# Allergic Inflammation: Effect of Propolis and Its Flavonoids

**DOI:** 10.3390/molecules27196694

**Published:** 2022-10-08

**Authors:** Nada Oršolić

**Affiliations:** Division of Animal Physiology, Faculty of Science, University of Zagreb, Rooseveltov trg 6, HR-10000 Zagreb, Croatia; nada.orsolic@biol.pmf.hr or norsolic@yahoo.com

**Keywords:** propolis, allergy, inflammatory cells, propolis sensitization, anti-allergic, anti-inflammatory, antioxidative effects of propolis

## Abstract

The incidence of allergic diseases and their complications are increasing worldwide. Today, people increasingly use natural products, which has been termed a “return to nature”. Natural products with healing properties, especially those obtained from plants and bees, have been used in the prevention and treatment of numerous chronic diseases, including allergy and/or inflammation. Propolis is a multi-component resin rich in flavonoids, collected and transformed by honeybees from buds and plant wounds for the construction and adaptation of their nests. This article describes the current views regarding the possible mechanisms and multiple benefits of flavonoids in combating allergy and allergy-related complications. These benefits arise from flavonoid anti-allergic, anti-inflammatory, antioxidative, and wound healing activities and their effects on microbe-immune system interactions in developing host responses to different allergens. Finally, this article presents various aspects of allergy pathobiology and possible molecular approaches in their treatment. Possible mechanisms regarding the antiallergic action of propolis on the microbiota of the digestive and respiratory tracts and skin diseases as a method to selectively remove allergenic molecules by the process of bacterial biotransformation are also reported.

## 1. Introduction

**Figure 1:**
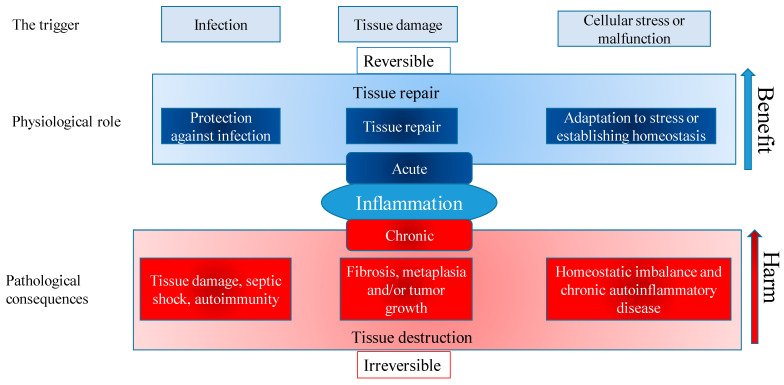
Inflammatory reaction: physiological and pathological consequences of inflammation.

The immune system is a complex system of organs and cells that are important in maintaining body homeostasis by protecting the organism from attacks of harmful microorganisms, and endogenously formed mutated cells, such as virus-infected or tumor cells. Protection from infectious diseases has both nonspecific and specific components [1]. The adaptive immune response provides specific protection against infection from bacteria, viruses, parasites and fungi and therefore, it is essential for normal health. Under normal physiological conditions, effector cells of the immune system remove antigens that induce an inflammatory response, preventing the onset of inflammatory responses and tissue damage. Tissue damage or cell death can occur during long-term uncontrolled inflammation. Inflammation is an adaptive response to stimuli and conditions, such as infection, tissue damage, and cellular stress or malfunction (Figure 1). The main characteristics of the local inflammatory reaction are the following: *dolor* (pain), *calor* (heat), *rubor* (redness), *tumor* (swelling) and *functio laesa* (loss of function). Systemic inflammation occurs in different conditions, such as massive trauma, chronic disease, or as a response to an infection, in which case it is designated as sepsis [2].

Controlled, physiological inflammation is a useful, adaptive reaction that plays a role in protection against infection, tissue repair and adaptation to stress, or establishment of “disturbed” homeostasis. However, if not regulated, it can become harmful and manifest as tissue damage, septic shock, autoimmunity, fibrosis, metaplasia, or homeostatic imbalance and chronic autoinflammatory diseases. Regardless of the cause, inflammation likely evolved as an adaptive response to maintaining homeostasis. Many pathological and clinical burdens of allergic disease reflect the long-term consequences of chronic allergic inflammation at sites of persistent or repetitive exposure to allergens [3].

Certain immune responses can give rise to excessive or inappropriate reactions. Disorders that result from excessive immune responses to allergens are known as hypersensitivity reactions. Allergens are antigens, commonly considered to be harmless, distinct from pathogenic organisms. Allergens may be environmental, such as dust, smoke, sulfur dioxide, diesel exhaust, and ozone, bee or other insect venoms, or food products (e.g., nuts, some propolis components, pollen, royal jelly), or certain proteins or components found in drugs. Host factors involved in the risk for allergic reactions are heredity, sex, race and age, nutrition, socioeconomic status and lifestyle of families, stress, allergen exposure, sibship size, childhood infections, dietary factors, and the environment (city or village). In addition to genetic factors, certain environmental factors, such as smoking, can act as adjuvants and can influence allergic symptoms, for example, allergic rhinitis/asthma. Furthermore, evidence suggests that interactions with microbes that inhabit the natural environment and human microbiome play an essential role in immune regulation. Changes in the microbiome of the host due to lifestyle, urbanization, changes in diet, excessive use of antibiotics and their effects on the intestinal microbiome also contribute to the increased risk of allergic diseases or lack of immunotolerance [4,5,6]. In developed countries, the incidence of allergies has increased significantly in the last 20 years among gender, age and racial groups, which consequently leads to a burden on the health system. Allergies primarily affect children and young people and, according to the World Allergy Organization, they affect 30–40% of the world’s population [6].

Allergy is a hypersensitivity reaction initiated by the immune response of our body occurring upon secondary contact with an antigen, producing a tissue injury that can cause serious disease. Hypersensitivity (hypersensitivity reaction or intolerance) is an unwanted reaction of the normal immune system that includes allergies and autoimmunity. Allergic reactions cause itching, pain, and swelling (edema) [5]. Allergic diseases can seriously affect vital functions of the organism and lead to psychological disorders of the patient, negatively affect the quality of life of the patient and the entire society; also, they can be a great economic burden.

Hypersensitivity reactions were classified into four types by Gell and Coombs (Table 1). Briefly, a Type I hypersensitivity reaction is mediated by the production of IgE antibodies. The reaction often occurs as an immediate allergic reaction to food and pollen, medicines, insect bites or as a serious anaphylactic reaction in patients who have previously been in contact with the corresponding allergen. In addition to IgE antibodies, the reaction is characterized by mast cell hypersensitivity, activation of helper CD4 T cells (Th2) and recruitment of eosinophils. Type II hypersensitivity reactions are cytotoxic reactions, involving IgM and IgG antibodies, specific for certain tissues in the body, and cause the destruction of cells in these tissues (e.g., autoimmune hemolytic anemia, Hashimoto’s thyroiditis and Goodpasture’s syndrome, erythrocyte transfusion reaction). The final result of this reaction is phagocytosis, namely the activation of killer cells or cell lysis mediated by complement activation. The activation of the complement system results in opsonization, agglutination of red blood cells, cell lysis, and cell death. Type III hypersensitivity reactions are mediated by immune complexes, with tissue damage caused by deposition of antigen-antibody complexes (e.g., many vasculitides and glomerulonephritis). Activated neutrophils and medium-sized immunocomplexes are the most important for damage to blood vessels of various tissues, especially the kidneys, the heart, and damage to arteries and joints.

Type IV hypersensitivity reactions (e.g., skin tests for TB, contact dermatitis) are delayed reactions that often occur after 72 h from exposure to the allergen and are mediated by sensitized T lymphocytes and macrophages. Distinct from true hypersensitivity reactions, which occur after sensitization, nonallergic hypersensitivity reactions (e.g., pseudo allergies) cause mast cell activation and histamine release after initial exposure to a trigger substance (e.g., radiocontrast media) [6].

Propolis is a resinous product of bees that they collect from the buds and bark of plants to construct the hive, protect it from the wind, bad weather, and enemies; it also protects the bee colony. Toxicity data for propolis are limited. Propolis is considered to be non-toxic, non-genotoxic, and safe at doses of 1.4 mg/kg, or about 70 mg/day. Specifically, over 90 days, a dose of 1400 mg/kg/day in mice did not cause histomorphological changes in mice and was proposed as NOEL (No-observed-adverse-effect level). It was also confirmed that the administration of ethanolic extracts of Brazilian and Chinese propolis in 5-week-old mice at a dose of 2230 to 4000 mg/kg for 2 weeks did not affect animal mortality, body weight, or histological abnormalities within the tissues. Furthermore, the epicutaneous application of propolis (20%, 10%, 1% in acetone) on the shaved flank of a guinea pig causes irritation after application of a dose greater than 20% solution. On the other hand, the LD50 for flavonoids has been confirmed, the LD50 varies from 2000 to 10,000 mg/animal (8000 to 40,000 mg/kg for a 250 g rat). However, propolis as a natural product may contain environmental pollutants including heavy metals. The analysis confirmed six toxic metals (Ni, Cr, Hg, Cd, Pb and Sn) in 106 samples of Brazilian raw propolis. Toxic metals, because they are not biodegradable, can accumulate in living tissues along the food chain reaching humans mainly through food (up to 90%). It is interesting that the process of filtration and separation of crude propolis reduces the presence of heavy metals by 24.24% to 100.00% [5,7].

Propolis and its components are important in health protection, prevention and treatment of minor diseases. Propolis is an integral part of (bio)cosmetics, and as a natural antibiotic in the treatment of ear, throat, and nose infections. Applications of propolis are very broad, spanning food industry, medicine (as an immunomodulator, healing of wounds and burns), cosmetics, and hygiene products. Despite the good properties of propolis, numerous studies have indicated that it causes allergic reactions in people hypersensitive to propolis components. Various patch test studies have shown various degrees of reaction to propolis and its components. Sensitivity to propolis was found in European studies (1.2–6.6%); a Finnish study in adults (0.5–1.4%), and children (2–13.7%), a Polish study in children (16.5%) and in young adults (5.4%), and a study in Prague (4%) [7]. The observed allergic reactions were the following: contact cheilitis, contact stomatitis, perioral eczema, labial edema, oral pain, peeling of lips, and dyspnea. Various allergens have been isolated from propolis, namely 3-methyl-2-butenyl caffeate, phenylethyl caffeate, benzyl caffeate, geranyl caffeate, benzyl alcohol benzyl cinnamate, methyl cinnamate, ferulic acid, and tectochrysin. In addition, propolis seems to be one of the most frequent contact sensitizers and should be included in routine patch testing in children and adolescents before prescribing it.

Propolis and its flavonoids have been widely used in folk medicine as anti-inflammatory agents, and as ingredients in anti-fungal antibiotics, anti-coagulants, anti-retrovirals, anti-allergic and anti-cancer drugs. Propolis has been used in folk medicine for a very long time. The anti-inflammatory action of propolis is based on inhibition of platelet aggregation, rat paw edema, and adjuvant-induced arthritis, eicosanoid synthesis, cytokines production, and other mediators important in controlling allergic diseases. Propolis is also known as a radioprotector; it has protective activity against gamma-radiation-induced DNA damage and UV radiation. In this way, propolis protects the skin from numerous processes, such as premature aging (wrinkles, scaling, dryness, dilatation of blood vessels, and loss of collagen) and skin cancer [8,9]. The wound healing effects of propolis and its ability to induce the production of type I and III collagen besides wound matrix degradation should be emphasized. Propolis contains a large number of compounds that accelerate skin healing processes, such as tensile strength and elasticity, and enables growth, expansion, and migration of human keratinocytes. These properties and biochemical changes in propolis could favor re-epithelization and thus represent promising effects in wound healing. It should be noted that the use of propolis as a dressing in wound healing also contributes to low cost, safe and painless application, prevention of infection, promotion of rapid wound healing, small number of dressing changes during healing, lack of scar tissue formation, and others. In a recent “back to nature move”, modern man is searching for natural products with medicinal properties, particularly those obtained from plants and bees, that have the potential ability to control allergies and/or inflammation. Laboratory and clinical research on propolis, phytochemicals and their flavonoids and other antioxidants suggest their use in prevention and treatment of a number of diseases, including allergies. This review summarizes the current knowledge regarding the mechanism(s) involved in generation of sensitivities and/or inflammation potential of polyphenolic/flavonoids components present in propolis as well as their effect in on the control of allergy diseases and possible modes of antiallergic reaction.

## 2. Risk Factors and Links between Allergy, Epidemiology and Immunology

Epidemiologic studies play a critical role in evaluating the causes of allergic diseases and are the primary scientific tool used to establish causality when experimental study designs are not possible.

IgE-mediated inflammatory reactions are directed towards foreign molecules called allergens. The prevalence of sensitization is higher in people who have an atopic predisposition, that is, a genetic predisposition to diseases in which IgE antibodies are produced in response to even a limited exposure to environmental triggers that usually do not bother other people. In Eastern Europe the prevalence of allergic sensitization is lower than in the West, possibly due to differences in living standards and exposure to various bacteria and viruses [1].

Genetic and environmental factors including air pollution, housing conditions and other lifestyle factors have been linked to the etiology and triggering of allergies [2]. Humbert et al. [3] found more similarities than differences between atopic and non-atopic asthma and hypothesized that immunologic factors play a significant role in both variants. According to new research, dietary factors, especially the amount and quality of dietary fat intake [4] (key components of a typical Western diet), significantly contribute to allergic sensitization [5,6,7]. The hygiene hypothesis states that allergies and increased longevity are both consequences of reducing infectious stressors during early childhood. The secretion of Th2 cytokines (IL-4, IL-5 and IL-13) during the intrauterine life of the fetus or in the early phase of the immature immune system of the newborn postnatally increases the production of allergen-specific IgE and eosinophilic inflammation. Conversely, infections with viruses and perhaps other intracellular organisms influence development of Th1 responses with production of Th1-like cytokines such as IL-12 and IFN-γ that downregulate Th2 responses. The hygiene hypothesis clearly shows the importance of the Th1/Th2 relationship; insufficient exposure to microbes and reduced Th1 response leads to an increase in the prevalence of atopic sensitization and clinical diseases such as asthma, allergic rhinitis, and eczema. The changing pattern of microbial exposure, with the decline in certain infectious diseases, may lead to a slower maturation of the immune system, with a delayed development of the optimally balanced immune responses.

The term “sensitivity” is general and may include true allergies, reactions that do not affect the immune system (and therefore are not technically allergies), and reactions for which the cause has yet to be determined. Sensitization to an allergen reflects the allergen’s ability to elicit a Th2-cell response, in which IL-4 and IL-13 drive IgE production by promoting immunoglobulin class-switch recombination in B cells. Factors influencing the likelihood to develop clinically significant sensitization are: host genotype, type of allergen, concentration of the allergen in the environment, route of allergen exposure, presence of other agents that may enhance the sensitization process as well as myeloid and/or plasmacytoid characteristics of the DC subpopulations that participate in the responses.

Certain non-allergic types of sensitivity are called intolerances and may be caused by toxins, enzyme inadequacies, drug-like chemical reactions, psychological associations, and reactions to chemicals found in food, water, medications, cosmetics, perfumes, textiles, building materials, and plastics. Examples of intolerances include lactose intolerance (inability to digest lactose due to a deficiency of lactase) and phenylketonuria (an autosomal recessive metabolic genetic disorder characterized by a deficiency in the hepatic enzyme phenylalanine hydroxylase). The identification of allergies and other sensitivities is a long-term and demanding process that requires the help of numerous experts.

**Figure 2:**
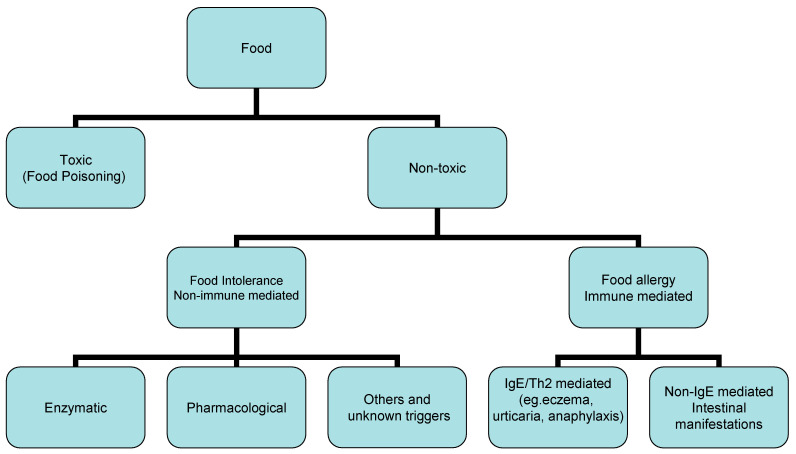
Adverse reactions to food.

There are immune-mediated and non-immune mediated reactions among adverse reactions to food. Immune-mediated reactions to food are mediated either by IgE antibodies or other immunological pathways, whereas food intolerance causes the formation of antibodies against proteins in a certain type of food. IgE antibodies, which would indicate an allergic process, are not formed. IgA antibodies are formed in the first phase, and after multiple stimulations, IgG antibodies are formed. Food intolerances also comprise non-immunologic-mediated responses that depend on enzyme deficiencies, pharmacological reactions or arise by unknown mechanisms. Adverse health consequences in children (aged 0 to 17 years) due to allergic reactions to food include food intolerances (Table 2, Figure 2). Thus, in the USA, 32 million Americans have food allergies, and relevant studies, published in 2018 and 2019, estimated that the number of Americans of all ages who had allergic symptoms to a certain food were: 8.2 million: milk; 6.1 million: peanuts; 6.1 million: tree nuts; 3.9 million: eggs; 2.6 million: fish; 2.6 million: wheat foods; 2.4 million; and soy: 1.9 million. In developed countries, IgE-associated food allergies affect 3–8% of children and 1–3% of adults. Food allergies have increased worldwide from about 3% of the population in 1960 to about 7% in 2018. Approximately 7.6% of children in the USA are thought to have food allergies, and 40% of them have multiple food allergies [10]. The food we eat daily can have both allergy-promoting and anti-allergic nutrients, which indicates the possibility of allergy control through food selection [11]. For example, certain vitamins (A, C, D and E), minerals (selenium, copper, zinc and magnesium), probiotics and omega-3 polyunsaturated fatty acids+ (PUFAs), as well as polyphenols, have been shown to possess anti-allergic properties, whereas omega-6 PUFAs are precursors for leukotriene C4, which is known to promote allergic inflammation.

Food allergens induce the production of IgE antibodies that have the ability to bind to the surface of mast cells and basophils distributed throughout the body (Figure 2), stimulating their degranulation (release of histamine from basophils and mast cells). which cause an allergic response in a sensitive person within a few minutes or, at most, a few hours. Chemically, food allergens are proteins or glycoproteins with molecular masses from ~10,000 to 70,000 daltons, and high stability to proteases and heat [12].

Approximately 200 of the hundreds of thousands allergenic proteins have been isolated from food [13] and include peanuts, soybeans, crustacea, fish, cow’s milk, eggs, tree nuts and wheat. According to Hefle et al. [14] these allergens make 90% of all reported food allergies worldwide [10,11,12,13,14,15].

The number of people suffering from IgE-mediated allergic diseases is constantly increasing and reached the 6th leading cause of diseases in the US [10,16]. Although the number of Americans with allergies is around 40–50 million according to the National Institute of Health, it is believed that a large number of people have not been diagnosed yet. The overall prevalence of certain allergies, such as asthma, allergic rhinitis and atopic eczema, are increasing substantially [16]. According to the World Health Organization, the number of patients who have asthma is about 300 million, and it is expected to increase to 400 million by 2025. Similarly, allergic rhinitis occurs in more than 500 million people worldwide and also is constantly increasing. According to certain studies, an increase in the prevalence of allergic diseases can be attributed to lack of microbial stimuli during infancy and early childhood. Thus, the intake of probiotics can increase the effectiveness in reducing eczema by 21%.

Dietary changes over the past 20–40 years seem to be contributing to the increased incidence of allergic diseases in both children and adults, and it is estimated that one in two people in Europe will likely suffer from at least one allergy by 2021 (Global Allergy and Asthma European Network, GA2LEN). In addition to hereditary and environmental factors, breastfeeding, the intake of certain nutrients and probiotics seem to be key contributors in the development of allergies and asthma in children. Intake of antioxidants in diets, such as polyphenols (flavonoids, vitamin C, vitamin E and selenium coming mainly from fruit and vegetables), may have a protective effect [17]. Furthermore, different fats found in milk, butter, vegetable oils and fish may have different effects on the development of allergies and asthma. Recent research points to a central role of the microbiome, which is highly influenced by multiple environmental and dietary factors. It was found that extensive changes in the environment and nutrition produce dysbiosis in the gut, skin and lung microbiome, inducing qualitative and quantitative changes in their composition and metabolic activity. Dysbiosis induces qualitative and quantitative changes in the microbiota that directly affect immunological mechanisms leading to allergic diseases (Table 3). The role of probiotics and prebiotics in the diet is promising. Therefore, it seems that the microbiota is a very dynamic environment, influenced by multiple environmental and nutritional factors, with a complex role in allergic diseases. For example, raw propolis contains 45.02–58.72% of dietary fiber and a significant amount of sodium, potassium, copper, zinc, iron, calcium, potassium, phosphorus, manganese, magnesium, pyridoxine and folic acid, which indicates that it can be used as food with a positive effect on intestinal microbiota. Foods rich in fiber and antioxidants, such as polyphenolic constituents of plants and bee products including propolis, stimulate the activity and formation of “good bacteria”, reducing and acting as a bactericidal on pathogenic bacteria; and can reduce and prevent numerous allergic reactions. The bifidogenic effect of bee products has been known for a long time and can be useful in treating allergic reactions [17,18]. The main effects of probiotics are presented in Table 3.

Living organisms, such as probiotics, appear to protect against the development of allergies by inducing changes in the gut bacteria that stimulate the immune system. For example, Japanese cedar pollinosis affects nearly one in six Japanese. Oral administration of *Bifidobacterium longum* BB536 and *Lactobacillus casei* strain Shirota and casei have been shown to be effective in relieving Japanese cedar pollinosis symptoms during the pollen season [19].

Food protein allergies are quite rare and affect approximately between 1–2.5% of adults and 6–8% of children in the US. Moreover, the allergenicity of food or protein varies a lot in different cultures depending on exposure to the allergen, such as rice in Japan or peanuts in North America [20].

## 3. Development of Allergic Inflammation

Allergy is an overreaction of the immune system to normally harmless substances and is accompanied by an inflammatory reaction. Allergic inflammation consists of three phases. Early-stage reactions (or type I immediate hypersensitivity reactions) are induced within seconds to minutes after allergen induction, and late-stage reactions occur within a few hours. In contrast, chronic allergic inflammation is a permanent inflammation that occurs at sites of repeated exposure to allergens [3].

IgE immunoglobulins are allergen-specific and are produced by B-cells after the sensitization process. Cytokines, such as interleukin (IL)-4 and IL-13, are crucial in the transition of B-cells from IgG to IgE production. By binding to high-affinity IgE receptors (FcεRI), IgE immunoglobulins trigger the release of mediators from mast cells. Sensitized individuals already have allergen-specific IgE bound to surface IgE receptors on mast cells. The main receptors involved in mast cell activation are: FcεRI, Toll-like receptors, complement receptors (CR1–5) and IgG receptors FcγRI (CD64) and FcγRII (CD32) [21]. Cross-linking of adjacent IgE molecules by bivalent or multivalent allergen, FcεRI aggregation initiates a complex intracellular signaling process that results in the secretion of key biologically active products: (1) mediators stored in primary cytoplasmic granules; (2) mediators formed from lipids and newly synthesized cytokines, chemokines and growth factors, as well as other products. Mediators contribute to an allergic reaction, known as an “immediate hypersensitivity reaction”. Certain genes are involved in the specific immune response (i.e., HLA-D, TCR, CD14, toll-like receptor, STAT6) and Th1/Th2 cell differentiation; others are genes encoding (total) IgE response or IgE receptor functions (i.e., IL-4, IL-4R, FcεRIβ, Fc epsilon RI) and genes involved in the inflammatory process (TNF-γ, IFN-γ, IL-3) [3,20,21].

**Figure 3:**
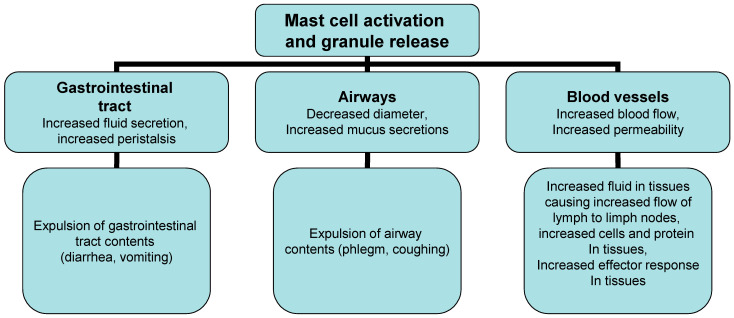
Effector mechanism in allergic reaction.

Two phase reactions trigger the IgE/allergen cross-linking event in acute and chronic inflammation. The first process depends on calcium as the most important trigger, but also the main switch of the cascade allergic reaction and is based on the release of previously formed mediators such as histamine, interleukins, serotonin and Hagemann’s factor from mast cells and basophils (Figure 3). The released chemical mediators are responsible for allergic (inflammatory) processes, such as vasodilation, increased vascular permeability and increased chemotaxis of other inflammatory cells (Figure 3). The second phase of the reaction begins with the synthesis of mediators derived from lipids through the conversion of phospholipids into arachidonic acid by phospholipase A2, followed by the conversion of arachidonic acid into leukotrienes (LT), platelet activation factor (PAF) and prostaglandins [3,20,21]. The inflammatory cascade continues with the synthesis of LTB4 and LTC4 (with their metabolic derivatives LTD4 and LTE4), prostaglandin D2 and other substances that stimulate by acting on vascular smooth muscles, connective tissue, mucous glands and inflammatory cells. Mast cells further increase inflammation through the production of numerous cytokines including TNF-β, IL-4, IL-5, IL-13 [3,22]. Their number is increased in the late phase of inflammation, where mast cells attract leukocytes, especially Th2 cells and eosinophils, by induction and regulation of adhesion molecules on the endothelial cells of blood vessels. Cytokines (IL-4, IL-5 and IL-13) are also secreted by memory Th cells of the Th2 type reactivated by allergens that are presented by local antigen-presenting cells (APC) in the airways, stimulating an inflammatory reaction. Symptoms characteristic of allergic rhinitis are the result of previously formed lipid mediators on the mucous membrane of the upper respiratory tract, and not a property of the allergen itself. Certain patients react to certain allergens, while others do not; reaction to allergens depends on a number of factors including genetics, geography and exposure levels [3,20,21,22].

The late phase of the reaction showing clinical features of inflammation after 6–12 h is based on an increase in the arrival of inflammatory cells in the tissue, especially eosinophils, neutrophils, basophils, macrophages and lymphocytes. The key task of cytokines secreted from mast cells is to: (1) recruit other immune cells either directly or indirectly (TNF-α,LTB4, IL-8 CCL2 and other chemokines); (2) activate innate immune cells (TNF-α and IL-5); (3) affect many aspects of DC biology, T cells and B cells (IL-10, TNF-α, TGF-β, histamine); (4) stimulate the reaction of Th2 cells (IL-4 and IL-13), (5) promote the inflammatory reaction (TNF-α, and IL-3), and (6) stimulate the production and activation of eosinophils (IL-5 and GM-CSF) [3,20,21,22]. Certain mast-cell-derived products can also influence the biology of structural cells, including vascular endothelial cells, epithelial cells, fibroblasts, smooth muscle cells and nerve cells while other molecules, such as IL-10, TGF-β, can have anti-inflammatory or immuno-suppressive functions. Leukocytes recruited in late-phase reactions consist of Th2 cells in the early phases of the response and Th1 cells in the late phases; they release cytokines that attract and activate granulocytes, monocytes and eosinophils, contributing to the inflammatory reaction and damage to the surrounding tissue, especially after the reaction of eosinophils with IgE and their degranulation.

Chronic allergic inflammation occurs when exposure to allergens is constant or repeated and this persistent inflammation is associated with changes in the structural cells at the affected sites, and in many cases with markedly altered function of the affected organs. Thus, it is known that chronic allergic inflammation in atopic dermatitis (AD), allergic rhinitis, and asthma is associated with tissue remodeling, which includes long-term changes to the structural elements of the affected sites (such as increased vascularity) and substantial alterations in the barrier function of the affected epithelia including increased risk of both cutaneous infections with the bacterium such as *Staphylococcus aureus* and the colonization or the development of nasal polyps in patients with allergic rhinitis. Chronic inflammation is based on a complex interaction of many cells including mast cells, T cells, eosinophils, basophils, neutrophils, monocytes/macrophages, platelets, NK cells (natural killer T-cells), as well as a number of cytokines, including IL-4, IL-5, IL-12, IL-13, IL-15, IL-25 and IL-33 [3,23,24].

### 3.1. Cells and Their Mediators Involved in the Inflammatory Process

The inflammatory process begins with the release of various mediators from the resident cells, including granulocytes (neutrophils, mast cells, eosinophils), lymphocytes, macrophages, DCs, platelets, interstitial fibroblasts, and vascular endothelial cells. This signaling changes the local adhesion molecules’ profile and creates a chemotactic gradient that recruits cells from the blood stream to the inflammatory site.

#### 3.1.1. Neutrophils

Neutrophils, the main cell type in most acute and some chronic inflammatory diseases, are the body’s first line of defense in the innate arm of the immune system. Neutrophils dominate the early stages of inflammation as cells with antimicrobial functions and cells which can interact, directly, or via cytokines and chemokines, with other immune cells to modulate both innate and adaptive immune responses. Their granules contain numerous microbicidal agents; they can destroy invading microorganisms, through phagocytosis and intracellular degradation, release of granules, and formation of neutrophil extracellular traps after detecting pathogens. Several studies have shown that neutrophils can regulate the induction of allergic inflammation through their innate responses. Neutrophils are a major source of oxidative stress through their respiratory chain; they are the main source of superoxide anions (O_2_^−^), H_2_O_2_ and HOCL which, together with neutrophil proteases, increase the degree of tissue damage. In addition, neutrophils may contribute to allergic inflammation by increasing microvascular permeability, inducing proinflammatory cytokines, elastase, cathepsin G, and proteinase-3, IL-8, lactoferrin, myeloperoxidase (MPO), matrix metalloproteinase 9 (MMP-9), and secreted polymeric mucins MUC5AC [3,20,21,22,23,24]. It has been shown that neutrophil elastase causes MUC5AC mucin synthesis via the EGF receptor, ERK and NF-kB pathways in A549 cells [24]. Activated neutrophils have a role in the allergic sensitization process and thus, may contribute to allergic inflammation in allergic rhinitis and asthma by priming T cells and attracting eosinophils.

#### 3.1.2. Monocytes/Macrophages

Monocytes/macrophages are a heterogeneous cell population acting as a bridge between the innate and adaptive immune systems. Macrophages and neutrophils migrate into extravascular tissue by adhesion and diapedesi in the early acute phase of inflammation. Macrophages exert a variety of pro- and anti-inflammatory functions that are correlated with various states of immune activation and are broadly classified into M1- or M2-related classes. Macrophage plasticity is influenced by environmental factors; M1 polarization is characterized by the expression of Th1 and Th17 cells, while in M2 polarization the Th2 response is dominant. M1 macrophages highly express CD80, CD86, MHCII, TLR4, and iNOS, and produce high levels of pro-inflammatory Th1 cytokines (e.g., IL-6, IL-12, IL-1β, and TNF-α) and chemokines (e.g., CCL2, CCL5, MCP-1, MCP-2, IL-8) while M2 macrophages are characterized by high expression of MRC1, CD163, Arg-1 and low expression of iNOS, MHCII and CD86. M2 is generally assumed to play significant roles in wound healing, tissue remodeling, parasite clearance, and resolution of excessive inflammation.

These important immune effector and homeostatic macrophage functions are altered during allergic reactions, and activated macrophages can contribute to the propagation of the inflammatory process by producing a number of inflammatory cytokines, chemokines, complement cascade proteins (C1, C2,C3, and C4), and eicosanoids [e.g., prostaglandin (PG)E2, thromboxane (TX)A2, prostacyclin (PGI2), LTB4, and PAF], procoagulant factors such as tissue factor (TF) and plasmin inhibitor (or alpha 2-antiplasmin). Macrophages are characterized by the presence of numerous receptors on their surface, such as scavenger receptors, TLR, opsonin receptors, including the mannose receptor for recognizing glycoproteins on the surface of microbes, CR1, CR3, CR4, Fcγ RI, Fcγ RII, and Fcγ RIII [3,22,25].

#### 3.1.3. Eosinophils

Eosinophils play a key role in the symptoms of asthma, allergies, and atopic and adverse drug reactions. Eosinophils can regulate local immune and inflammatory responses, and their accumulation in the blood and tissue is associated with several inflammatory and infectious diseases [26,27]. When activated, eosinophils rapidly produce and secrete four distinct granule cationic proteins: major basic protein, eosinophil peroxidase, eosinophil cationic protein and eosinophil-derived neurotoxin, and inflammatory mediators including GM-CSF, and RANTES, which exert an autocrine effect on eosinophil survival, differentiation, and accumulation. In addition, eosinophils secrete a range of highly toxic granule proteins and other mediators of inflammation presented in Table 4**.** Eosinophils play an essential role in the development and maintenance of allergic diseases through the secretion of lipid mediators and proteins and may be an important target for treatment and management of allergic diseases. Eosinophils have an important role in late-phase allergic reactions, where they are recruited in high numbers. Recruitment of eosinophils is a complex and dynamic process involving cell adhesion and attraction, diapedesis, and chemotaxis by cytokines such as IL-5 and eotaxin which can regulate eosinophil homing and tissue accumulation in allergic asthma [26,27]. Eosinophils express seven integrin heterodimers [28]: α4β1 (CD49d/29), α6β1 (CD49f/29), αMβ2 (CD11b/18), Lβ2 (CD11a/18), αXβ2 (CD11c/18), αDβ2 (CD11d/18), and α4β7 (CD49d/β7). Rapid apoptosis of eosinophils in the absence of their growth factor IL-5 was observed in the tissues. The IL-5 production by Th2 cell located in the airway mucosa stimulates the migration of eosinophils from the blood and increases their survival by inhibiting apoptosis. The main biological effects of mature eosinophils are considered to occur in tissues, where they may release active mediators upon activation, including proinflammatory cytokines, arachidonic acid-derived mediators, enzymes and reactive oxygen species (ROS) (Table 4). The release of toxic substances by activated eosinophils may contribute to the pathophysiology of eosinophilic disorders, including allergic asthma and rhinitis. Eosinophils also express a number of Ig receptors, including receptors for IgE that are involved in activation and release of prestored toxic granule proteins involved in the destruction of the epithelial cell layer that is characteristic for allergic asthma. In autoimmune diseases, eosinophils have a number of functions, including: (1) tissue damage by cytotoxic granule proteins; (2) antibody-dependent cellular cytotoxicity; (3) activation of tissue remodeling and fibrosis; (4) antigen presentation; (5) modulation of the adaptive immune response; (6) promotion of B cell responses; and (7) induction of tissue repair processes. Eosinophils can induce a protective immune response against helminths, viral and bacterial pathogens. Eosinophils are a source of anti-tumorigenic (e.g., TNF-α, granzyme, cationic proteins, and IL-18) and protumorgenic molecules (e.g., pro-angiogenic factors) depending on the milieu.

#### 3.1.4. Basophils

Basophils, the least abundant granulocyte population (less than 1%) of all circulating leukocytes, are initiators, regulators and effectors of type 2 inflammation. Type 2 inflammation, a specific type of immune response, which includes Th2 cells that secrete IL-4, IL-5, and IL-13 and stimulate type 2 immunity, which is characterized by high levels of immunoglobulin E (IgE) and eosinophils. In addition to the accumulation of eosinophils, there is an accumulation of other cells such as basophils, mast cells, Th2 cells, type 2 innate lymphoid cells (ILC2s) and IgE-producing B cells and their cytokines. An excessive response of eosinophils within the airways leads to airway remodeling and causes smooth muscle cell changes (hyperplasia and hypertrophy), mucus cell changes (hyperplasia, metaplasia) and vascular remodeling (more blood vessels and more leaky vessels). Such changes create a predisposition of the mucous membrane of the respiratory tract for an exaggerated response to inhaled allergens or environmental stimuli, such as viruses, cigarette smoke or other air pollutants. Murine basophils are characterized by the presence of surface markers (FcεRI+, CD49b+, CD69+, Thy-1.2+, CD123+, CD200R+, CD117−, CD19−, CD14−, CD122−, CD11c−, Gr-1−, NK1.1−, B220−, CD3−, γδTCR−, αβTCR−) 2, 8, 9, 11, 12 that can also be found in humans. Basophils can be activated by a variety of signals, including cytokines such as IL-3, IL-5, IL-33, IL-18, and GM-CSFs, antibodies (IgE, IgD, and IgG), allergens, proteases, TLRs ligands, and complement factors (C5a) (Table 5) [3,29]. Numerous TLRs receptors are present on human basophils such as TLR1, TLR2, TLR4, TLR6 and TLR965–67 while murine basophils express TLR1, TLR2, TLR4 and TLR668. In addition to TLRs, human basophils have also been shown to express the complement receptors CR1, CR3, CR4 and CD88. Substances that lead to degranulation of mast cells/basophils are: (1) At/Ag interactions (IgE, IgG); (2) complements (C3a, C4a, C5a); (3) HRF (histamine release factor). When activated, basophils release mediators (Table 5) such as histamine, leukotriene (LT)C4, platelet-activating factor (PAF), all major mediators of acute bronchoconstriction, chemokines, and cytokines (IL-4, IL-5, IL-13, and thymic stromal lymphopoietin). In addition, basophils play an important role in allergic reactions, through B-cell activation, by increasing the production of IL-4 and B-cell-activating factor and inducing production of immunoglobulins IgM, IgG, and IgA, mediated by IgD. Basophils are involved in both IgE-dependent and IgE-independent allergic inflammation. In IgE-dependent allergic inflammation, basophils are activated by antigen and IgE stimulation, causing degranulation and secretion of cytokines. Basophil-derived mediators induce the recruitment of other inflammatory cells, leading to the chronic allergic inflammation in antigen-challenged sites. IgE-independent allergic inflammation mediated by basophils is mostly associated with thymic stromal lymphopoietin (TSLP). TSLP-elicited basophils are activated by cytokine stimulation, such as IL-3, IL-18, and IL-33, leading to the secretion of IL-4 [23,29]. Basophil-derived IL-4 induces the recruitment of inflammatory cells, including eosinophils, to the site of inflammation. In addition, basophils play a major role in the regulation of monocytes and macrophages in M1 or M2 macrophages under the influence of environmental factors [29]. Thus, in the early phase of inflammation, basophil-derived IL-4 acts on skin-resident cells, including fibroblasts, endothelial cells, and ILCs, leading to the accumulation of eosinophils to the site of inflammation while in the later phase of inflammation, basophil-derived IL-4 acts on inflammatory monocytes, leading to the differentiation into M2 macrophages. M2 macrophages promote either the resolution of allergic inflammation or protective immunity against helminthic parasites [29]. Finally, basophils have been shown to act as antigen-presenting cells for T_H_2 differentiation in response to protease allergens and may act independently of or cooperate with DCs and other professional APC populations [29].

#### 3.1.5. Mast Cells

Mast cells are effector cells in the regulation of numerous processes, including the regulation of immunity, inflammation, crossing the blood–brain barrier and cancer growth [20,21,22]. Mast cells play an important role in the early phase of allergic reactions due to their localization and rapid encounter with environmental or food allergens in the submucosa of the respiratory or digestive tract. Changes in the surrounding tissue such as swelling, itching, sneezing in allergic rhinitis are attributed to the secretion of histamine. Alongside pro-inflammatory cytokines (IL-1, IL-6, IL-8 and TNF-α), upon stimulation, mast cells secrete number different mediators (Table 6), including biogenic amines serglycin proteoglycans, serine proteases, and various other enzymes and growth factors; these molecules can be associated with the granules, such as tumor-necrosis factor-α (TNF-α) and vascular endothelial growth factor A (VEGFA). Mast cells activated by the aggregation of FcεRI also release an abundance of arachidonic acid metabolites, notably leukotriene (LT) C4, prostaglandin (PG) D2, and platelet activating factor (PAF) [30]. The main classes of mediators that are released by mast cells are presented in Table 6. Mast cells contribute to the expression and synthesis of cytokines depending on: (1) the maturation of mast cells; (2) the location of mast cells within different tissues [31]; and (3) cytokine activation of mast cells [31].

Mast cells produce Th2 cytokines, such as IL-4 and IL-13, which are increased in AD. These cytokines facilitate the development of skin infections by inhibiting the production of antimicrobial peptides through keratinocytes [20,21,22]. Furthermore, the cytokine profile of mast cells is crucial in their physiological and pathological role in innate and acquired immunity [20,21,22], inflammation [21,30,31], wound healing and tumor growth [21]. In addition to cytokines, mast cells secrete proinflammatory, vasoactive, and neurosensitizing molecules. Regardless of the activation mechanism, vasoactive, proinflammatory, and neurosensitizing molecules act on keratinocytes, endothelial cells, or nerve endings to stimulate the release of additional molecules leading to chronic inflammation and neuropathic hypersensitivity or pain [32,33]. Chronic stress weakens immune processes, while acute stress stimulates these processes. In addition to the pathological activation of mast cells via FcεRI, under physiological conditions, mast cells play a protective role in host defense against bacteria through the production of tumor necrosis factor (TNF)-α, mainly as a result of Toll-like receptor (TLR)4- or CD48 (a mannose-containing GPI-anchored molecule)-mediated activation [32].

#### 3.1.6. Lymphocytes

Lymphocytes comprise three cell types: natural killer (NK) cells as effector lymphocytes of the innate immune system, B cells, and T cells as part of the adaptive immune response, either in humoral immunity or in cell-mediated immunity [3,23,24,34,35,36,37,38]. During inflammation, there are close interactions between lymphocytes and macrophages and their cytokines, in a bidirectional way, which leads to a persistent inflammatory response. Allergen-reactive T helper cells type 2 (Th2) are thought to play an important role in the induction and maintenance of the allergic inflammatory cascade through increased production of cytokines and chemokines (GM-CSF, IL-4, IL-5, IL-6, IL-9, IL-10, IL-13, macrophage-derived chemokine). The reaction of other cells activated by Th2 cytokines, and the reaction of damaged tissue associated with Th2 (eotaxin, transforming growth factor-β, IL-11), contributes to pathophysiological allergic disorders. Increased production of cytokines and other factors consequently leads to the increased production of IgE antibodies, recruitment or activation of mast cells, basophils, and eosinophilia, mucus hypersecretion, subepithelial fibrosis, and tissue remodeling. Cytokines produced by Th2 cells induce differentiation, activation, and in situ survival of eosinophils (through IL-5); promote the production of high amounts of antibodies by B lymphocytes, including IgE (through IL-4 or IL-13); as well as the growth of mast cells and basophils (through IL-4, IL-9, and IL-10) [35,36]. Certain cytokines inhibit several macrophage functions or the development of Th1 cells, which are important against the majority of infectious agents, with the exception of some gastro-intestinal nematodes. Thus, the phagocyte-independent Th2 response is usually less protective than the Th1 response. However, Th2 cells likely play an important regulatory role in the immune system because switching from Th1 to Th2 may provide a protective effect when the Th1 response threatens to become a dangerous for the host.

T-regulatory (Treg) cells play a key role in maintaining immune tolerance to allergens. Knowledge about their role in the regulation of infectious, autoimmune diseases, asthma and allergen immunotherapy has increased in the last few years; however, the mechanisms by which Treg cells fail to maintain tolerance in patients with allergic diseases are not fully understood. Many different Treg subsets have been described [37,39], including CD8+ Treg cells, NK cells and several different CD4+ Treg cell subsets. Major populations of natural Treg cells (nTreg) are generated in the thymus but can also develop from conventional CD4+ cells in specific conditions or signals. These cells are known as CD4+ FOXP3+ nTreg cells. Inducible Treg cells are a second population. CD4+ FOXP3+ iTreg cells arise extra-thymically in peripheral lymphoid tissues after exposure to antigens and in the presence of TGF-β. These cells are found in the gastrointestinal tract and the lungs during chronic inflammation. It should be noted that iTreg cells are less stable than nTreg cells, regardless of the fact that they share similar levels of markers, such as FOXP3, CTLA-4, GITR, ICOS, CD103 and CD25. In addition, iTreg can lose FOXP3 expression and secrete cytokines such as IFN-γ and IL-17 under inflammatory conditions. The third population represents CD4+ type 1 T regulatory cells (Tr1) that have been defined by the expression of IL-10 and the surface marker LAG-3 and CD49b when FOXP3 and CD25 expression is absent. Tr1 cells express a number of transcription factors common to other T cell populations including Ahr (Aryl hydrocarbon receptor), and others. Th3 are CD4 T lymphocytes are induced from naïve CD4 T cells by TGF-β, with a similar phenotype to conventional Treg cells that secrete TGF-β and IL-10 and are characterized by the expression of IL-4. The suppressive role of Treg cells is mediated by multiple mechanisms from the release of inhibitory cytokines (TGF-β, IL-10, and IL-35) and cytolytic molecules (granzymes A and B), or the reduction in the function of antigen-presenting cells (cytotoxic T lymphocyte antigen 4 (CTLA-4) and lymphocyte activation gene 3 (LAG-3) to withdrawal of trophic cytokines (IL-2 via CD25) and modulation of metabolic pathways (CD73 and CD39).

In the suppression of effector Th2 cells by nTreg cells, the key cell surface molecules are CTLA-4, Notch-3 and LAG-3 and the intracellular enzyme heme oxygenase (HO)-1. CTLA-4 and CD28 deliver opposite signals to APCs, thereby regulating indoleamine 2,3-dioxygenase (IDO) activity and its anti-proliferative effects on effector T-cells. The suppressive effects of nTreg cells are abrogated by GITRL and interleukin (IL)-6 that can be expressed by APCs. Immature or semi-mature APCs producing IL-10 or transforming growth factor (TGF)-β generate T-regulatory cell type 1 (Tr1)- and Th3-type of adaptive CD4+ T cells that promote immune tolerance (aTreg cells), respectively. The production of these cytokines by APCs can be induced by regulatory-type pathogen-associated molecular patterns (PAMPs). Data suggest that nTreg cells also mediate the development of aTreg (Tr1, Th3) cells.

In addition, other Th lymphocytes have also been used to characterize allergic diseases: Th9 lymphocytes, Th22 lymphocytes, T follicular helper cells (Tfh) lymphocytes and invariant natural killer T (iNKT) lymphocytes [34,35,38]. Th17 cells, characterized by the secretion of IL-17A (also called IL-17), IL-17F, IL-22 and other cytokines, can induce autoimmunity by promoting tissue inflammation and mobilizing innate immunity. Abnormal Th17 immunity may also contribute to the pathogenesis of classically recognized Th2-mediated allergic disorders. Th9 cells have an important role in the immune response regulation. Th9 express predominantly IL-9. IL-9 causes the induction of lung eosinophilia, increased serum total IgE levels, airway hyperreactivity, and generation of cytokines from active mast cells; it also upregulates high-affinity IgE receptors on mast cells. Th22 cells are positive for chemokine receptors CCR4, CCR6 and CCR10 and produce mostly IL-22. High levels of IL-22 have been found in patients with allergic rhinitis and asthma. T follicular helper cells (Tfh) represent a specialized CXCR5-expressing CD4 T cell population, regulated by Bcl-6 in child and adult asthma patients. In addition to the mentioned T cells, a part of unconventional T lymphocytes is also involved in the pathophysiology of asthma, especially iNKT and mucosa-associated invariant T cells (MAIT) [35,38] that produce low to moderate levels of IL-4 and IL -13. MAIT-17 can produce several cytokines, including IL-17 and IFN-γ [35], which are considered to be associated with asthma symptoms in children.

#### 3.1.7. Plateles

Platelets are small cellular fragments deriving from megakaryocytes that maintain homeostasis, rapidly respond to vascular injury, become activated, and induce platelet aggregation and thrombus formation. Activated platelets and platelet-activating factor (PAF), as a potent inflammatory mediator released by inflammatory cells such as macrophages, neutrophils, eosinophils, platelets and endothelial cells, play an important role in the pathogenesis of inflammatory diseases through the induction of inflammatory mediators (Table 7) that: (1) stimulate vascular endothelial cells to produce cytokines (e.g., IL-1 and IL-18) and platelet expression of CD40/CD40 ligands to promote increased thrombotic activity; (2) stimulate the synthesis of lipid mediators (TXA2, hydroxy-eicosatetraenoate (12-HETE) and PAF); (3) release peptide 2 (activates neutrophils), sphingosine 1-phosphate, growth factors (PDGF and TGF-β), nitric oxide (NO), cytokines (IL-1β and IL-7) and chemokines (CXCL5, MCP-3, RANTES, MIP-1α, platelet factor 4); (4) proinflammatory mediators such as histamine, serotonin; (5) growth-regulating oncogene α (GRO-α); (6) high-mobility group box 1 and P-selectin. The expression of CD40/CD40 ligand on activated platelets is crucial in antigen presentation to effector cells (T lymphocytes), maturation and activation of DCs and production of T-dependent isotype switching as well as interaction between other immune cells such as B cells, T cells, neutrophils, macrophages, endothelial cells, NK cells and DCs [30,40]. PAF plays an important role in inflammatory and thrombotic diseases and in both immune-mediated and non–immune mediated anaphylaxis. It is a neutrophil chemoattractant, which increases eosinophilic and neutrophilic infiltration, platelet aggregation and activation through the release of vasoactive amines in an inflammatory reaction leading to vascular permeability, nasal hyperreactivity to histamine, bradykinin or kinins, circulatory collapse, decreased cardiac output, bronchoconstriction, mucus hypersecretion, and inflammation of bronchi. As a signaling molecule, it plays a role in inflammatory diseases such as asthma, allergic rhinitis, sepsis, atherosclerotic disease, liver cirrhosis, inflammatory reactions in the skin and malignancy. Its production requires phospholipase A2 and acetyltransferase while its degradation is catalyzed by PAF acetyl-hydrolase (PAF-AH). PAF is rapidly hydrolyzed and degraded to an inactive metabolite, lysoPAF; and its elimination time is short (~3–13 min). Platelets have the ability to modulate early inflammation as well as the link between inflammation and coagulation. Platelets are the main source of serotonin that is released during allergic inflammatory responses. It should be emphasized that platelets also contain substances that limit inflammation through the production of lipoxin during the interaction of platelets and leukocytes. They play an important role in the adaptive immune response through the expression of high and low affinity receptors for immunoglobulins (FcγRI, FcγRII, FcγRIII, FcεRI, FcεRII, FcαRI, etc.). Increased levels of blood markers of activated platelets (PF4, P-selectin, β-thromboglobulin (β-TG), and PMP) have been observed in numerous human diseases, including systemic lupus erythematosus (SLE), systemic sclerosis (SSc), and small vessel vasculitis, AD and psoriasis, but also in a mouse model of AD. Some of the platelet factors including histamine, 5-HT, acid proteases, IL-1β, TGF-β, PAF and prostaglandin E2 induce pruritus and bleeding in AD [39,40,41]. The interaction of platelets with the endothelium and leukocytes leads to the activation of platelets and increased deposition of fibrin by platelets, as well as stimulation of neutrophils in the release of chromatin and formation of extracellular traps (NETs), which directly promote activation and aggregation of platelets [42].

#### 3.1.8. Epithelial Cells

A healthy epithelium is key to maintaining mucosal homeostasis and may potentiate immune tolerance to frequently encountered allergens. Specialized epithelial subset cells, including secretory and ciliated cells, are important tissue barriers whose dysfunction is a fundamental component of chronic human inflammatory diseases, including allergies. Epithelial cells at mucosal surfaces play a dominant role in allergic diseases and have an active role in the inflammation process. In susceptible individuals, after exposure of the epithelium to environmental allergens, mucosal epithelial cells release cytokines, such as IL-1, IL-25, IL-33, thymic stromal lymphopoietin (TSLP) and GM-CSF, and endogenous danger signals, such as uric acid, ATP and HMGB1. These factors lead to the production of inflammatory mediators and activate a network of DCs and other innate immune cells including basophils and innate lymphoid cells type 2 (ILC2) through the signaling pathways of nuclear factor kappa B (NF-ĸB) and IĸB [43]. Inflammatory mediators of epithelial cells include cytokines (e.g., TNF-α and IL-1β), chemokines (e.g., IL-8, MIP-2, CXC chemokines (MCP-1, IL-7 and IL-15), adhesion molecules [e.g., β-integrins and intercellular adhesion molecule 1 (ICAM-1)], O, and various TLR (2 and 4) receptors, growth factor receptors, TNF-α receptors (TNFR1 and TNFR2), and plasminogen activator receptors [43,44,45]. The relative importance of each mediator appears to differ in various models of Th2 inflammation. For example, IL-33 mediates its Th2-promoting effect via signaling to various immune cells, such as mast cells, T cells, DCs; and ILC2 might be a potent driver of IL-5 and IL-13 in mast cells and ILC2. IL-25 secreted from epithelial cells can activate DCs by upregulation of co-stimulatory molecule expression (CD80, CD86), which promotes the differentiation of naïve T cells into Th2 lineage. IL-25 also enhances collagen deposition in the lungs by promoting IL-13 from ILC2 while IL-13 and ILC2 is necessary for worm clearance in the lamina propria. It has been shown that epithelial cells in the airways can react to IL-25 through an autocrine-paracrine mechanism, stimulating the production and secretion of leukocyte chemoattractants CCL5 (RANTES) and CXCL1 (GROa). In addition, IL-25 plays a role in viral-exacerbations of asthma. Elevated TSLP production from epithelial cells of the human airways, gut and skin is observed in multiple allergic diseases such as AD, asthma, and food allergies. Alongside Th2 differentiation, TSLP can promote Th9 cells and production of IL-9 leading to allergic airway inflammation. TSLP can induce the differentiation of splenic progenitor-like cells resembling granulocyte-monocyte precursors (GMP) into different APC cells of the myeloid lineage.

#### 3.1.9. Endothelial Cells

Pathogenesis of chronic allergic diseases, such as bronchial asthma, allergic rhinitis, eosinophilic gastrointestinal disorders and AD, involves chronic inflammation and tissue remodeling caused by immune reactions to various antigens on the tissue surface. Due to their anatomical location, vascular endothelial cells are the final responders that interact with various exogenous antigens and pathogen-associated molecular patterns (PAMPs) that come into contact with the epithelial surface. Endothelial cells line the inner walls of blood vessels, forming a selective permeable barrier between the blood inside the vessels and the surrounding tissues. Vascular endothelial cells are the final responders to interact with various alarmins on the epithelial surface which spontaneously express MHC-I molecules and a wide variety of functional PRRs, including TLRs, and NLRs. Endothelial cells form a single-cell-thick layer called the endothelium, which lines the inner wall of blood vessels. The endothelium, as a selective permeable barrier between the bloodstream and vessel walls in physiological conditions, is composed of 1 − 6 × 10^13^ cells and covers more than 1000 m^2^ of the entire body surface. The main functions of the endothelium are: (1) maintenance of metabolic homeostasis, (2) regulation of blood vessel hemodynamics, (3) regulation of cell permeability, coagulation and extravasation. Endothelial cells are crucial in the regulation and propagation of the inflammatory response and the development and exacerbation of allergic disorders.

Inflammatory conditions and increased oxidative stress by inflammatory cells lead to the disruption and opening of interendothelial junctions, increasing the migration of inflammatory cells across the endothelial barrier. This migration of inflammatory cells leads to tissue injury in addition to removing pathogens and foreign particles [46]. Activated endothelial cells contribute to inflammation by secreting numerous inflammatory molecules, including inflammatory cytokines (TNF-α, IL-1α, IL-1β, IL-6 and IL-8), chemokines (IL-8, RANTES and MIP-1), ROS and RNS, adhesion molecules [ICAM-1, ICAM-2, PECAM, vascular cell adhesion molecule 1 (VCAM-1), E-selectin and P-selectin], growth factors (VEGF and TGF-β), proteases, leukotrienes (LTs), and prostaglandins (PG). IL-33 can stimulate pulmonary microvascular endothelial and epithelial cells via the IL1RL1/ST2 receptor and stimulate type 2 cytokines, the production of CXCR2 chemokine (IL-8) as the main neutrophil chemoattractant and proangiogenic factor. Endothelial cells play a key role in innate and adaptive immune responses, expressing cytokine and chemokine receptors (TFNR1 and IL-1R), TLRs, and procoagulants and protease-activated receptors. During inflammation, changes in the cytoskeleton and intercellular proteins in these cells are crucial for vascular permeability and leukocyte migration [46,47].

#### 3.1.10. Fibroblasts

Fibroblasts, a heterogeneous population of stromal cells, are recognized for their structural role in synthesizing (e.g., collagen and fibronectin, proteoglycans, etc.) and remodeling the ECM in tissues. Fibroblasts are mainly responsible for ECM production, but also for the reparative role of damaged tissue through collagen synthesis. ECM remodeling via fibroblasts takes place through the production of metalloproteinase (MMP) and tissue inhibitors of MMP (TIMP) and communication with nearby cells. High levels of MMP2 and IL10 in patients with allergic contact dermatitis and AD are associated with an impaired Th1/Th2 cell ratio. It was demonstrated that there is a bidirectional interaction between eosinophils, mast cells and fibroblasts via numerous soluble factors (growth factors, cytokines and MMPs) which, by activating fibroblasts, leads to the development of tissue remodeling/fibrosis. Prolonged or repeated activation of both mast cells and eosinophils can lead to their extended survival, recruitment of other inflammatory cells and further secretion of inflammatory mediators resulting in tissue damage [48]. Eosinophils also affect collagen production depending on the source of fibroblasts and collagen deposition by MMP-9, TIMP-1 and TIMP-2. Collagen production increased in skin fibroblasts but decreased in lung fibroblasts. Numerous mediators of mast cells and eosinophils could be responsible for the antagonistic or synergistic effects on fibroblast functions. Thus, under certain conditions, there is an excessive and unbalanced repair process, which eventually leads to fibrosis and tissue remodeling. High concentrations of cytokines (TGF-β, IL-4 and TNF-α) from mast cells can stimulate the synthesis of collagen and fibronectin in fibroblasts, contributing to fibrosis that disrupts the structure and function of organs. TNF-α is a growth factor that stimulates fibroblast proliferation and chemotaxis and secretion of MMPs, collagenase and cytokines, including TNF-*α*, IL-6, and IL-1β [49]. Crosstalk between fibroblasts and innate and adaptive inflammatory cells directly promotes fibroblast activation by cytokines and growth factors and indirectly leads to myofibroblast activation via further induction of pro-inflammatory, pro-fibrotic factors in other immune cells [48].

#### 3.1.11. Dendritic Cells

Based on ontogeny, DCs consist of heterogeneous groups and can be classified in the following subsets: conventional dendritic cell (cDC), plasmacytoid dendritic cell (pDC), monocyte-derived dendritic cell (moDC), and Langerhans cells. DCs are primary antigen-presenting cells involved in interactions with T cells leading to the proliferation of Th1 or Th2 cell types. The role of DCs in inducing or preventing allergic inflammation involves different mechanisms of activity, ranging from antigen sampling and DC migration to complex interactions with other cells, infectious agents and allergens. DCs play a central role in allergic inflammation; they are the link between allergens and the environment, epithelial cells, and immune effector cells. A number of signals trigger the activation of quiescent and immature DCs into mature DCs, including: danger signals (TNF-α,IL-1β, type I IFN, ATP and UTP, heat-shock proteins-HSPs, necrotic cells), innate immunity maturation signals (TLRL, viral RNA and poly IC) Mycobacterial extracts, single strand RNA, CpG deoxy-oligonucleotides, bacterial DNA) adoptive immunity maturation signals (CD40LAg-Ab complex), and others (growth factors GM-CSF, cytokines IL-15, IL-17, TSLP) [50]. DCs internalize and process antigens and then display them on the surface, in conjunction with human leukocyte antigen molecules, allowing for the “presentation” to the lymphocytes, resulting in their activation. It has been confirmed that aberrant DC expression of surface receptors leads to Th2 polarization and significant changes in the expression of important molecules to interact with T cells, such as costimulatory molecules of the B7 family (CD80, CD86, PD-L2/B7-DC, ICOS-L), members of the TNF family (CD137/4-1BBL, CD134/OX40L, CD70) and chemokine receptors (CCR5, CCR7). DC function can be directly or indirectly regulated by inflammatory cells and their cytokines. Activated DCs actively produce and release numerous cytokines and chemokines during inflammatory responses. These products contribute to the recruitment of inflammatory cells to the site of inflammation, including differentiation of cells of the mononuclear phagocytic system into different subsets of tissue macrophages with additional specific functions leading to tissue damage and the development of chronic allergies independent of the role of DC cells in antigen presentation. Monocyte-derived DCs are key players in both innate and adaptive immunity, given their antibacterial capacity as well as their ability to stimulate CD4+ helper T-cells through antigen presentation, and induce (Th)1, Th2, Th17 and T regulatory cells. A few studies reported higher FcεRI expression on DCs in individuals suffering from atopic diseases, such as allergic rhinitis, AD, and asthma [51].

### 3.2. Oxidative Stress in Allergic Disorders

Oxidative stress occurs in many allergic and immunologic disorders, such as asthma, rhinitis, and and inflammatory skin diseases such as AD, urticaria, psoriasis and other skin diseases. Although ROS serve as cell signaling molecules for normal biologic processes, excessive exposure to ROS and nitrogen species can also provoke damage to multiple cellular organelles and processes [52]. ROS can react with proteins, lipids, and nucleic acids, leading to cell death. After reaction with lipids, the lipid structure is destroyed, permeability increases and cellular death occurs, leading to tissue damage and necrosis. Furthermore, arachidonic acid (AA) is a precursor of enzymatic and non-enzymatic oxidized products, such as prostaglandins, thromboxanes, leukotrienes, lipoxins, and isoprostanes, which play an important role in asthma. These products may exert signaling or damaging roles during physiological and pathological conditions, some of them being markers of oxidative stress linked to inflammation. ROS attack proteins to form carbonyls and can react with nitrogen species and tyrosine to form nitrotyrosine whereas reaction with DNA can form base pair derivatives, such as 8-oxo-2-deoxyguanosine [52,53]. DNA damage caused by ROS induces the release of 8-oxoguanine (8-oxoG) from damaged DNA. The damaged base is cleaved by 8-oxoguanine-DNA glycosylase-1 (OGG1); and 8-oxoG and OGG1 form a signaling complex that activates NF-kB and inflammation.

An imbalance between ROS and antioxidants can lead to elevated oxidative stress levels. Inflammatory cells (mast cells, monocite/macrophages, eosinophils and neutrophils) and airway tissue cells (epithelium and smooth muscle) are the likely source of reactive radicals and produce endogenous ROS after their activation by a variety of stimuli. Given the location of mast cells at the host-environment interface, such as perivascular areas and mucous membranes where they encounter antigens and invading pathogens, mast cells play a key role in allergic inflammation, host defense, as well as in coordinating the early stages of certain allergies and autoimmune diseases. FceRI cross-linking in mast cell causes degranulation and releases wide range mediators, such as histamine, prostaglandin D2, certain cytokines, chemokines, and tryptase, followed by allergic inflammation. Moreover, FceRI cross-linking results in ROS production within mast cells. It is known that inflammatory cells, especially monocytes, macrophages and neutrophils, as defense cells of innate immunity, create high levels of ROS and inflammation via NADPH oxidase and myeloperoxidase (MPO) through a “respiratory burst”. Furthermore, key cells in allergic inflammation, such as mast cells, generate high levels of ROS in response to antigens; therefore, an imbalance between ROS and antioxidants occurs in allergic diseases. Apart from mast cells, eosinophils are the most dominant inflammatory cells in both allergic and non-allergic asthma, AD, and allergic rhinitis; and they have greater ability to synthesize ROS than neutrophils. In addition, the activity of NADPH oxidase is higher in eosinophils than in other phagocytes, although activated macrophages possess a powerful ROS production system [52,53]. Circulating inflammatory cells (peripheral blood monocytes and eosinophils) might also be a source of ROS and allergic inflammation. Monocytes are activated to secrete superoxide when IgE binds to membrane receptors. Thus, in atopic dermatitis, the inflammation process may be increased by oxidative stress. AD is a chronic, itchy skin disorder caused by a combination of genetic predisposition, immune dysregulation, and damage to the skin barrier. The source of oxidative stress may be irritants, environmental and food allergens, which bind to the AhR and induce the production of ROS, DNA damage and the production of inflammatory cytokines that cause skin inflammation. Increased oxidative stress in the skin leads to skin barrier dysfunction or immune dysregulation. Another source of oxidative stress may be skin microbes such as skin colonization with *Staphylococcus aureus* or defective host defense mechanisms involved in controlling bacterial infection. The presence of a bacterial pathogen stimulates the synthesis of IL-4 and IgE to cause dermal inflammation and therefore, itching and scratching. It seems that monocytes from patients with AD are primed to generate ROS and that oxidative stress and redox imbalance may develop or worsen AD by inducing pruritus or enhancing Th2 polarization. In addition, increased levels of IgE, cytokines IL-4, IL-4 receptor and IL-13, or altered cutaneous inflammation, such as mast cell chymase, are visible. Namely, by colonizing inflamed skin via TLR ligands, *Staphylococcus aureus* converts transient dermatitis mediated by Th2 cells into permanent and aggravated chronic inflammation [54]. Furthermore, mast cells generate mainly intracellular ROS following the aggregation of FcεRI, which may act as secondary messenger in the induction of several biological responses. Oxidative stress can activate nuclear factor kappa-B (NF-*κ*B) pathways to activate gene expression and synthesis of antioxidant enzymes and proinflammatory cytokines, such as IL-6, IL-8, IL-9, and IL-33, which in turn enhances dermal inflammatory infiltrate and histamine release in the affected skin to worsen symptoms [55,56].

In psoriatic skin, reactive species are generated by keratinocytes and activated leukocytes, mostly neutrophils and macrophages, which play a key role in inducing psoriasis-like skin disease [54,55,56]. Lactoferrin released by specific neutrophil granules can promote neutrophil-endothelial cell adhesion and, as a source of iron, may promote the Fenton reaction with the generation of the hydroxyl radical (OH•) [54]. Psoriatic skin is characterized by an advanced state of lipid peroxidation [54] and depletion of intracellular GSH [54]. In psoriasis, ROS produced during the inflammatory process affects primarily the polyunsaturated fatty acid in a biological system, forming a lipid peroxidation product MDA, which serves as an important biological marker of lipid peroxidation. The increased levels of other reactive species, such as nitric oxide (NO), superoxide anion (O_2_•−) and hydrogen peroxide (H_2_O_2_) have been found in the skin of psoriatic patients [54,55,56]. Hydrogen peroxide (H_2_O_2_) and the superoxide anion (O_2_•−) can be generated by the action of the enzyme xanthine oxidase, which displays a higher activity in psoriatic epidermis [54,55,56]. Additionally, cytokines such as tumor necrosis factor alpha can contribute to H_2_O_2_ production [54]. This suggests that cellular redox status plays a pivotal role in healthy skin environment and that an imbalance between pro-oxidant and antioxidant mechanisms could result in skin diseases, including psoriasis.

Airway hyperresponsiveness is closely related to the redox and immunological status of asthma patients. The oxygen stress level in asthma is increased because of inflammatory cells in vivo, and cigarette smoke (CS) or particulate matter (PM), which are a major component of air pollution. It has been suggested that ROS play an important role in pathogenesis of airway inflammatory diseases [55], especially increased concentrations of NO^●^, H_2_O_2_ and 8-isoprostane. Some radicals such as O_2_•−, NO^●^, and halides interact to form highly reactive species such as peroxynitrite and HOBr, which in turn cause nitration and bromination of protein tyrosine residues. Hypohalous acids, strong oxidants and important factors in host defense systems are produced by activated neutrophils, monocytes, eosinophils, and possibly macrophages (HOCl) and activated eosinophils (HOBr). HOCl and HOBr are formed by the reactions of H_2_O_2_ with Cl^−^ or Br^−^ catalyzed by the heme enzymes, myeloperoxidase (MPO) and eosinophil peroxidase (EPO). Both HOCl and HOBr readily react with biological molecules, including amino acids, proteins, antioxidants (including thiols), carbohydrates, lipids, and DNA. These oxidants also destroy ciliary functions of the respiratory epithelium and decrease surfactant activity while increasing mucus secretion, activity of cytokines and proteases, neutrophil chemotaxis and alveolar permeability and smooth muscle contractility. Oxidative stress may also reduce glutathione (GSH) levels and cause the inactivation of antioxidant enzymes such as superoxide dismutase, with a consequent increase in apoptosis, shedding of airway epithelial cells and airway remodeling [52,53,54,55,56]. Based on the above, the use of antioxidants, such as polyphenolic/flavonoid components of propolis, may be crucial in inhibiting the production of ROS and activation of mast cells, eosinophils, macrophages and other cells including their cytokines involved in allergic inflammation.

## 4. Botanical Sources of Propolis

Propolis (bee glue) is a resin-like material collected by honeybees from the buds of various plant sources and used by bees to seal holes in honeycombs, smooth out the internal walls, protect the entrance of beehive against intruders and protect bees from diseases. Propolis also serves as a means of better maintenance homeostasis of the nest environment by reducing the growth of microbes on the hive walls, preventing uncontrolled air flow into the nest, waterproofing the walls from external moisture. The collected materials are mixed with the enzyme α-glycosidase present in the bees’ saliva, partially digested and added to beeswax to form the final product (raw propolis, propolis in natura). Raw propolis is mostly composed of 50% plant resins, 30% waxes, 10% essential and aromatic oils, 5% pollens and 5% other organic substances % waxes, 10% essential and aromatic oils, 5% pollens and 5% other organic substances such as vitamins (A, C, D, E and B1, B2, B5, and B6), niacin and folate, and some micro and macro minerals. More than 500 compounds have been identified in propolis from different geographical origins including phenolic acids, flavonoids (flavones, flavanones, flavanols, dihydroflavanols, and chalcones), terpenes, lignans, amino acids, fatty acids, vitamins, and minerals [57,58,59,60,61,62]. The chemical characteristics of propolis are linked to the diversity of geographical locations, plant sources and bee species. The chemical composition of propolis has been summarized in articles, with respect to geographical location, botanical origin and bee species [57,58,59,60,61,62]. The main chemical components of propolis confirmed in different percentages are as follows: fatty and aliphatic acids (24–26%); flavonoids (18–20%); sugars (15–18%); aromatic acids (5–10%); esters (2–6%); vitamins (2–4%); alcohol and terpenes (2–3.3%); microelements (0.5–2.0%); others (21–27%) [60,61,62].

Propolis contains about 30 elements. Calcium, manganese, zinc, copper, silicon, iron and aluminum are present in the largest quantities [60,61,62]. Several vitamins can be found in propolis including B vitamins, vitamins C, D, P and E, as well as provitamin A (β-carotene). In addition to vitamins, small amounts of enzymes are also present in propolis, among which are α-amylase, β-amylase, α-lactamase, β-lactamase, maltase, esterase, and transhydrogenase [62]. Enzymes in propolis come from bee glands, while part of the enzymes come from pollen [60,61,62]. The protein content in EEP is on average about 2.8% [57,58,59,60,61,62], while the content of free amino acids is low and about 17 amino acids can be found [57,58,59,60,61,62]. Among the amino acids, pyroglutamic acid was discovered, which belongs to the bee organism. Polysaccharides, as well as di- and mono-saccharides are present in propolis: sucrose, glucose, fructose, rhamnose, ribose, talose and gulose [57,58,59,60,61,62]. There are different types of propolis according to the location of its botanical origin and collecting season (Table 8). Despite the high diversity, most studies list at least six main chemical types of propolis: (1) propolis found in Europe, North America, and non-tropical regions of Asia whose primary source is poplar (*Populus* spp., most commonly *P. nigra*); (2) propolis present in Russia, where birch (*Betula verrucosa* Ehrh.) is dominant; (3) green propolis is present in Brazil, mainly from *Baccharis* spp., predominantly *B. dracunculifolia* DC.; (4) in Cuba and Venezuela, there is red propolis, propolis belonging to *Clusia* spp.; (5) ‘Pacific’ propolis from Okinawa and Taiwan, no identified plant source; as well as (6) ‘Canary’ propolis from the Canary Islands. “Poplar propolis” the resinous exudates of the buds of poplar trees (*Populus* species), are characterized by similar chemical composition; the main compounds belong to the phenolic class and include flavonoid aglycones, aromatic acids, and their esters. Brazilian green (Alecrim) propolis, is characterized by the presence of prenylated derivatives of *p*-coumaric acid and *o*-hydroxy-acetophenone [62]. Chemical constituents of Brazilian propolis, are not present in propolis from other regions. It differs from European propolis by different flavonoid constituents, diterpenes and lignans. Brazilian green propolis contains relatively higher amounts of artepillin C (20.7%), bacharrin (7.5%) and kaempferide (3.6%). *Clusia nemorosa* (Clusiaceae) is main plant source of red propolis in Cuba, while in Venezuela bees collect propolis most often from *C. scrobiculata.* Cuban propolis is different from both European and Brazilian propolis and it is rich with polyprenylated benzophenones. For propolis produced in the Pacific region, geranyl flavanones are the characteristic compounds, which are also found in propolis from the African region [60,62]. Propolis, independently of its plant source and chemical composition, always possesses antimicrobial, antioxidative and immunomodulatory, anti-inflammatory, anti-allergic, dermato-protective, laxative, anti-diabetic, anti-angiogenic and antitumor activity. The antimicrobial activity of propolis is attributed to the presence of flavonoids such as pinocembrin, galangin, pinobanksin, pinobanksin-3-acetate, and caffeic acid ester. Propolis with strong antioxidant activity contains compounds such as kaemferol, caffeic acid (CA), and phenethyl caffeate. The anti-inflammatory activity can be explained by the presence of active flavonoids such as quercetin, acacetin and naringenin and cinnamic acid derivatives including baccharin, drupanin and caffeic acid phenethyl ester (CAPE). CAPE, as a major constituent of temperate propolis, has numerous biological activities, including inhibition of nuclear factor κB (NF-κB); inhibition of cell proliferation; induction of cell cycle arrest and apoptosis. Different components of Brazilian, Cuban and Mexican propolis were found to exert pro- and anti-inflammatory effects depending on the dose, which may be useful for the development of novel immunomodulatory drugs.

The concentration of bioactive principles in propolis may vary substantially according to the origin of the sample, and such differences may affect its biological activities. The compound characteristics of propolis from different geographical origins are listed in Table 8.

Healing properties of propolis have been established in folk medicine since antiquity. Several results confirm the therapeutic efficacy of propolis through quantitative and qualitative analyses of collagen types I and III expression and degradation in matrix of wounds, showing that propolis supports re-epithelization along with its antioxidant and anti-inflammatory properties. Propolis and its flavonoids successfully inhibit the synthesis of nucleic acids, attachment and formation of biofilms and energy metabolism of bacteria, prevent bacterial cell division and cause dysfunction within the cytoplasm, thereby inactivating bacterial activity and growth. At the same time, they stimulate growth of skin tissue and regeneration as well as modulate collagen production accelerating wound repair and regeneration; these take place through numerous mechanisms involving a number of cellular and molecular processes involved in integrated phases, such as hemostasis, inflammation, cell proliferation and tissue remodeling [63,64]. Propolis reduces oxidative stress in wounds by inducing the expression of antioxidant related genes (hemeoxygenase-1 (HO-1), glutamate-cysteine ligase- modifier GCLM and- catalytic GCLC subunits) and improves collagen expression and cell viability on cells exposed to high levels of oxidative stress. It was also demonstrated that propolis and some of its active substances have a pronounced cytostatic [64,65,66,67,68] anticarcinogenic and antitumor effect both in in vitro and in vivo tumor models [64,65,66,67,68]. Honeybee propolis and its components (CA, CAPE, artepilin C, quercetin, naringenin, resveratrol, galangin, genistein and other) are the most promising as antitumor and anti-inflammatory agents [5,57,58,64,65,66,67,68]. This strategy opens a new venue for cancer prevention to cancer treatment as well as treatment of numerous diseases associated with oxidative stress and inflammation, including allergy diseases.

In addition to their antitumor properties, flavonoids present in propolis are powerful antioxidants and anti-allergic nutrients that inhibit mast cells, basophils, and eosinophils regulate allergic inflammation, inhibit the synthesis and release of Th2 type cytokines such as interleukin (IL)-4 and IL-13, the release of chemical mediators, histamine, and CD40 ligand expression by high-affinity immunoglobulin E (IgE) receptor-expressing cells, such as mast cells and basophils [18,69]. Flavonoids inhibit the release of histamine, leukotrienes, prostaglandin D2, and GM-CSF from mast cells in a concentration-dependent manner [69,70,71]. They also inhibit IL-4-induced signal transduction and affect differentiation of naïve CD4+ T cells into effector T-cells through their inhibitory effect on activation of the aryl hydrocarbon receptor. An epidemiological study of 10,054 adults in Finland clearly indicated an association between flavonoid intake and the risk of several chronic diseases including the incidence of asthma, which was significantly reduced by higher intake of quercetin, naringenin and hesperetin [72].

## 5. Role of Propolis and Its Components in Allergy

### 5.1. Propolis Components as Sensitizers

Propolis components can cause allergies with pronounced clinical manifestations, depending on the route of sensitization; allergies include contact dermatitis, airborne allergic contact dermatitis, urticaria, rhinitis, conjunctivitis, angioedema and anaphylactic shock. Propolis allergy has been well documented throughout the literature and its frequency varies between 1.2–6.55% [70,71]. Giusti et al. [73] reported that 5.9% of patch tested children showed positive allergic reactions to propolis. Less than 10% of the population is thought to be sensitive to propolis (sensitization rate, 1.2 to 6%); however, a significant increasing trend in sensitization has been observed in adolescents (from 2 to 13.7%) in the period 1995 to 2002 [74]. Different degrees of allergic reactions were shown in patch test studies, as reported by De Groot [75]. In the European study, the reaction level was in the range 1.2–6.6%; in the Finnish study in adults, the reaction ranged from 0.5 to 1.4% in adults, and 2–13.7 in children; in Polish children it was 16.5% and in young adults 5.4%, whereas in the research conducted in Prague, the percentage of allergic reactions was about 4%. In general, a higher frequency of allergic reactions was observed in the countries of Central and Eastern Europe (Austria, Germany, Poland, Lithuania, the Czech Republic, and Switzerland) where propolis is used as a biopharmaceutical. In addition, high frequencies of sensitization were also observed in the US and Canada from 2007 to 2008 (4.9%) and the United Kingdom in 2007 (3.5%). According to reference [75], the studies after 2002 show a higher rate of sensitization in the elderly, and in most reports the prevalence is much higher than 3%. In addition, it was reported that up to 28.6% of beekeepers were sensitized to propolis, probably due to their higher exposure to it [76]. According to Uter et al. [77], between 2007 and 2010, 2.35% of patch tested people were found to be allergic to propolis compared to 3.94% from 2015 to 2018, which is an increase of 68%.

Despite the chemical composition of propolis being variable and strictly dependent on the vegetation of a particular area at the place of collection, the main ingredients are flavonoids, phenolic acids and their esters, as well as terpenoids and steroids. In addition to numerous beneficial effects, propolis is also a potential allergen. Of the 800 propolis compounds discovered up to the present, the strongest sensitizers are caffeic acid esters (phenylethyl caffeate and methyl butenyl caffeate), but ingredients such as free aromatic acids, isoferulate, and flavonoids can be involved in allergic reactions to propolis [57,70,71,73,74,75]. Most of these products also occur in bud exudate of poplar species [75] known to be bees’ major source of propolis, at least in Central Europe. These compounds can be grouped as follows: (1) free aromatic acids (e.g., benzoic, caffeic, cinnamic, coumaric, ferulic acid); (2) esters of these acids (benzyl, methyl butenyl, phenethyl, cinnamyl and others); (3) flavonoids; (4) chalcones and dihydrochalcones; (5) terpenoids; (6) others (sugar, ketones, alcohols). From an allergologic perspective, the most interesting among these natural products are the esters of aromatic acids. Although the chemical composition of propolis is highly variable, about 26 components are thought to cause a severe allergic reaction, and the major sensitizers are widespread and found in many other cross-reactive products of plant origin.

Apparently, esters of CA play an essential role in propolis sensitization (contact dermatitis, oral mucositis with ulceration) [70,71,75,76,77,78]. Hausen et al. [78] reported that the primary allergen in propolis is ‘LB-1′, consisting of a mixture of 3-methyl-2-butenyl caffeate (54%), 3-methyl-3-butenyl caffeate (28%), phenylethyl caffeate (8%), CA (1%) and benzyl caffeate (1%); the strongest sensitizer is 3- methyl-2-butenyl caffeate. The widely accepted opinion, however, is that ‘LB-1′ is a 1,1-dimethylallyl caffeic acid ester [70,71,75]. From a structural point of view, the ester of CA plays an essential role. All tested caffeates showed moderate to strong sensitizing capacity. Isoprenyl caffeate present in propolis composition was indicated as a pro-hapten, which can be enzymatically oxidized in the skin cells and present as an allergen for T cells [70,71,75]. Other constituents of propolis (e.g., isoferulates, flavonoid aglycones, free aromatic acid) may contribute to the sensitizing properties to a lesser extent, depending on their variable amounts (or presence at all) in different propolis samples. The sensitizing potency seems to rise with the length of the aliphatic side-chain. Several observations support the assumption that the sensitizing capacity of caffeates is related to the presence of free hydroxyl groups on the aromatic ring. This would be in accordance with a new hypothesis suggesting that mono- and/or di-hydric alkylbenzenes form free-protein reactive radicals, which act as haptens. The results from Hansson et al. [79] support the hypothesis that the catecholic propolis hapten is a pro-hapten that forms a complete antigen after oxidation to caffeate quinone and addition to nucleophilic groups of proteins. Delayed allergenic contact dermatitis is usually caused by low molecular weight haptens. Haptens couple to macromolecules, presumably proteins, to yield a complete antigen. Considering the incidence of propolis sensitivity in humans, propolis causes allergy in only 1.2–6.55% [75,76,80]; 50% of those being sensitized acquired their contact allergy from cosmetics containing propolis and 20% from capsules used for propolis therapy.

On other hand, a group of German investigators examined the sensitizing capacity of 26 compounds known to be present in propolis and/or poplar buds and divided propolis compounds according to their sensitization ability as follows: (1) compounds with strong sensitizing abilities (3-methyl-2-butenylcaffeate, geranyl caffeate, farnesyl caffeate, benzyl caffeate, and benzyl isoferulate; (2) compounds with moderate sensitizing ability (coniferyl benzoate, resorcinol monobenzoate, cinnamyl caffeate, methyl caffeate, coniferyl alcohol, phenethyl caffeate acetate, and phenethyl isoferulate; (3) other flavonoids in poplar-type propolis, however, were experimentally found to have weak sensitizing ability (rhamnetin, quercetin, naringenin, acacetin, chrysin) or no such ability (galangin, kaempferol, apigenin, tectochrysin, ermanin) [81]. In a similar study, phenethyl caffeate was found to be a strong sensitizer, benzyl salicylate a moderate one, and benzyl cinnamate a very weak sensitizer [70,71,75,82].

On the other hand, there are few reports on propolis antigenicity on the oral mucosa [75]. It has been shown that propolis can cause allergic stomatitis accompanied with labial edema, dysphonia and dyspnea after topical use of a propolis solution and lozenges [83]. Contact cheilitis has been reported in some patients and oral mucositis with ulceration in a patient who used propolis lozenges daily after dinner. This case report emphasizes the antigenic potential of propolis and suggests that it should be carefully used as a topical agent in the treatment of oral diseases [5,83,84,85,86]. Moreover, it highlights the fact that delayed propolis contact sensitivity should be taken into account in differential diagnosis of oral lesions. Thus, Brailo et al. [81] and Henatsch et al. [87] reported an interesting case of delayed contact sensitivity that developed on the lips and the oral mucosa as a result of topical propolis use for treatment of recurrent aphthous ulceration. In contrast, numerous studies show a protective effect of propolis in induced mucositis, especially in patients who received treatment for tumors [87,88,89,90] and in cases involving dental health [90,91,92,93].

In certain cases, a few side effects, such as cutaneous eruption, lung dysfunction, allergy, and contact dermatitis were reported after propolis use [70,85]. Other authors suggest that propolis and its components are effective in controlling allergic diseases because many antioxidants inhibit histamine release from mast cells [69,70,71]. The results of patch testing with propolis in consecutive dermatitis patients were reported [75]. The epidemiological findings by Strom et al. [94] indicated that genistein (flavonoid present in propolis) might play an important role in allergic reactions especially during infancy when soy formula instead of cow milk formula is used. Soy formula should not be recommended for the prevention of allergy or food intolerance in infants at high risk of allergy or food intolerance [95].

### 5.2. Control of Allergy Reaction by Propolis and Related Flavonoids

Propolis has been widely used in folk medicine as an antioxidative and anti-inflammatory agent. Propolis has attracted the interest of researchers to elucidate its biological properties and discover new therapies to treat many diseases such as diabetes, tumors, bacterial infections, allergic rhinitis, and ulcers. Propolis inhibited platelet aggregation, eicosanoid synthesis and adjuvant arthritis, showing potent anti-inflammatory activity [70,71]. Kiderman et al. [96] reported the beneficiary effects of propolis treatment in an infant with eosinophilic ulcers. Recently, some studies [90,91,92] showed that propolis has significant antimicrobial properties in the saliva of patients with periodontal disease [90,91,92,93] whereas diprenyl-4-hydroxy-cinnamic acid, 3-prenyl-4-dihydrocinnamoloxy-cinnamic acid and 22-dimethyl 6-carboxy-e-thenyl-2H-1-bezopyran were confirmed as the main antimicrobial compounds with the highest activity against bacteria. Propolis has the ability to reduce dentinal hypersensitivity (sudden sharp short pain arising from tactile, osmotic, thermal or other stimuli from exposed dentin) by decreasing hydraulic conductance of dentin. In addition, there is considerable evidence indicating that flavonoids in propolis and plants, depending on their structure, can inhibit secretory processes, mitogenesis, and cell–cell interactions including their possible effects on adhesion molecule expression and function. Moreover, flavonoids may affect gene expression, proinflammatory cytokines and cytokine receptors. Antioxidant and radical scavenging activity of propolis and flavonoids contributes to the control of allergic reactions and inflammatory processes [8,9,70,71,97,98]. Several studies have demonstrated that flavonoids can inhibit the degranulation of mast cells, reduce histamine, tryptase, IL-6 and IL-8 release from cultured mast cells and macrophages [70,71]. Furthermore, several flavonoids have the ability to inhibit the release of histamine, leukotrienes, prostaglandin D2, IL-4, IL-13 and GM-CSF from mast cells and activated human basophils in a concentration-dependent manner [11,69,70,71,99,100,101,102]. All of the previous results suggest that flavonoids may have potent anti-inflammatory and anti-allergic activities, especially for mast cell related allergic inflammatory diseases, including allergic rhinitis asthma, AD, skin and gastrointestinal allergies [11,69,70,71,99,100,101,102,103].

#### 5.2.1. Anti-Allergic Effects of Propolis and Its Flavonoids

Propolis and its compounds are effective in controlling allergic diseases because many antioxidants inhibit histamine release from mast cells and basophils [69,70,71,99,100,101,102,103]. Mast cells, as long-lived resident cells, may be induced by immunological mechanisms or chemical agents. Mast cells and basophils expressing the high affinity IgE receptor (FcεRI) play an important role in allergic inflammation by releasing chemical mediators, such as histamine, heparin, serine proteases, cytokines, chemokines, prostaglandin, leukotrienes and PAF. After IgE-mediated degranulation, mast cells can undergo regranulation, a process that is important in terms of exacerbation and duration of allergies [69,70,71].

Contrary to the allergenic properties of propolis previously described, a few authors demonstated that propolis does not interfere with the immunologic process of mast cells degranulation. Mirsadraee et al. [103] and Khayyal et al. [104] demonstrated the potential beneficial effects of a propolis food product as an adjuvant in asthmatic patients. In particular, the ethanolic solution of propolis achieved a better effect than the aqueous solution in preventing the degranulation of mast cells. A possible explanation lies in the fact that the ethanolic solution contains a significantly higher level of flavonoids (in Croatian propolis the ratio of flavonoids between the ethanolic and aqueous solutions is 40.20: 11.19%). These benefits may be related to the presence of CA derivatives such as CAPE and other active constituents in the extract [105]. Propolis inhibited histamine release induced by a compound described as 48/80 and concanavalin A, suggesting the presence of an unknown compound with non-flavonoid and anti-inflammatory activity [23,69]. Propolis showed inhibitory effects on myeloperoxidase activity, ornithine decarboxylase, tyrosine-protein-kinase, NADPH-oxidase, and hyaluronidase from guinea pig mast cells. This anti-inflammatory activity can be explained by the presence of active flavonoids and cinnamic acid derivatives such as acacetin, quercetin, naringenin and CAPE and CA [69,70,71,105,106,107,108,109,110]. Conversely, Ahangari et al. [57] demonstrated that the ethanolic extract of propolis (3, 10, 30, and 100 µg/mL) did not show significant effects on the histamine release induced by ionophore A23187 and ovalbumin. Several authors suggested that only high concentrations of propolis may directly activate mast cells, promoting inflammatory mediator release by cytotoxic mechanisms that could be related to allergic processes in people sensitive to propolis.

It appears that the anti-inflammatory properties of propolis may be based on the following mechanisms: (1) suppression of the release of inflammatory cytokines, such as TNF-α and IL-1β; (2) increase in production of anti-inflammatory cytokines such as IL-4 and IL-10; (3) prevention of TLR4 activation; (4) suppression of LOX, COX-1 and COX-2 gene expression; (5) suppression of NF-κB and AP-1 activities; and (6) reduction in the infiltration of monocytes and neutrophils [105,106,110].

It has been shown that CAPE, strong antioxidants and NF-κB inhibitors alleviate asthma by regulating the airway hyperresponsiveness via the ROS-responsive MAPK/Akt pathway [105]. CAPE treatment reduced ROS levels in the airway microenvironment, attenuated extensive inflammatory cell infiltration and inhibited goblet cell hyperplasia, collagen deposition and fibrosis by significantly limiting secretion of eotaxin-1, TGF-β1, TNF-α, IL-4, IL-13, monocyte chemoattractant protein-1, IL-8, matrix metalloproteinase-9, and alpha-smooth muscle actin expression. As markers of oxidative stress, malondialdehyde (MDA) production and protein carbonyl (PC) levels significantly decreased while GSH level increased after CAPE treatment in an animal allergic asthma model. CAPE (free radical scavenger) prevents the release of oxidant-induced inflammatory mediators, contraction of airway smooth muscles and proliferation of airway smooth muscle cells (ASMCs) by reducing the level of ROS [105,106,107]. CAPE and other flavonoids, including quercetin and kaempferol, alleviate airway inflammation and remodeling in chronic asthma by balancing the airway microenvironment. These effects were shown to be associated with decreased ROS levels because CAPE significantly decreased the phosphorylation of Akt and MAPK pathways (caused by increased ROS).

CAPEs effectiveness was confirmed in a murine model of systemic anaphylaxis induced by ovalbumin (OVA) challenge in mice. After challenging the sham-treated mice, they all developed anaphylactic symptoms, increased plasma levels of histamine and OVA-specific IgE, marked vascular leakage, NF-κB activation, platelet-activating factor (PAF) production, and histological changes including pulmonary edema and hemorrhage in the renal medullae within 20 min. On the contrary, in the CAPE-treated mice, a reduction in the plasma levels of histamine and OVA-specific IgE and inhibition of NF-κB activation and PAF release were observed [97,106]. These results indicate that CAPE is effective in the systemic anaphylaxis model, indicating that CAPE-mediated anti-allergic effects may be the result of its protective effects against IgE-mediated allergy [69,70,71,72,73,74,75,76,100]. CAPE effectively reduced the infiltration of monocytes and neutrophils and inhibited the activation of TLR by interrupting interaction of the ligand (LPS) with the receptor complex (TLR4/MD2) [108,109]. CAPE successfully affects several cellular oxidative processes: (1) inhibits myeloperoxidase activity (MPO) by PMN infiltration; (2) reduces respiratory burst of human PMN; and (3) forms oxide-base in epidermal DNA isolated in mice treated in vivo [109]. Inhibition of LOX and leukotriene production in peritoneal macrophages was also successfully inhibited by CAPE and CA. Their action on LTC4 was less emphasized in vivo [70,71]. Ilhan et al. [110] have shown that CAPE may exert protective effects against experimental allergic encephalomyelitis-induced oxidative stress in rats. Therefore, as a selective inhibitor of NF-κB activation and an inducer of apoptosis, CAPE may provide multiple anti-inflammatory and immunomodulatory effects in numerous chronic diseases associated with oxidative stress and inflammation such as allergic and autoimmune diseases [108].

Isolated antiallergic components in Chinese and Brazilian propolis are kaempferol and chrysin. Chrysin and kaempferol act as antioxidants, whereby they stabilize ROS, preventing tissue damage in allergies. Chrysin and kaempferol inhibit IL-4, prevent the activation of eosinophils, and reduce the total number of eosinophils. Furthermore, kaempferol suppresses CD23 mRNA expression, preventing the second signal for mast cell degranulation to occur; thus, cytokine release decreases, indirectly causing the eosinophil count to decrease. It has been demonstrated that Malaysian and Brazilian propolis are more effective as anti-allergic agents compared to Indonesian propolis.

Other compounds from propolis including quercetin, an active bioflavonoid found abundantly in plants such as primrose leaves, garlic, onion and green tea, showed strong anti-allergic activity. Quercetin has been used clinically in a number of pathological processes, including inhalant allergies, food allergies, AD, chronic sinusitis, bronchitis, asthma, serous otitis media reoccurrences, and bowel inflammatory diseases [101].

Middleton et al. [111] demonstrated that quercetin inhibited ragweed antigen-induced basophil histamine release at concentrations of 5 to 50 μM (80 to 100% inhibition at 50 μM) in a concentration-dependent fashion. Quercetin markedly inhibited antigen-induced, calcium-independent basophil activation in the 1st stage of histamine release and was inhibitory in the antigen-independent, calcium-dependent 2nd stage. Given that quercetin does not affect the ability of histamine release when unstimulated cells are exposed to it but does exert an inhibitory effect during or after antigen activation, it can be concluded that quercetin acts on a basophil process that is activated (“exposed”) as a result of antigen activation. Thus, quercetin acts in a fashion affecting the “opened” calcium channel.

Quercetin is extremely safe in allergic rhinitis therapy. Quercetin has so many other benefits (antioxidant, anti-inflammatory, capillary stabilizer, etc.) It is a strong inhibitor of basophil and mast cell degranulation [70,71,99,112]. In immune reactions, basophils and mast cells that are sensitized by IgE antibodies attached to the cell surface strongly degranulate upon re-exposure to the allergen. Degranulation requires energy and an influx of calcium (Ca^2+^), and results in the simultaneous release of histamine, adenosine triphosphate and other mediators that are stored in the granules. In the process of degranulation, mast cells use calcium-activated enzymes to assemble contractile microtubules, which pulls the granules towards the cell membrane, where the inflammatory contents are spilled outside the cell, unleashing an allergic reaction [69,70,71,99,101,111,112]. Quercetin prevents the process of mast cell degranulation, preventing the entry of Ca^2+^ into the cells Secondary mediators of inflammation, such as arachidonic acid metabolites, are released outside the cell via the action of phospholipase A2. It is known that steroids act as anti-inflammatory agents due to their ability to inhibit phospholipase A2. Quercetin also inhibits many steps along the eicosanoid membrane pathway, including phospholipase A2 and lipoxygenase. Several authors confirmed that quercetin: (1) can inhibit mast cell degranulation; (2) reduces airway hyperreactivity; (3) reduces mucus and collagen production; (4) reduces eosinophil and neutrophil recruitment; (5) reduces bronchial epithelial cell activation and MMP-9 and MMP-12 expression; (6) modulates Th1/Th2 cytokine production; (7) demonstrates anti-fibrotic activities; (8) reduces collagen deposition by inducing HO-1 upregulation; (9) decreases the production of IL-4, IL-5, CCL11 and LTB4 and increase IL-4 and increase IFN-γ concentration; (10) decreases type I and type III collagen synthesis; and (11) downmodulates P-selectin expression by blocking NF-kB signaling. These effects might be associated with attenuation of the PI3-kinase, Akt and NF-kB signaling pathways. Quercetin taken with vitamin C has been reported to reduce hay fever symptoms [99,113]. In fact, it has been shown that flavonoids inhibit enzymes that increase histamine release from mast cells and basophils: cAMP phosphodiesterase and calcium-dependent ATPase. Cyclic AMP phosphodiesterase degrades cAMP; large amounts of cAMP act by blocking intracellular reservoirs of histamine. Moreover, calcium-dependent ATPase degrades ATP to release energy and facilitate the gating of Ca^2^^+^ across the cell membrane; high intracellular Ca^2+^ levels also cause histamine release from cellular storage granules. Quercetin has a strong affinity for mast cells and basophils; stabilizes membranes, prevents release of histamine and has the power to inhibit two enzymes that regulate the release of leukotrienes, which are implicated in asthmatic-type reactions [11,70,101,111,112,113,114]. By inhibiting the release of histamines and leukotrienes into the bloodstream, quercetin prevents allergy symptoms, such as swollen nasal passages, congestion, sneezing, watery eyes, and itchiness in eyes and nose.

Mechanisms of the antiallergic action of flavonoids, such as luteolin, quercetin and baicalein, which are affected both IgE-mediated immune responses, a sensitization phase and an effector phase, are based on their structure and ability to inhibit: (1) the enzymes hexosaminidase (as a key strategy to restrain the degranulation process of mast cells), phospholipase A2 (PLA2) and 5-lipoxygenase (5LO); (2) transport ATPase in histamine secretion from rat mast cells; (3) allergen-stimulated human basophils; (4) cytokine synthesis and secretion IL-4, IL-13 and CD40 ligand (important for the differentiation of B cells into IgE producing cells), granulocyte macrophage-colony stimulating factor, GM-CSF, interleukin (IL)-6 and tumor necrosis factor (TNF)-α and [11,113,114]. Presently, the numerous mechanisms by which flavonoids inhibit synthesis and release histamine in response to cross-linking of the high affinity IgE receptor (FcεRI) are unclear and require further research. According to many researchers [69,112,113,114,115,116], based on their antioxidant and antiallergic properties, flavonoids can inhibit the synthesis and release of many allergic mediators, including Th2-type cytokines (IL-4 and IL-13), and expression of CD40, which is an important ligand in numerous intercellular interactions; these cells (such as mast cells and basophils) express the high-affinity immunoglobulin E (IgE) receptor, leading to an increase in the inflammatory response. It appears that B cells must receive two signals to produce IgE; the first signal originates from the cytokines IL-4 and IL-13 and the second signal occurs when CD40L, which is induced on the surface of T cells after allergen exposure, binds to CD40 on the surface of B cells. This CD40/CD40L interaction is critical for the activation and induction of isotype switching in B cells. T cells that respond after encountering an allergen express CD40L and can then engage CD40 on B cells, monocytes, DCs, and epithelial cells. Polyphenols/flavonoid compounds regulate Th1/Th2 balance and inhibit antigen-specific IgE antibody production by influencing the formation of allergen-IgE complexes and by influencing the binding of this complex to its receptors (FcεRI) on mast cells and basophils. In addition to Th2 cells, tannins isolated from apples can prevent food allergies through increase in γδTCR T cells in intestinal intraepithelial lymphocytes [69,116,117].

The inhibitory effect of several flavonoids on mast cell degranulation was shown to be due to modulation of the receptor-directed Ca^2+^ channels in the plasma membrane [106,113,114,115,116,117] and on β-hexosaminidase as a marker of mast cell degranulation. Thus, apigenin, luteolin, 3.6-dihydroxy flavones, fisetin, kaempferol, quercetin, and myricetin were found to inhibit hexosaminidase release from mast cells with an IC_50_ value of less than 10 μM, while quercetin, quercetagetin, kaempferol-3-O-galactoside, and scutellarin inhibited PLA2 with IC_50_ values ranging from 12.2 to 17.6 μM [116,117]. In addition, luteolin, apigenin and fisetin are the strongest inhibitors of IL-4 and IL-13 synthesis while 3-hydroxyflavone, kaempferol, quercetin, eriodictyol, fustin and 7-hydroxyflavone also inhibit IL-4 production but more weakly. Cirsiliol (3′, 4′, 5-trihydroxy-6,7- dimethoxy flavone) caused 97% inhibition of 5LO activity from rat basophilic cells and 99% suppression of release of cysteinyl leukotrienes from guinea pig lung [69,116]. In particular, quercetin and kaempferol can inhibit IL-4 synthesis with an IC_50_ value of 15.7–18.8 μM. Moreover, Park et al. reported the inhibitory effect of kaempferol on IL-5 bioactivity and histamine release, by basophils and mast cells [116]. IL-4, hydroxylation in positions 7 and 4′ and the presence of OH in either position 3 or 5 is essential for maximal inhibition, while glycosylation of position 3 decreases the activity.

By inhibiting the IL-4-induced signal transduction, flavonoids prevent differentiation of naïve CD4+ T cells into effector T-cells by inhibiting activation of the aryl hydrocarbon receptor (AhR) and NF-κB. AhR is a ligand-activated transcription factor that mediates the toxic and biological effects of many aromatic environmental pollutants such as dioxins [118].

Flavonoids, apigenin, luteolin, baicalein, quercetin, kaempferol and myricetin had noticeable inhibitory effects on AhR activation (with an EC_70_ value equal to 70% of the maximal response to dioxin (2,3,7,8-tetrachlorodibenzo-*p*-dioxin [TCDD]) of 1.9–5.1 μM. On the other hand, marked AhR activation was exhibited by isoflavones such as daidzein, resveratrol (a stilbene), some flavanones such as naringenin, and flavones such as baicalein. The antioxidant activity of flavonoids is mediated by the cytosolic AhR/nuclear factor erythroid 2-related factor 2 (Nrf2) pathway and leads to an increase in antioxidant enzymes such as superoxide dismutase, glutathione peroxidase, catalase, peroxiredoxin, and heme oxygenase-1 [119]. By reducing oxidative stress, propolis and its flavonoids can suppress the nucleotide-binding oligomerization domain, leucine rich repeat containing gene family, and pyrin domain-containing 3 (NLRP3) inflammasome [120,121,122].

Increasing evidence suggests the important role of platelets and their products (e.g., platelet factor 4, β-thromboglobulin, RANTES, thromboxane, or serotonin) in pathogenesis of allergic diseases. A variety of changes in platelet function have been observed in patients with asthma, such as alterations in platelet secretion, expression of surface molecules, aggregation, and adhesion. Moreover, platelets have been found to actively contribute to most of the characteristic features of asthma, including bronchial hyperresponsiveness, bronchoconstriction, airway inflammation, and airway remodeling. PAF is a phospholipid mediator formed by different cells including eosinophils, macrophages, platelets, neutrophils and vascular endothelial cells. Most of the PAF actions can be prevented by blocking its receptor with antagonists. According to Ling et al. [123], flavonoids (as PAF antagonists) exhibited >50% inhibition on the binding activity at 18.2 µg/mL. Among the flavonoids that inhibit PAF with IC_50_ values ranging from 19.5 to 62.1 µM, the following compounds were the most important: quercetin 4′-methyl ether, quercetin 7,4′-methyl ether, kaempferol 420-methyl ether, kaempferol 3-O-rutinoside, taxifolin 4′-methyl ether, taxifolin 7-methyl ether and eriodyctiol 7,4′-dimethyl ether naringenin 4′-methyl ether. Flavonoid compounds exert protective effects against different types of inflammatory stimuli, such as ultraviolet radiation, egg white, rat paw edema, and others [70,101]. However, the potential of flavonoids as therapeutic agents in allergies needs to be reexamined. The flavonoid components (apigenin, luteolin, flavone, quercetin, kaemperol, rhamnetin, fisetin, naringenin, dihydrofisetin, dihydroquercetin and (+)-catechin) markedly inhibited the released histamine, and IgE production from cells stimulated with the calcium ionophore, A23187. Similarly, a diet rich in the antioxidant vitamin E appeared to protect people against hay fever, while a high intake of beta-carotene or oleic acid, and fatty acids in olive oil, which also is an antioxidant, seemed to increase the risk of skin allergic reactions [114,121,124].

Important mechanisms of polyphenols in antiallergic responses may be the following: (1) inhibition of histamine and β-hexosaminidase release; (2) reduction in proinflammatory cytokines, and leukocyte production; (3) regulation of Th1/Th2 balance; (4) inhibition of cytokine release; (5) inhibition of allergen-specific IgE antibody complex formation; (6) inhibition of binding allergen-IgE complex to its receptors (FceRI) on mast cells and basophils; (7) suppression of eosinophil production; reduction in the number of inflammatory cells and their accumulation (eosinophils, lymphocytes, neutrophils, monocytes and basophils); (8) inhibition of mast cell degranulation; (9) activation of Nrf2/HO-1 signaling; (10) inhibition of Lyn/Syk, NF-κB and p38 MAPK signaling pathways in activated mast cells; (11) reduction in cell hyperplasia and mucus accumulation in bronchioles; (12) certain polyphenols can increase Treg cells, including CD8 + CD25 + T cells, TCRγδ + T cells, and natural killer (NK)1.1 + T cells.

#### 5.2.2. Anti-Inflammatory Pathways of Propolis and Related Flavonoids

It is known that specific-immune events, such as hypersensitivity reactions (type I, II, III, IV), can lead to inflammation [54,55,56,75,76]. Inflammations are known to be complex processes, caused by ROS, local disturbance of blood circulation and abnormal tissue metabolism. Collectively, these abnormal processes lead to tissue damage, edema formation and activation of membrane-bound phospholipase A2. The latter step often marks the beginning of control mechanisms decisive in stimulation of activities or initiation of disease symptoms [54,55,56,70,71]. As a consequence, cyclooxygenases (COXs) and lipoxygenases (LOXs) are able to transfer oxygen to liberate arachidonic acid, leading to formation of a vast amount of reactive species (such as epoxides, endoperoxides or hydroperoxides) and inflammatory mediators. Cyclooxygenase-dependent processes influence inflammation, pain, fever, blood pressure and platelet aggregation by releasing prostaglandins, prostacyclins and thromboxane. The main sources of these cyclooxygenase-derived mediators, during acute inflammation, are endothelial cells and mononuclear phagocytes [54,55,56,70,71]. On the other hand, the lipoxygenase-dependent pathway leads to the formation of lipoxines and leukotrienes via oxidation of arachidonic acid. Both classes of mediators are known to act in a strong chemotactic way for phagocytic cells, especially neutrophils. Some of them also work in a spasmogenic way or activate respiratory burst and degranulation of leukocytes [54,55,56,69,70,71,72]. Thus, lipoxygenase plays a decisive role in pathological processes, such as inflammation or allergies [54,55,56,70,71,125,126]. Moreover, certain enzymatic products of lipoxygenases are thought to be essential for tumorigenesis [65]. The attracted neutrophils at the site of inflammation produce superoxide via respiratory burst by their NADPH oxidase. As a consequence, hydrogen peroxide, hydroxyl radicals and hypochlorite and related derivatives are formed by myeloperoxidase activity. Overall, the previous processes can feed the affected tissue with reactive species, which leads to oxidative stress that disturbs the pro- and anti-oxidant balance.

The anti-inflammatory effects of propolis have been confirmed by numerous studies in the literature [70,71,126,127,128,129,130,131,132,133]. Several studies have shown that propolis (as an aqueous or ethanol preparation) can act as a strong anti-inflammatory agent against acute and chronic inflammations [70,71,126,127,128,129,130,131,132,133]. The efficacy of aqueous extract of propolis (WSDP) was confirmed in an acute model of inflammation (paw edema induced by PGE2) and a chronic model of inflammation (arthritis induced by formaldehyde) [130]. The anti-inflammatory effects of WSDP are based on inhibition of platelet aggregation, in vitro PG biosynthesis, and adjuvant-induced paw edema in vivo, when administered orally, and the effect is dose-dependent [128,129,130,131]. However, WSDP has no effect against carrageenan-induced forms of edema and Freund’s adjuvant-induced arthritis [128,129,130]. The anti-inflammatory effects of orally administered ethanolic extract of propolis (EEP), rich in phenolic acids and flavonoids, are evident in the inhibition of various models of inflammation, such as Freund’s adjuvant-induced arthritis, cotton-pellet-induced granuloma, carrageenan-induced edema and the effect on vascular permeability and analgesia in a dose-dependent manner [128,129,130]. Tanaka et al. and Parisi et al. [130,131] demonstrated a reduction in serum levels of IL-17, IL-1β, and IL-17, triggering cytokine production by propolis in mice with collagen-induced arthritis. EEP and selected flavone derivatives (chrysin, galangin, kaempferol and quercetin) exerts its inhibitory effect on the IL-1beta and iNOS gene expression in J774A.1 macrophages at the transcriptional level. Published studies [131,132] confirmed the efficacy of propolis in a randomized clinical trial in patients with rheumatoid arthritis; propolis had a positive effect on inflammatory biomarkers, oxidative stress status, and clinical symptoms of RA patients. However, Matsumoto et al. [133] showed that Brazilian propolis had no effects on disease activity in patients with RA. The inflammatory response may also be implicated in a myriad of diseases including dermatological conditions, such as psoriasis, arthritis (rheumatoid/osteoarthritis), and a number of other pathologies [70,71,98]. Ethanolic extract of Coation propolis had a relevant topical anti-inflammatory effect in a model of cutaneous inflammation in mice; EEP reduced the total number of inflammatory cells and macrophage spreading index in the peritoneal cavity and inhibited the cellular migration of polymorphonuclear leukocytes in skin, an important step in the inflammatory process [70,71]. Antioxidant compounds, including propolis and its flavonoids (quercetin, chrysin, curcumin), protect against psoriatic complications through their roles as inhibitors of inflammation and free radical scavengers. EEP-treated skin samples exhibited slight hyperkeratosis, absence of parakeratosis, mild acanthosis, absence of elongated rete ridges, and mild dermal inflammation. In addition, flavonoids present in propolis can inhibit the degranulation of mast cells basophils, reduce histamine, tryptase, IL-4, IL-5 IL-6, IL-8, IL13 and other inflammatory mediators released from cultured mast cells and macrophages [18,69,70,71,100,109,110,111,112,113]. Several flavonoids were found to inhibit the release of histamine, leukotrienes, prostaglandin D2, and granulocyte macrophage-colony stimulating factor (GM-CSF) from mast cells in a concentration-dependent manner [70,97]. The inhibitory effects of certain flavonoids on mast cell degranulation are attributed to the modulation of the receptor-directed Ca^2+^ channels in the plasma membrane and/or calmodulin. In addition, the use of propolis suppressed cytokines produced by monocytes/macrophages (IL-1β and IL-12) and Th1 type (IL-2) lymphocytes, whereas the production of TGF-β1 by T regulatory cells increased.

In acute peritonitis induced by zymosan in vivo, propolis had the ability to suppress generation of LOX and COX in murine peritoneal macrophages and reduced high production of LTB4 and LTC4 [134]. However, its oral supplements do not affect PGE2 generation in zymosan-treated mice ex vivo or in vitro [120,128,134]. In other mouse models with uric acid crystal-induced peritonitis, the alcoholic extract of Taiwanese green propolis (TGP) attenuated the peritoneal recruitment of neutrophils, and the levels of IL-1β, active caspase-1, IL-6 and monocyte chemoattractant protein-1 in lavage fluids. Propolin G component was separated from TGP and identified as a potential inhibitor of the NLRP3 inflammasome [18]. The authors suggested that TGP could be useful in ameliorating gouty inflammation via the inhibition of the NLRP3 inflammasome. Active components of propolis with anti-inflammatory properties are galangin, quercetin, chrysin, CAPE, and artepillin C in Brazilian propolis, which reduce cytokine and chemokine production, and inhibit T cell activation and proliferation through NF-kB inhibition. For example, CAPE induces immunosuppressive activity, inhibiting the early and late events of T cell activation and the consequent release of cytokines such as IL-2. Inhibition of NF-κB results in reduced expression of COX-2 and in potent NO inhibition by blocking the activation of iNOS [133]. Propolis has inhibitory effects on prooxidative enzymes, such as myeloperoxidase activity, NADPH-oxidase [71,126], ornithine decarboxylase, tyrosine-protein-kinase, and hyaluronidase from guinea pig mast cells [126] reducing the level of ROS production and accumulation of inflammatory cells. This anti-inflammatory activity can be explained by the presence of active flavonoids and cinnamic acid derivatives [119,120,135,136,137,138]. The former include acacetin, quercetin, and naringenin (terpenoid constituents may exert an addictive effect; the latter include CAPE and CA [11,12,13,14,15,16,17,18,19,20,21,22,23,24,25,26,27,28,29,30,31,32,33,34,35,36,37,38,39,40,41,42,43,44,45,46,47,48,49,50,51,52,53,54,55,56,57,58,59,60,61,62,63,64,65,66,67,68,69,70,71,72,73,74,75,76,77,78,79,80,81,82,83,84,85,86,87,88,89,90,91,92,93,94,95,96,97,98,99,100,101,102,103,104,105,106,107,108,109,120,126,135,136,137,138]. Furthermore, it was reported that CAPE has anti-inflammatory activity, inhibiting the release of arachidonic acid from cell membranes, suppressing the enzyme activities of COX-1 and COX-2, and inhibiting the activation of COX-2 gene expression [105,106,107,108,109,111,122]. The anti-inflammatory activity of propolis has largely been attributed to CAPE, a phenolic antioxidant which causes a nonselective inhibition of COX-1/COX-2 and suppression of 12,O-tetradecanoylphorbol-13-acetate (TPA)-induced COX-2 mRNA expression (101, 108–111). CAPE also inhibited H_2_O_2_ formation by activated polymorphonuclear neutrophils that infiltrate the skin following TPA application [139].

Propolis and its components such as CAPE, CA, quercetin, and naringenin, inhibit the production of eicosanoids. In fact, these components significantly suppressed the lipoxygenase pathway of arachidonic acid metabolism, and CAPE was the most potent modulator. This CAPE activity can be attributed to the reduction in c-Jun-N-terminal kinase (JNK1/2) and NF-κB activation and the decrease in COX-2 expression [122,139].

It was also shown that nobiletin, amentoflavone, quercetin, quercetin pentaacetate, flavone, resveratrol, apigenin, chrysin, kaempferol, galangin and genistein may be non-selective inhibitors of COX-1 and COX-2 [140,141]. Pecyna et al. [141] demonstrated that hydroxylated but not methoxylated resveratrol analogues were able to bind to the enzymes. Therefore, hydroxylated resveratrol analogues represent a novel class of highly selective COX-2 inhibitors and promising candidates for in vivo studies. Furthermore, promising in vitro data indicated that treatment with selective COX-2 inhibitors such as curcumin, chlorogenic acid, CA, resveratrol, galangin or the flavonoid silymarin may reduce the risk of Alzheimer’s, and Parkinson’s disease [142,143] and also be effective in the treatment of asthma and other allergic diseases [125,136,144,145].

**Figure 4:**
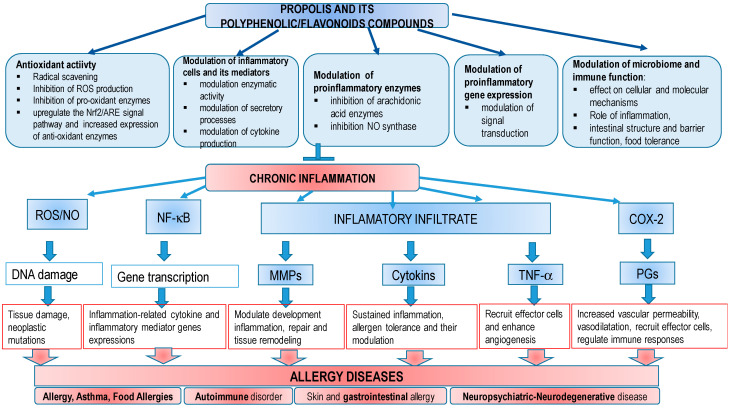
Inflammatory pathways as potential targets for propolis and related flavonoids in allergy treatment. Propolis and its components, with their antioxidant and anti-inflammatory abilities, modulate the activity of inflammatory cells, enzymes and pro-inflammatory genes as well as gut microbiome and immune function. By blocking the accumulation of inflammatory cells, such as phagocytic cells, mast cells and endothelial cells, they reduce the production of important inflammatory mediators including proteins, peptides, glycoproteins, cytokines, arachidonic acid metabolites (prostaglandins and leukotrienes), nitric oxide, and oxygen free radicals. ROS/NO produced by inflammatory and endothelial cells can lead to tissue damage as well as activation of NF-κB, which regulates many aspects of innate and adaptive immune functions and serves as a key mediator of inflammatory reactions. Note: ROS, Reactive oxygen species; NO, Nitric oxide; NF-κB, Nuclear factor kappa-light-chain-enhancer of activated B cells; COX-2, Cyclo-oxygenase-2; MMPs, Matrix metalloproteinases; TNF-α, Tumor necrosis factor alpha; PGs, Prostaglandins responses.

The anti-inflammatory activities of flavanols (quercetin, rutin and morin) and flavanones (hesperetin and hesperidin) were investigated in animal models of acute and chronic inflammation. There are numerous mechanisms (Figure 4) that explain the anti-inflammatory ability of propolis and its flavonoids, including: (1) strong antioxidant and radical scavenging activity, (2) regulation of the activity of inflammatory cells, (3) inhibition of arachidonic acid metabolism enzymes (phospholipase A2, COX, LOX) and nitric oxide synthase, (4) modulation of the production of proinflammatory cytokines and mediators, and (5) reduction in the expression of pro-inflammatory genes [113,114,115,116,117,118,140,141,142,143,144,145]. It should be emphasized that the main processes of flavonoids resulting in anti-inflammatory effects are based on: (1) inhibition of the proinflammatory enzymes (COX, LOX, and inducible NO synthase; (2) inhibition of transcription factors NF-κB and activating protein-1 (AP-1); (3) activation of phase II detoxification enzymes through the anti-oxidant responsive element, including glutathione reductase, glutathione peroxidase, heme oxygenases, γ-glutamyl cysteine synthetase, superoxide dismutase and catalase; and (4) modulation of signaling pathways, such as protein kinase C, mitogen-activated protein kinase (MAPK), and nuclear factor-erythroid 2-related factor (Nrf2). Nrf2 is the transcription factor responsible for both constitutive and inducible expression of the antioxidant responsive element-regulated genes, whose protein products are involved in the detoxication and elimination of reactive oxidants and electrophilic agents through conjugative reactions and by enhancing cellular antioxidant capacity. Its activity results in the suppression of MCP-1 and vascular cell adhesion molecule-1 expression, monocyte adhesion to endothelial cells and transmigration, and activation of p38 MAPK.

The anti-inflammatory mechanism of propolis and flavonoids such as quercetin, luteolin, anthocyanin, hyperin and alpinetin on the TLR4/NF-κB/NLRP3 signaling pathway has been confirmed in publications [146,147,148]; it is based on the interruption of different signaling phases of the NLRP3 inflammation pathway in vitro and in vivo, by reducing the expression of NLRP3 inflammasome-related components, such as IL-1β, IL-18, NLRP3 and caspase-1 and/or blocking the inflammasome assembly, such as ASC oligomerization via signaling molecules (e.g., TLR4/NF-κB/NLRP3, PPARγ, TXNIP and Syk/Pyk2, and others). For example, EGCG reduces peritoneal inflammation by inhibiting NLRP3 expression and IL-1β release in mice treated with monosodium urate (MSU) crystals [148] through inhibition of the NLRP3 inflammasome conjugation with thioredoxin-interacting protein (TXNIP) in THP-1 cells, whereas quercetin inhibits NLRP3 and IL-1β expression and caspase-1 activity in human colonic epithelial cells.

The anti-inflammatory mechanism of resveratrol has been shown to be based on its ability to remove ROS, inhibit COX and activate many anti-inflammatory pathways, including Sirtuin-1 (Sirt1), which interferes with TLR4/NF-κB/STAT signal leading to reduced production of cytokines and pro-inflammatory factors from inactivated immune cells, macrophages and mast cells. Thus, the addition of resveratrol to the human diet can be promising in treating immune disorders, but only in the form of nanoparticles due to rapid metabolism in the body [149].

Regardless of the low bioavailability of flavonoids in the intestine, and consequently, the low active concentration in plasma and target tissues, it seems that even a low concentration of flavonoids is sufficient for the activation of the Keap1/Nrf2/ARE pathway and inhibition of the NF-κB pathway as the main mechanisms of health benefits [150]. The key mechanisms of the anti-inflammatory effects of the flavonoids in the gut are the following: (1) strong antioxidant and/or antiradical properties, (2) the effect of nitric oxide (NO) on the metabolism, (3) inhibition of proinflammatory cytokine production, (4) inhibition of lipoxygenase and reduction in leukotriene B4 (LTB4) production, (5) preservation of colonic absorptive function, and (6) effects on TLR and inflammasome pathways. Accordingly, it is likely that propolis and its flavonoids represent suitable compounds that may limit the development of allergic/inflammatory reactions or even prevent them.

#### 5.2.3. Modulation of Cytokine/Chemokine Production by Propolis and Flavonoids

Cytokines, key modulators of inflammation, are redundant and pleiotropic; various cytokines can target the same receptor, and a single cytokine can have multiple, even contradictory effects. Cytokines have a specific effect on interactions and communication between cells and regulate the intensity and duration of inflammatory responses.

It was shown that propolis and flavonoids inhibit IL-10, IL-4 and IL-13. IL-4 and IL-10 inhibit IL-12-induced IFN-γ secretion. Moreover, IL-10 antagonizes many proinflammatory effects of TNF-α and IL-1β whereas the production of IL-4 and IL-10 by monocytes can be downregulated by IFN-γ.

Flavonoids are able to decrease the expression of different proinflammatory cytokines/chemokines, including TNF-α, IL-1β, IL-6, IL-8 and monocyte-chemoattractant protein-1, in different cell types such as RAW macrophages, Jurkat T-cells and peripheral blood mononuclear cells. Recently, it was reported that CAPE exhibits inhibitory effects on the production of proinflammatory cytokines IL-1β, TNF-α, and monocyte chemoattractant protein 1 (MCP-1) from lipopolysaccharide- (LPS-) stimulated RAW264.7 macrophages [151]. Macrophages are ubiquitous cells that secrete a number of potent bioactive inflammatory mediators such as growth factors, cytokines, proteolytic enzymes, proteoglycans, lipid mediators and prostaglandins [3,22,25]. They play a critical role in all phases of the inflammatory process, including initiation, maintenance, and resolution. Furthermore, macrophages are important cells in the primary response to pathogens, maintenance of tissue homeostasis, inflammation, and immunity [3,25]. Macrophages, along with other inflammatory cell types, provide a wide range of bioactive molecules that cause marked changes in the inflammatory loci by interacting with epithelial, mesenchymal and vascular endothelial cells. For example, genistein quercetin, luteolin and luteolin-7-glucoside were found to prevent IL-6, IL-1β, and TNF-*α* formation in LPS-induced human monocytes, as in macrophages, gastric epithelial cells, and osteoblasts while eriodictyol and hesperetin only inhibited TNF-α release [69,114,151,152,153,154,155,156,157].

Alongside inhibiting cytokines, propolis and flavonoids may act on chemokines modulation [99,126,157] including: (1) CC or β chemokines, (MCP-1, MIP-1*α*, RANTES, and eotaxin) and (2) CXC or α chemokines (IL-8, GRO-α, and epithelial-derived neutrophil-activating peptide (NEA-78)). CC chemokines act primarily on lymphocytes, monocytes, basophils, and eosinophils, but not on neutrophils while chemoattractant cytokine IL-8 and the other CXC chemokines act primarily on neutrophils. IL-8 is produced by a variety of tissue and blood cells and its application induces local exudation and a massive, long-lasting accumulation of neutrophils, which release enzymatic granules and a large amount of ROS after stimulation. Since flavonoids exhibit antioxidant and anti-inflammatory effects, they are subjects of interest for pharmacological modulation of ROS production. Thus, in a human whole blood assay, flavonoids presenting a catechol group in the B ring were good inhibitors of IL-1β, IL-6, TNF-α, INF-γ, and IL-8 production, but inhibited IL-8 to a smaller extent. It is known that tumor necrosis factor alpha (TNF-α) is present in the synovial fluid of patients with rheumatoid arthritis and induces the expression of proinflammatory cytokines in synovial cells. Given that reactive oxygen intermediates play an important role in mediating TNF-α action, Shaik et al. [99] and Jafarinia et al. [114] examined the effect of quercetin on TNF-α induced expression of IL-8 and monocyte chemoattractant protein -1 (MCP-1) in cultured human synovial cells. The authors demonstrated that quercetin inhibited TNF-α mediated induction of IL-8 and MCP-1 expression, by inhibiting activation NF-κB [158]. Shaik et al. [99] suggested that quercetin is unique in its ability to inhibit TNF-α transcription by inhibiting phosphorylation and activation of JNK/SAPK and therefore, suppressing protein AP-1-DNA binding. However, quercetin and Ginko biloba extract (EGb 761) inhibited ERK 1/2 phosphorylation and p38 mitogen-activated protein kinases (MAPK) activity, which are important in post-transcriptional regulation of TNF-α mRNA. Thus, dietary flavonoids have an important role in the regulation of redox inflammatory networks from NF-kB to MAPKs through the perturbation of redox-sensible networks in immune cells.

NF-ĸB is one of the most important transcription factors, which regulates the transcription of more than 400 genes, including the expression of inflammatory response genes; for example, cytokines, such as IL-1β, IL-6, TNF-α and IL-8 as well as COX-2. Inactivation of NF-kB by flavonoids (daidzein, genistein, isorhamnetin, kaempferol, quercetin, naringenin, and pelargonidin) reduces inflammatory cytokines, ROS production, and LPS activation of NF-kB. Flavonoids prevent the transcription of factors, such as AP-1 or NF-ĸB by modulating the PKCs and MAPKs signaling pathways. The main mechanism of flavonoids (genistein, quercetin, galangin) to reduce the inflammatory response is via inhibition of NF-ĸB activation. In addition, daidzein, genistein, isorhamnetin, kaempferol, quercetin, naringenin and pelargonidin were described as inhibitors of LPS activation of NF-ĸB. Moreover, kaempferol inhibited phosphorylation of ERK-1/2, p38, and JNK, and activation of NF-ĸB by IL-1*β* in rheumatoid arthritis fibroblasts. In this same study, kaempferol inhibited the production of COX-2, PGE2, and MMP-1 and MMP-3 [156,157,158,159].

Moreover, flavonoids inhibited: (1) growth factors such as EGF, fibroblast growth factor, insulin-like growth factor 1, keratinocyte growth factor, hepatocyte growth factor, PDGF; and TGF-β that globally contribute to cellular proliferation and amplification of the inflammatory response; (2) PAF; (3) kinin; (4) inflammasome; (5) C-reactive protein; (6) serum amyloid A (7) vasoactive amines; and (8) proteases.

It should be noted that several cytokines and growth factors use ROS as second messengers, and ROS can be implicated in many signaling pathways and processes, such as cell growth or inhibition, mitogenesis, differentiation, apoptosis of several cell types, vascular injury, angiogenesis, immunosuppression, cell respiratory burst depression, Treg and immature myeloid cells development, and others [160].

#### 5.2.4. The Antioxidant Pathway of Propolis and Flavonoids in the Regulation of Allergic Diseases

Flavonoids have powerful antioxidant properties in vitro and in vivo and are able to scavenge a wide range of reactive oxygen, nitrogen, and chlorine species, such as superoxide, hydroxyl radical, peroxyl radicals, hypochlorous acid, and peroxynitrous acid (Table 9). Flavonoids can also chelate metal ions, often resulting in decreased prooxidant activity of metal ions [55,56,65,70,71,96,101,120,136].

First, flavonoids as effective scavengers of free radicals may decrease levels of oxidative DNA damage in vivo. Phenols might exert direct antioxidative effects within the gastrointestinal tract; these effects could include binding of prooxidant iron, scavenging of reactive nitrogen, chlorine, and oxygen species, and perhaps inhibition of COX-2 and LOXs.

As a “bioreactor”, the GI tract is constantly exposed to reactive oxygen, chlorine, and nitrogen species; many of them originate from the diet, and others originate from activation of phagocytes in the gut [161]. The sources of high levels of oxidative stress in the GI tract are attributed to: (1) mixtures of ascorbate and Fe^2+^ in the stomach during iron uptake as a powerful pro-oxidant combination; (2) heme proteins in the diet, as potential strong pro-oxidants; (3) dietary lipid peroxides, cytotoxic aldehydes and isoprostanes that can be increased by gastric juice; (4) nitrite in saliva and foods converted to HNO_2_ by gastric acid, forming nitrosating agents and DNA-deamination compounds; (5) high concentrations of H_2_O_2_ in certain beverages; (6) the presence of highly oxidizing, pro-oxidative phenolic compounds, such as hydroxy hydroquinone in the GI tract; and (7) diet-derived bacteria and toxins that activate the immune system cells present in the GI tract. The antioxidant capacity of flavonoids and other phenolic compounds directly protects the GI system by removing reactive oxygen and chlorine species, inhibiting heme protein-induced peroxidation, DNA base deamination by HNO_2_-derived reactive nitrogen compounds, as well as by chelation of metals, thereby reducing their pro-oxidative activity and possible intestinal damage [161,162]. In addition, phenolic compounds can enhance toxin-metabolizing enzymes or antioxidant defense enzymes in the GI tract [163]. Iron from food is usually not fully absorbed and enters the feces where it can present a pro-oxidative challenge for the colon and rectum [164], especially when the diet is high in fat and low in fiber, which enhances its pro-oxidative activity. Flavonoids, ascorbates and vitamin E reduce fecal mutagenicity by maintaining genomic stability [161,162,164,165].

Briefly, the antioxidant action of flavonoids occurs by inhibiting the activity of several pro-oxidative enzymes (xanthine oxidase, COX, protein kinase C, ascorbic acid oxidase, LOX, cAMP phosphodiesterase and Na+/K+ ATPase) and the consequent inhibition of ROS production, radical scavenging, inhibition of lipid peroxidation by chelating metal ions, mainly iron and copper, or by potentiating the synthesis of antioxidant molecules through upregulation of the signaling pathway of nuclear factor E2-related factor 2/antioxidant response element (Nrf2-ARE). Good scavenging activity requires the presence of a catechol moiety in the B- ring along with a 3-hydroxy group in combination with a 2–3 carbon double bond in the C- ring of flavonoids, resulting in increased Fe^2+^ chelation and inhibition of the of lipid peroxidation rate.

In addition, the enhancement of endogenous antioxidant defense by propolis and its flavonoids was linked to the direct elimination of ROS, inhibition of lipid peroxidation and GSH oxidation; increase in reduced GSH levels; and restoration of activities of antioxidant enzymes, such as superoxide dismutase, catalase, glutathione S-transferase and glucose 6-phosphate dehydrogenase; and activation of Erk-Nrf2-HO1, GCLM, and TrxR1 signal pathways [166,167]. Therefore, the binding of flavonoids to AhR can result in activation of Nrf2 and production of molecules that protect cells from oxidative damage.

The antioxidative effect of flavonoids in biological system is related to: (1) their ability to scavange ROS including singlet oxigen (^1^O_2_), hydroxyl radicals (OH^●^), hydrogen peroxide (H_2_O_2_), superoxide anions (O_2_^−●^), perhydroxy radicals (HO_2_^●^), lipid radicals (LO^●^) and lipid peroxy radicals (LOO^●^; (2) their ability to scavange nitric reactive radicals (HOONO, NO, NO_3_, and others); (3) inhibition of oxidative enzymes; (4) metal ion chelation (Cu^2+^, Fe^2+^, Zn^2+^, Mg^2+^); (5) increase in the activity of antioxidant enzymes and their protection [66,67,127,146].

The antioxidant properties of propolis and flavonoids and their ability to increase the activity of antioxidant enzymes is essential in reducing ROS and oxidative stress and consequently reducing bronchoconstriction and airway hyperreactivity [66,67,120,136,166,167,168], and establishing tissue homeostasis via increased activities of superoxide dismutase (SOD), catalase (CAT), glutathione peroxidase (GPx), glutathione reductase (GR), and GSH [168]. The ability of flavonoids to capture free radicals with flavonoids enables tissue repair, which shows the importance of several different cooperative and synergistic mechanisms of propolis and its polyphenolic compounds in protecting the organism from the damage caused by ROS.

A low intake of antioxidants vitamins C and E, selenium and flavonoids in food can disturb the oxidant/antioxidant balance in patients with asthma, which can be further impaired by the consequent decrease in the concentration of these and other antioxidants in the body, such as bilirubin and albumin in the plasma and airway epithelial lining fluid. However, vitamin C and E supplementation in patients with mild asthma did not show significant benefits over standard therapy.

In children with allergic asthma and respiratory diseases, a lack of reduced glutathione in whole blood and some disorders in the phagocytic and oxidative burst activity of monocytes were observed. Chernyshov et al. [169] showed that the use of reduced glutathione, L-cysteine and anthocyanin led to an increase in the production of IFN-γ, lymphocyte response to mitogens, NK cell activity, increase in the percentage of naive CD4(+) T lymphocytes, which resulted in a significant improvement in the clinical status of the patient.

According to references [170,171], GSH levels in antigen-presenting cells may play an integral role in cellular immunity and disease progression. For example, GSH levels and antigen-presenting cells determine which response will be dominant (Th1 or Th2) [170]. The Th1 response is characterized by production of IL-12 and IFN-α and enhancement of delayed hypersensitivity response; the Th2 response is determined by IL4 and IL-10 production and upregulation of a number of antibody responses.

GSH is a ubiquitous intracellular thiol present in all tissues, including the lungs. Its redox system consists of primary and secondary antioxidants such as glutathione peroxidase (GPx), glutathione reductase (GR), glutathione S-transferase (GST) and glucose 6-phosphate dehydrogenase (G6PD). GSH is a key intracellular antioxidant that maintains cellular integrity by creating a low oxidation state environment via its multiple functions, including detoxification of xenobiotics, synthesis of proteins, nucleic acids, and leukotrienes. GSH provides lung protection against oxidative injury caused by various endogenous or exogenous lung toxicants and can be found in high concentrations in the bronchoalveolar lavage fluid (BALF), while GSH deficiency leads to an increased risk of lung damage and disease. Considering its role, GSH is an important marker of many lung diseases [171].

Quercetin acts as an antioxidant by inhibiting xanthine oxidase, lipoxygenase and NADPH oxidase, which are oxidative enzymes that play key roles in the initial process of free radical-induced cellular damage [172,173,174]. In addition, quercetin may act on signaling pathways and its indirect interaction with the endogenous antioxidant defense system [172,173,174], such as phase I and II detoxifying enzymes. Quercetin has ability to increase the expression of the rate limiting enzyme c-glutamyl cysteine synthetase in the synthesis of GSH and induce xenobiotic responsive elements in CYP 450 family genes [172,173,174]. Furthermore, plant-derived flavonoids or polyphenols, including quercetin, may act as non-stressful and non-cytotoxic heme oxigenase-1 (HO-1) inducers, maximizing the cellular intrinsic antioxidant potential [174]. Morales et al. [175] suggested that renal protection exerted by quercetin is based on its ability to increase metallothionein in the kidneys. Rutin and chlorogenic acid effectively scavenge ROS and inhibit histamine release by upregulating multiple cytokines, including IL-10, IL-13, IFN-γ, IL-6 and TNF-α in antigen-IgE activated mast cells. EGCG inhibits superoxide-induced histamine release from mast cells, showing that they act as a cell membrane stabilizer and a radical scavenger. In addition, dietary intake of flavonoids may affect APC cell function by fine-tuning levels of intracellular ROS and thus modulate APC activation or deactivation and play a key role in immune defense as well as protection against overt immune-mediated inflammation under chronic antigenic stimulations. Accordingly, propolis and flavonoids may be effective in many allergic diseases and immunologic disorders, such as gastrointestinal diseases, respiratory diseases (asthma, rhinitis, COPD) and inflammatory skin diseases such as AD, urticaria, psoriasis, and other skin diseases. The anti-inflammatory activity of flavonoids in the skin is related to the same mechanisms found in the GI system, including: (1) antioxidant and/or antiradical ability; (2) inhibition of NO production; (3) inhibition of LOX and reduction in leukotriene B4 (LTB4) production; (4) reduction in proinflammatory cytokine production; (5) protection of the skin from pathogenic microbes; and (6) increased GSH concentration, catalase and superoxidase activity [69,70,71]. Furthermore, flavonoids accelerate the healing process of damaged skin by increasing GSH levels. Flavonoid advantages compared to other drugs are as follows: (1) acceleration of skin re-epithelialization and healing; (2) inhibition the production of SASP components (TNF-α, IL-1β, IL-6, and IL-10); (3) strong antioxidant, anti-inflammatory, and antimicrobial activities, and anti-cancerogenic, and vasodilating properties; (4) low cost and availability; and (5) reduction in skin aging and loss of elasticity. Given that patients with allergic diseases have a very poor quality of life, propolis and flavonoids may alleviate and reduce the numerous consequences of allergic diseases and improve their quality of life.

#### 5.2.5. Effect of Propolis and Flavonoids on Mucociliary Clearance

As previously noted, ROS attack proteins to form carbonyls and can react with nitrogen species and tyrosine to form nitrotyrosine while their reaction with DNA can form base pair derivatives, such as 8-oxo-2-deoxyguanosine. Oxidants also destroy the ciliary functions of the respiratory epithelium and decrease the surfactant activity while increasing mucus secretion, activity of cytokines and proteases, neutrophil chemotaxis and alveolar permeability and smooth muscle contractility [176,177].

Lung defense involves cough, anatomical barriers, aerodynamic changes, and immune mechanisms; however, the primary defense mechanism is mucociliary clearance (MCC). In asthma, MCC is a non-specific defense mechanism of the respiratory tract. Ciliated cells, which line the surface epithelium of the airways, provide the necessary force for MCC via coordinated beating of their cilia. MCC damage is a feature of chronic respiratory diseases, including asthma. In vitro and in vivo studies indicate that inflammatory mediators affect the mucociliary apparatus. The role of MCC is to eliminate inhaled pathogens and irritants from the surface of the respiratory epithelium. Epithelial dysfunction (loss of physiological and biological barrier) and increased penetration of inhaled pathogens and toxins affects the inflammatory response, increases secretion of mucus, reduces the mobility of ciliary villi, and reduces mucociliary clearance, resulting in difficulty in breathing; these are several characteristics of bronchial asthma. Although the mechanisms of MCC damage in asthma are not clearly understood, airway inflammation is thought to play a major role [176]. Asthma is generally initiated by allergen-induced activation of DCs that stimulate the development and proliferation of T-helper cells: Th1, Th2, Th9, and Th17. These T helper cells subsequently promote the expression of inflammatory cytokines and chemokines. Subsequently, inflammatory cells, such as neutrophils and eosinophils, and the epithelial-mesenchymal transition of airway smooth muscle cells accumulate. Chemoattractants such as eotoxin are an important factor for eosinophil recruitment to the asthmatic airway, which leads to eosinophilic airway inflammation due to CCR receptors on eosinophils [178]. Furthermore, the elevation of eotoxin levels was proportional to eosinophil infiltration and bronchial hyperreactivity [178]. Nobiletin significantly decreased the levels of eotoxin in serum and bronchoalveolar lavage fluid (BALF) of asthmatic rats and increased the apoptosis index of eosinophils by Fas ligand/Fas receptor molecular interactions. Genistein inhibited IL-5–induced protein tyrosine phosphorylation and gene expression in eosinophils [179] while the molecular mechanisms of EGCG appear to include pleiotropic effects in a murine model of asthma after toluene diisocyanate inhalation (TDI). For example, EGCG successfully reduced the high number of neutrophils, eosinophils, lymphocytes, macrophages, and the total cells, MMP-2, MMP-9 production, hyper-responsiveness (AhR), mucus overproduction [180], overexpression of Th2-mediated cytokines including IL-4, IL-5, IL-13 and TNF-α, and chemokines such as eotoxin and RANTES and ROS generation. It was reported that EGCG suppresses the LPS-induced phenotypic and functional maturation of murine DCs through the inhibition of mitogen-activated protein kinases and NF-κB [180]. EGCG treatment alleviated tissue injury, inflammation, mucus production and collagen deposition, and reduced M2 macrophage infiltration in mouse lung tissues induced by house dust mite. Yang and Li showed that EGCG treatment relieves asthmatic symptoms in mice by suppressing HIF-1α/VEGFA-mediated M2 skewing of macrophages [181]. Yang et al. [181] showed that EGCG effectively inhibits inflammation and infiltration of inflammatory cells as well as epithelial-mesenchymal transition (EMT) via the PI3K/Akt signaling pathway and the regulation of phosphatase and tensin homolog (PTEN) expression in the lungs of OVA-induced asthmatic mice. It appears that EGCG has protective effects on airway remodeling in asthma and may be useful as an adjuvant therapy for bronchial asthma [180,181,182]. The therapeutic potential of propolis is evident in allergic disorders caused by fungal allergens such as conidia of *Aspergillus fumigatus*, which cause allergic inflammation in people suffering from allergic bronchopulmonary aspergillosis (ABPA), allergic Aspergillus sinusitis and asthma [125,183]. The intake of propolis ethanol extract reduces the production of pro-inflammatory cytokines IL-13 and IL-17 and increases the production of IL-12. The inhibitory effect of propolis on the inflammatory response has been confirmed in various cell models, including epithelial cells and immune cells, such as peripheral leukocytes and PBMC [184]. El-Aidy et al. [184] reported similar data, namely that a propolis ethanol extract, at a dose of 30 mg/kg, reduced eosinophils and basophils in the blood and inflammatory cells in the peribronchial and perivascular regions in the lungs of conalbumin-induced asthmatic mice. In another study, Piñeros et al. [185] observed that a standardized extract of Brazilian green propolis (EPP-AF^®^) at a dose of (150 mg/kg), after oral administration in OVA-induced asthmatic mice, inhibited lung inflammation, mucus and IL-5 production, eosinophil count and reduced the Th2 response by increasing the number of polymorphonuclear-myeloid suppressor cells (PMN-MDSC) and CD4+ Foxp3+ regulatory T cells. Other studies [186,187] have shown the therapeutic effects of ethanol propolis extract on rat models with allergic rhinitis where oral propolis was more effective than intranasal propolis in reducing inflammation, vascular proliferation, and increasing goblet cells, and symptom scores (nasal irritation, sneezing, and the amount of nasal secretion). Propolis treatment inhibited histamine H1 receptor (H1R) gene expression levels and proinflammatory cytokines such as IL-4, IL-5 and IL-9 in the nasal mucosa of rats. In the treatment of asthma, propolis successfully reduced the development and proliferation of T-helper cells, the epithelial-mesenchymal transition, and expression of cytokines and chemokines that are crucial in the development of neutrophilic and eosinophilic inflammation [188]. It was demonstrated that propolis and the propolis-derived compounds, such as CAPE, pinocembrin, chrysin, appear to have activity against IgE-mediated allergy in systemic and local anaphylactic animal models. In addition, propolis and its components successfully reduced the response of IgE antibodies, the expression of histamine H-1 receptor and histamine levels in plasma, the activity of NF-κB, PAF, cys-leukotriene and the expression of TNF-α, IL-1β as well as the level of Th2 cytokines [188]. A certain formulation of propolis and N-acetylcysteine was shown to significantly reduce sputum production and subsequently increase the quality of life of patients with COPD, asthma, and bronchitis [189]. The formulation contained an aqueous extract of propolis collected in Denmark, China, Uruguay and Brazil, and was standardized to contain ≥ 0.05% of aromatic acids (mainly caffeic, ferulic, isoferulic, cinnamic, and 3,4-dimethoxy-cinnamic acids) and smaller quantities of flavonoids. Patients who received propolis showed an improvement in ventilatory functions after 2 months. The serum levels of the pro-inflammatory cytokines TNF-α, ICAM-1, IL-6, and IL-8 dropped by 52%, 65%, 44%, and 30%, respectively, whereas IL-10 increased 3-fold. PGE2, PGF2α, and LTD4 significantly decreased to 36%, 39%, and 28% of the initial values, respectively [190]. Propolis also has the potential to promote an anti-inflammatory environment in cigarette-smoke damaged lungs by restoring elastic fibers, inducing macrophage-alternative activation and Nrf-2, SOD, CAT, GPx activity and significantly reducing inflammatory cell counts, MPO activity, and oxidative damage [191,192].

Effects of flavonoids on MCC are shown in Table 10. Furthermore, it should be emphasized that the respiratory tract contains a stable and diverse microbial population within the extracellular mucus layer. Mucus provides a powerful defense against infection, and the maintenance of healthy mucus is critical for normal lung physiology, promoting immune tolerance, and enabling a healthy, commensal lung microbiome that can change in association with chronic respiratory disease. Mucus acts as a physical barrier by coating the respiratory tract (bar the terminal bronchioles and alveoli) to entrap microbes and facilitate their extrusion through MCC and cough reflex.

#### 5.2.6. Benefits of Polyphenols on Gut Microbiota

*Development of Microbiota and Its Importance in the Regulation of the Immune Response to Allergic Diseases*

A complex microbial ecosystem is found in the human body. The composition of the microbiota differs according to the anatomical site, such as the oral and nasal cavity, colon, vagina and skin [193]. This complex microbial ecosystem is separated from the host’s interior by a single layer of epithelial cells present in the gastrointestinal tract, respiratory tract, genitourinary tract, and skin. Epithelial cells act as an interface between the host and the environment and are equipped with specialized apical surfaces (microvilli, cilia, mucus production, intercellular junctions) to enable physiological function during contact with the microbiota. Therefore, intestinal homeostasis is defined as the establishment of a balance between the intestinal microbiota, the immune system and the integrity of epithelial cells. The highly complex ecosystem of the gut microbiome includes eukaryotic fungi, viruses, and archaea. Bacteria are the most dominant components in the human microbiome. They make up to 90% of the cells in a ratio of 10:1. The microbiome’s composition is highly dynamic and dependent on host-associated factors such as age, diet, and environmental conditions [194,195,196,197,198] with the major phyla being *Actinobacteria*, *Bacteroidetes*, *Firmicutes*, and *Proteobacteria*. Diet, age, stress, antibiotics, xenobiotics, viruses, bacteria, parasites and diseases increase or decrease the relative abundance and diversity of bacterial species in the GI and other body sites. Alterations in GI tract bacterial levels or diversity (dysbiosis) can disrupt mucosal immunological tolerance, leading to allergic diseases including food allergy (FA) [195,199] and asthma and other infectious disorders [195,196,197,198,199,200,201,202]. Allergic diseases include heterogeneous inflammatory pathologies, such as respiratory and food allergies (FA), which are characterized by an immunological response with Th2 lymphocytes producing IL-4, IL-5, and IL-13 and low production of IFN-γ [195,199] and (Th9) producing IL-9 and IL-10 [196] as the main effector T cells. They promote the induction of other effector cells involved in allergic inflammation, such as mast cells, basophils, and eosinophils [197,198]. According to reference [203], different levels of host-microbe interactions can be distinguished including: (1) microbe-gut epithelium interaction; (2) microbe-immune system interaction; (3) microbe-microbe interaction.

**Figure 5:**
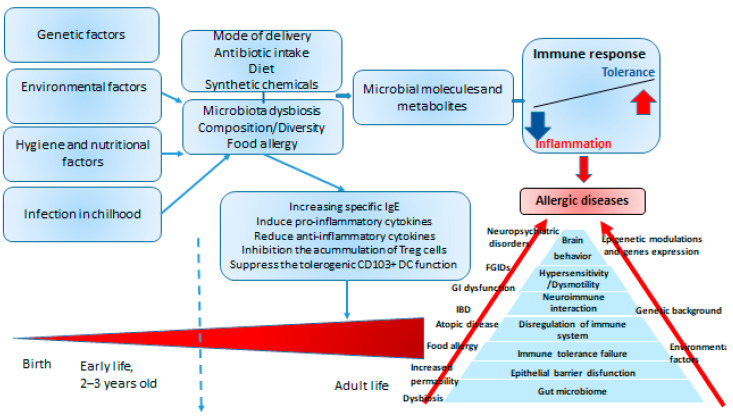
Numerous factors affect the development of the infant gut microbiome composition. The neonatal microbiome is a delicate and highly dynamic ecosystem that undergoes rapid changes in composition in the first few years of life determined by several prenatal (maternal genetic, environmental, diet, use probiotics, prebiotic, geographic location); natal (mode of delivery vaginal or cesarean, birth weight, gestational age) and postnatal factors (breast milk, use of antibiotics or gastric acidity inhibitors, use of antiseptic agents, rural environment, junk food-based and/or low-fiber/high-fat diet, consumption of unpasteurized milk or fermented foods, reduced consumption of omega-3-polyunsaturated fatty acids or vitamin D insufficiency, antioxidants, exposure to pets). All genetic, environmental, and dietary factors could modulate the gut microbiome-immune system axis influencing the occurrence of food allergy. The process of early colonization by a “healthy” microbiome is emerging as a key determinant of life-long health. In stark contrast, the perturbation of such a process, which results in changes in the host-microbiome biodiversity and metabolic activities, has been associated with greater susceptibility to immune-mediated disorders later in life, including allergic diseases.

In fact, it has been reported that dietary alterations are responsible for 57% of the gut microbiota’s entire variation, whereas the genetic background may only contribute to 12% [204]. With regards to the disturbance of intestinal microflora, dysbiosis may increase the individual’s susceptibility to infections and diseases such as allergy, inflammation, cancer and cardiovascular diseases. Food contaminants entering the body through the oral route are directly exposed to the action of gut microflora, which play a considerable role in the metabolism of endogenous and exogenous substances in the diet, food digestion, xenobiotic metabolism and regulation of innate and adaptive immunological processes. Imbalance of gut bacteria composition could be important in the context of health or disease [195,196,197,198,199,200,201]. Thus, early-life exposure is critical for the microbiome development. and microbiota can modulate the immune response [195]. Many studies have shown that children exposed early in life to microbiota present in farming/rural environments develop fewer allergic conditions than children living in the inner-city [204,205]. During pregnancy, the maternal and fetal immune system communicate in a bidirectional manner (Figure 5). The development and maturation of the immune system starts early in fetal life and continues throughout infancy and early childhood. In contrast, as a reduction in microbial diversity, dysbiosis induces intestinal inflammation and quantitative and/or qualitative abnormalities of the microbiota that may be associated with systemic immune, allergic, metabolic, and infectious disorders [195,204,205] as shown in Figure 5.

In the airways, quantitative changes in the microbiota have been described in chronic obstructive pulmonary disease, asthma and cystic fibrosis [206,207,208]. For example, cesarean-born infants typically have a gut microbiome, with lower levels of *Escherichia coli*, *Bifidobacterium* and *Bacteroides* species compared with those delivered vaginally [206], and increased levels of *Staphylococcus* and *Streptococcus*, comparable with the maternal skin microbiome [207]. The infant gut microbiota is significantly affected by factors such as perinatal exposure to maternal and child nutrition, antibiotic use and contact with older siblings while prebiotic intake as fibers and oligosaccharides can improve immunity and metabolism [200,204,205,206,207,208,209,210] and promote a long-term protective effect against food allergies in the offspring. For example, early inoculation with spore-forming *Clostridium* class IV and XIV species and other bacteria has been shown to lead to decreased levels of circulating IgE in adulthood while colonization of *H. influenzae, S. pneumoniae*, or *M. catarrhalis* within the first month of life increases the risk of asthma, leading to high counts of atopic markers, such as eosinophils and serum IgE [209,210,211]. Moreover, rhinovirus infection can also induce mucus hypersecretion and airway hyperresponsiveness in neonatal mice compared to adults [212]. According to Yatsunenko et al. [211], the highest interindividual microbial variability occurs during the first 3 years of age. It should be noted that the homeostasis of mucosal tissue arises from the perinatal establishment of mucosal-induced immune tolerance, which is controlled by signals from innate immune cells that form an adaptive immune response. Through its barrier function, cell contact-mediated signals and production of cytokines, this regulatory immune network is controlled by the mucosal epithelium. Perinatal maturation of the mucosal immune system may also be modulated by direct or undirect effects of environmental factors [195,196,197,208,209,210,211,212].

Environmental factors mainly modulate the gut microbiota structure and function, which in turn could be responsible for the epigenetic regulation of genes involved in immune tolerance. Thus, low IgA levels at the intestinal surface barrier can also contribute to FA. In fact, gut microbiota can stimulate DCs in the Peyer’s patches to activate B cells, leading to specific IgA antibody production through class switching [213,214]. Follicular T cells also activate B cells, inducing the production of IgA antibodies. Furthermore, microbe-immune system interactions include immune regulation and immune functions macrophages, neutrophils, DCs, T and B cells involved in the immune tolerance network. Therefore, the GI tract plays a very important role in the control of immune function in the development of either effector or tolerant responses to various antigens through the relationship between the activity of Th1 and Th2 cells as well as the regulation of Th17 and T regulatory (Treg) cells in the lamina propria [214,215,216]. Short-chain fatty acids (SCFAs), the main bacterial products of the catabolism of carbohydrates and proteins in the gut, and antigens present in food are the main antigenic load in the GI system. As metabolic products, SCFAs are crucial in the stimulation of macrophages and DCs and activation of Treg cells. Immune dysfunction in allergic diseases, such as asthma and atopic dermatitis, appears to be associated with differences in the function and composition of the gut microbiome [195,214,215]. Thus, in chronic inflammatory disorders of the skin, such as AD and psoriasis, the disorder occurs as intestinal dysbiosis, while the microbiome in the lungs triggers changes in the polarization of asthmatic cells by adjusting the balance between Th2 and Th17 patterns. According to Pascal et al. [195], the microbiome can be considered a therapeutic target for treatment of inflammatory diseases, such as allergies. It seems that probiotics, prebiotics, and/or symbiotics may be promising for the development of preventive therapies by restoring altered microbiome functionality, or as adjuvants in specific immunotherapies.

*Probiotics and Prebiotics and/or Symbiotics Can Influence the Intestinal Microbiota and Modulate the Immune Response*

Prebiotics are substrates, typically indigestible carbohydrates, in foods that selectively stimulate the growth and metabolic activity of beneficial intestinal microbes and are used by the microorganisms, providing health benefits to the host. Fiber and oligosaccharides can improve immunity and metabolism [200,204,205,206,207,208,209,210], and their intake in pregnant and lactating mice increases the effect of *Lactobacillus* and *Clostridium leptum* and promotes a long-term protective effect against food allergies (FA) in the offspring [204,205,206,207,208,209,210]. Therefore, it is important to enrich the diet with substances that promote the growth of lactic acid bacteria, which are important in maintaining human health. These bacteria have the ability to stimulate the immune system, protecting the host from the invasion of bacteria, viruses and toxins, reduce the inflammatory response through their antioxidant capacity, help the digestion of food to which humans are not tolerant, and concomitantly, create unfavorable conditions for the growth of potentially pathogenic species [195,214,215,216,217,218,219] producing strong acids as end products of metabolism (acetate, lactate), organic acids, hydrogen peroxide, and bacteriocins. Lactic acid bacteria also play a role in the synthesis of B vitamins, folic acid, vitamin K and lysozyme, as well as in metabolizing bile acids, sterols and xenobiotics [214,215,216,217,218,219]. Furthermore, as dietary supplements, lactic acid bacteria may help prevent constipation and regulate transit time, prevent diarrhea and colonic cancer, reduce blood lipid levels and stimulate the immune system by increasing the phagocytic activity of macrophages, and T and B cells. Probiotic bacteria can stimulate Th1 cells and production of IL-12 and IFN-γ. Cytokines, such as IL-12 and IFNs, produced by DCs and NK cells in response to stimulation of their TLRs by microbial products not only induce the Th1 differentiation but are also able to shift the allergen-specific Th2 response to a less polarized, or even to a Th1-polarized, phenotype [220]. Thus, the most important mechanisms in suppressing an allergic reaction could be based on modulating TLR and proteins that recognize enterocyte proteoglycans, leading to DC activation and Th1 response, stimulation of the Th1 cytokine and suppression of the Th2 response, increased IFN-γ production and decreased IgE and antigen-induced TNF-α, IL-5 and IL-10 secretion.

*Propolis and Its Flavonoids as an alternative Functional Food, Non-Pharmaceutical Approach in Maintaining Human Gut Health and Promotion of Gut Microbial Homeostasis*

Presently, allergic diseases, atopic eczema, allergic rhinitis, and asthma, besides chronic inflammatory bowel disease, Crohn’s disease, ulcerative colitis, diabetes and arthritis are chronic diseases that are on the rise in industrialized countries worldwide. The causes of these inflammatory diseases are unknown, although the explanation is partially linked to two hypotheses: the “hygiene hypothesis” and a reduced exposure to microorganisms and an underdeveloped immune system; another hypothesis is impairment of the gut-barrier function. Propolis and its components are strongly involved in preserving the intestinal barrier function, increasing the diversity, strengthening good intestinal microbiota, and reducing the number of pathogenic bacteria and their enzymatic activity. A recent study by Xue et al. [221] reported a significant increase in gut microbial diversity after 21 days of 0.3% propolis supplementation in rats fed with a Western diet [221]. Propolis treatment modified the dysbacteriosis caused by diabetes and restored the homeostasis of the gut microbiota microecology. Alongside this, propolis exerted hypoglycemic effects in diabetic rats, and repaired intestinal mucosal damage, benefited the communities of the gut microbiota and increased SCFA levels in diabetic rats through maintenance of transmembrane barrier proteins, such as occludin and claudins, which are components of tight junctions and gap junctions of the intestinal epithelium [222]. Furthermore, propolis plays an important role in the architecture and physiology of the tight junctions by increasing intracellular scaffold proteins ZO-1, which bind transmembrane barrier proteins to actin and microtubules [222,223,224,225]. Propolis also improves the metabolism of diabatic rats by upregulation of *Lactobacillus*, *Blautia* and *Clostridium* XVIII. Abundance of *Lactobacillus, Blautia* and *Clostridium* XlVa was significantly correlated with the concentrations of acetic acid, propionic acid and butyric acid, whereas the abundance of *Streptococcus, Enterococcus* and other bacteria was negatively correlated to a significant extent with the content of SCFAs. SCFAs stimulate secretion of intestine-trophic glucagon-like peptide to affect the intestines and reduce gut permeability [222,226] while butyric acid promotes synthesis of intestinal mucosa, which is beneficial to its repair and function. Propolis modulates gut microbiota, reduces endotoxemia and TLR4 pathway expression in mice fed with a high-fat diet [227]. Furthermore, the abundance of *Faecalibacterium* and the fecal butyrate content after consumption of polyphenols in obese rats improved intestinal barrier function by increasing the expression of zonula occludens 1 (ZO-1), occludin and mucin 2 (Muc-2) genes [228]. It has been shown that dimethoxy resveratrol derivatives also affect gut microbiota, especially the abundance of *Akkermansia muciniphila*, leading to an improvement in insulin sensitivity [229,230,231]; on the other hand, a natural flavanone glycoside increased the genus *Akkermansia* and expression of tight junction proteins, and reduced metabolic endotoxemia in mice fed with a high-fat diet [231,232]. Supplementation with resveratrol, quercetin and other flavonoids increased the abundance of *Lactobacillus* and *Bifidobacterium* and decreased the genes’ expression related to fatty acids synthesis, adipogenesis and lipogenesis in mice fed with a high-fat diet [231,232,233,234,235]. Catechins prevented an increase in toll-like receptor 4 (TLR4)/NFκB-dependent inflammatory genes [233]. Wang et al. [234] found that Brazilian propolis increased the diversity and richness of gut microbiota populations against dextran sulfate sodium (DSS)-induced colitis in rats.

Proanthocyanidin (PA), a major constituent of grape-seed polyphenols, exhibited beneficial effects on the gastrointestinal tract, including enhancement of bacterial flora in the intestine, prevention of colorectal cancer, and decreased prevalence of *Helicobacter pylori* [236,237,238]. Proanthocyanidin administration significantly suppressed gastric mucosal injury, induced by water-immersion restraint stress, in a dose-dependent manner. Mechanisms to inhibit mucosal damage by PA may be the following: (1) inhibition of acid secretion via G cells to circumvent attacks of water immersion restraint stress, (2) enhanced PGE2 secretion, (3) increased SOD activity to augment mucosal protection, (4) reduced secretion of gastrin, somatostatin, and histamine, and (5) inhibited myeloperoxidase activities. These interactions appear to inhibit acute stress-induced gastric mucosal injury.

Thus, the consumption of polyphenols, as prebiotics, especially catechins, anthocyanins and proanthocyanidins, which are non-absorbable oligomeric and polymeric components, increases the abundance of *Lactobacillus*, *Bifidobacterium*, *Akkermansia*, *Roseburia*, and *Faecalibacterium* spp. Intake of polyphenols, such as anthocyanins and ellagic acid, increased the production of SCFAs, including butyrate, and reduced plasma lipopolysaccharide-binding protein in animals. A similar effect was achieved with addition of vanillin in mice fed with a high-fat diet, where the increased production of acetate, propionate and butyrate and lower concentration of inflammatory factors (LPS, IL-6 and TNF-α) were observed [239]. Other beneficial effects have been observed with pro-anthocyanidin intake, including weight reduction, decreased insulin sensitivity, downregulation of pro-inflammatory genes in the liver and upregulation of genes involved in lipid catabolism. In addition, prebiotics can increase the level of butyrate and reduce the level of circulating inflammatory biomarkers, given that they can directly ferment to SCFAs, or indirectly ferment via cross-feeding, where the fermentation products of several microbes are utilized by others.

The anti-inflammatory mechanism of flavonoids for intestinal inflammation may be based on: (1) increased Treg activity and regulatory differentiation of Th17 cells in the lamina propria; (2) reduced infiltration of innate immune cells; (3) beneficial effect on the gut microbiota by inhibiting pathogens and promoting beneficial bacteria; (4) improved intestinal barrier function; (5) interference with lipid raft assembly by reducing cholesterol or preventing cholesterol oxidation; (6) suppression of TLR4; (7) reduced IkB phosphorylation; (8) inhibition of NF-kB; (9) inhibition of pro-inflammatory cytokines and enzymes, (10) inhibition of MAPK family phosphorylation, (11) upregulation of the Nrf2 pathway and expression of antioxidant enzymes.

A few studies have evaluated the effect of fiber/oligosaccharide intake in modulating asthma [200,240,241] with varying effects; one study reported reduction in wheezing [200,241,242] but others did not report any effects [242,243]. A recent Cochrane review showed that addition of prebiotics to infant food may reduce the risk of eczema, but it is not clear whether their use may affect other allergic diseases, including asthma [244].

There is evidence of an additional mechanism by which polyphenols protect the intestinal barrier against oxidative stress, since excess ROS will damage the cell membrane and disrupt the tight junctions leading to an increased intestinal permeability and development of metabolic disorder. According to references [216,217,244,245,246,247,248], probiotic strains are involved in the scavenging of hydroxyl radicals and superoxide anions and produce antioxidants, such as glutathione transferase, CAT, superoxide dismutase (SOD), GSH, folate, uric acid and vitamins C E, and β-carotene, which are absorbed and distributed in the organism. SOD production can increase the inherent cellular antioxidant defense as it prevents the formation of the toxic superoxide anion catalyzing its dismutation to hydrogen peroxide. *Lactobacillus* and *Bifidobacterium* are strains that have the ability to secrete SOD enzymes and metal chelating molecules and antioxidants that protect the intestines, liver and kidneys from diseases related to oxidative stress including cancer [216,217,244,245,246,247,248]. In addition, exopolysaccharides released by probiotic bacteria potentially play a role in reducing oxidative stress and stimulating Nrf2 expression in the liver [244,245,246,247,248]. The increased number of *Bifidobacterium* colonies in the intestines contributes to the anti-atherogenic properties of the flavonoids present in propolis as well as protection of the liver and intestines [216]. Moreover, probiotics can inhibit intestinal pathogens and reduce postprandial lipids, which are involved in oxidative damage [216,217,244,245,246,247,248]. The release of functional phenolic constituents after microbial fermentation in the colon contributes to colon health through their antioxidative, anti-inflammatory and immuno-modulatory effects. Through these prebiotic-effects, (poly)phenol-rich foods can attenuate metabolic and inflammatory diseases, increase host intestinal mucus production, induce the secretion of gut antimicrobial peptides, modulate hepatic bile acids, and gut immunoglobulins secretion [11,12,13,19,20,21,22,23]. Probiotic bacteria (*Lactobacillus rhamnosus* GG (LGG), *Lactobacillus plantarum* CAI6, *Lactobacillus casei*, *Lactobacillus plantarum* SC4, *Lactobacillus paracasei* Fn032, *Lactobacillus acidophilus*, *Lactobacillus bulgaricus* DSMZ 20080 and *Bifidobacterium longum*, *Bifidobacterium lactis* and *Bifidobacterium breve)* appear to have a role in preventing ROS production [216,217,244,245,246,247,248]. The total antioxidant activity of *Lactobacillus* strains was higher than other examined strains [249]. According to reference [250], LAB antioxidant activity depended on their concentration; LAB developed protective properties when the titer was equal to 3 × 10^7^ cells/mL. It seems that the protective effect of polyphenols against oxidative stress is based, in part, on its prebiotic properties through restoration of the intestinal microbiota [251].

There is evidence that colonic probiotic bacteria, such as *Lactobacillus acidophilus*, *Lactobacillus GG*, *and Bifidobacteria* [145,173,184,216,217], may positively modulate certain colon microbial enzymes that play a role in diet-related diseases [173,216,217]. For example, β-glucuronidase is an inducible enzyme elaborated by anaerobic *E. coli*, *Peptostreptococcus*, *Bacteroides* and *Clostridia*. Increased activity of this enzyme has been implicated in increased enterohepatic recirculation of toxins, hormones, drugs, and carcinogens. Therefore, reactivation of the released xenobiotics and endobiotics resulted in toxicity (diarrhea or epithelial injury) in the gut and increased the lifetimes of these compounds in the circulation. In fact, excessive β-glucuronidase activity may be a primary factor in the etiology of colon cancer.

**Figure 6:**
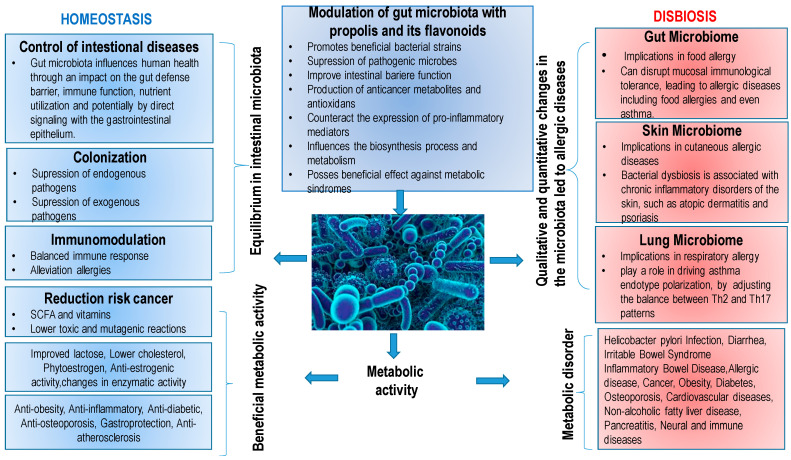
The main benefit of propolis and its flavonoids as prebiotics and their potential mechanism of action on the intestinal microbiota under physiological and pathological conditions, such as allergic diseases and metabolic disorders.

It has been suggested that increased *Lactobacillus* and *Bifidobacteri*, FOS, high-fiber diet and lowering colonic pH may help reduce β-glucuronidase levels [173,217]. According to Little et al. [251], glucuronidase activity may be regulated by the *Enterobacteriaceae* family of *Proteobacteria*, including *Escherichia*, *Salmonella*, *Klebsiella*, *Shigella*, and *Yersinia* pathobionts that can cause inflammation and changes in the intestines, leading to alterations in the mucosal epithelium and loss of structure of both villi and submucosa [217]. On the other hand, an increase in the number of *Bifidobacterium* colonies may be associated with a decrease in the count of *Enterobacter* colonies [200] as well as a reduction in the activity of β-glucosidase, β-galactosidase, and β-glucuronidase. Quercetin, which is found in propolis, fruits and vegetables, might act as a metabolic prebiotic and exert significant beneficial effects on the intestinal environment by modulation of the number of *Bifidobacteria* in cecal microflora population. The number of *Bifidobacterium* colonies increased after the intake of quercetin compared to the control [173]. It was found that select strains of *Bifidobacteria*, which are active in the biotransformation of flavonoid glycosides and are present in common bean seeds and seedlings, can improve the nutritional and health properties of flavonoid-based products. There is a double interaction between polyphenols and the gut microbiota; intestinal microbiota can transform dietary polyphenols into bioactive compounds and increase their bioavailability and provide beneficial effects on health, whereas polyphenols and their metabolites may modulate the growth and/or metabolic activity of the intestinal microbiota, and thus, its composition and function (Figure 6). It has been shown that more complex unabsorbed polyphenols and their metabolites selectively inhibit the growth of pathogens and stimulate growth of *Lactobacilli* and *Bifidobacteria* while the intake of prebiotics increases the number and metabolic activity of LAB in the colon of humans and animals [145,173,184,216,217,252]. Furthermore, lower activity of pro-carcinogenic enzymes was also shown as a consequence of prebiotic intake in humans. Quercetin preparations alone and/or in combination with ochratoxin, mycotoxins produced by certain *Aspergillus* species significantly suppressed bacterial β-glucuronidase activity in faces and caecal digest [173]. This is important because many of the glucuronides are conjugated products of detoxification by the liver, and their hydrolysis may liberate free toxins including carcinogens, which may then act on the colonocytes or may be absorbed by the intestines [173,252]. Reported data suggest that inhibition of β-glucuronidase in the intestinal microflora and the beneficial effects on lactic acid bacteria are major mechanisms of quercetin protection.

It is important to note that inhibition of β-galactosidase by propolis and flavonoids may reduce release of glucose and monosaccharides from carbohydrates, which play a potential role in controlling blood sugar levels resulting in anti-hyperglycemia [97]. EEP increases HDL-c levels, while reducing LDL-c, and reduces oxidation of LDL by two different antioxidant mechanisms, such as activation of the transcription factor Nrf2 and enhancement of the antioxidant enzymes such as heme oxygenase-1, phase II detoxification enzymes, and enzymes involved in GSH metabolism [97]. The secondary mechanism involves neutralization of oxidative species and inhibition of the NF-κB signaling pathways. Reduced levels of LDL-c in HFD mice treated with EEP is the beneficial aspect and shows the potential anti-atherosclerotic effects of EEP. This observation was consistent with the atherogenic parameter indices leading to protection against cardiovascular diseases, lipid disorders, diabetes, obesity and other complications. The positive effects of propolis are induced by its bioactive components, such as reservatol, quercetin, chrysin, naringenin, CA, CAPE, proanthocyanidins, gallic acid, rosemary acid, isorhamnetin, camphor, lutein, and pinocembrin.

On the other hand, Gardana et al. [253] described a strategy using food grade materials to selectively and effectively reduce the allergenic molecules in propolis, such as CA and its esters by means of a bacterial biotransformation procedure based on the cinnamoyl esterase activity of *Lactobacillus helveticus*. As previously noted, CA and its esters have been suggested as important allergens in propolis, as both CAPE and caffeic acid 1,1-dimethylallyl ester were strong sensitizers in guinea-pig experiments. Alongside its numerous positive properties, propolis may also have adverse effects, such as causing allergic eczematous contact dermatitis in apiarists and consumers with an allergic predisposition. For this reason, a strategy to eliminate allergens from propolis would be of wide interest. Thus, Gardana et al. [253] showed that a reduction in caffeate esters by *L. helveticus* did not affect the content of flavonoids and did not cause loss of the antimicrobial activity of propolis. This method is based on a foodgrade solvent (ethanol or PEG 400) and the cinnamoyl esterase activity of *L. helveticus*, a dairy bacterium generally recognized as safe; it is included in the EFSA QPS list of microorganisms and demonstrated probiotic properties. This strategy resulted in a propolis extract that would be industrially available.

Given that flavonoids are indigestible components of food, they have a positive effect on probiotic bacteria. Their major effect to improve clinical symptoms in patients with allergic diseases and prevent them, include: (1) inhibition of Th2 responses and shift responses to Th1; (2) production of butyrate and increased induction of tolerance; (3) increase in regulatory and anti-inflammatory cytokines, such as IL-10, and decrease in inflammation; (4) reduction in the number of eosinophils and serum specific IgE levels; (5) increase in the IFN-γ/IL-4 ratio; (6) increase in Treg cells and induction of their responses; (7) increase in TGF-β responses and inhibition of allergic responses; and (8) decrease in the metalloproteinase activity and cell infiltration.

## 6. Effects of Flavonoids on Specific Allergic Reactions

**Figure 7:**
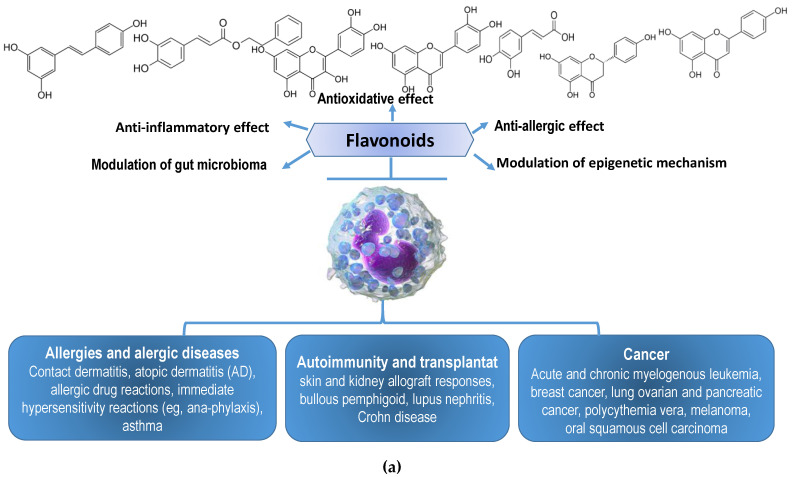

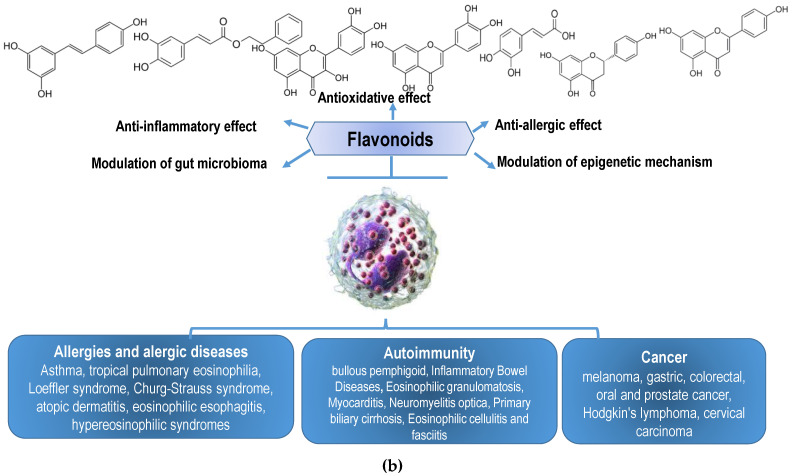
Propolis and flavonoids as candidates for preventing the development of inflammatory diseases, allergies, autoimmunity and cancer or as supplements to the management of basophils (**a**) and/or eosinophil-mediated diseases (**b**). Propolis and its flavonoid components can regulate basophils and/or eosinophil-mediated diseases by their antioxidant, anti-inflammatory, anti-allergic effects or through modulation of intestinal microbiota and epigenetic mechanisms.

Propolis and related flavonoids have been shown to control specific allergic reactions including allergic rhinitis, asthma, different skin allergy, rheumatoid arthritis, atopic dermatitis, bronchitis, ocular allergy and others [125,136,154,254,255,256,257]. Thus, flavonoids, can regulate a number of allergic, autoimmune and inflammatory diseases, including cancer, through blockade of eosinophils and basophils, as important mediator cells in allergy and inflammation (Figure 7a,b).

Propolis and its flavonoid components have confirmed their effects against allergic inflammations, allergic rhinitis, asthma, atopic dermatitis and food allergies and inflammatory bowel and skin diseases in numerous in vitro studies on different cell models, and animal models. Their anti-inflammatory and anti-allergic effects are attributed to the inhibitory effect of propolis on cells and their mediators involved in the inflammatory process, including activation of epithelial cells, mast cells, basophils and eosinophils and the release of various allergic mediators and cytokines. The most important active ingredients of propolis with anti-allergic properties are flavonoids, such as quercetin, chrysin, kaempferol, galangin and pinocembrin and cinnamic acid derivatives, such as CAPE and artepillin. Liew et al. [125] reported data on preclinical and clinical use of propolis as an adjunct in allergy therapy. As a supplement or an adjunct therapy, propolis is safe and attenuates various pathological conditions in asthma, atopic dermatitis, allergic rhinitis, and food allergies. The authors suggested that the anti-allergic activities of propolis may not be present in all propolis samples due to variations in chemical composition. Furthermore, these data can promote further research regarding the possible protective effects of propolis and flavonoids in preventing allergic diseases and the isolation of propolis components that cause sensitization to propolis in a larger percentage of people. Considering certain epidemiological data and previous research, propolis and its active components may provide a form of complementary and alternative medicine in the prevention and treatment of allergic diseases.

## 7. Conclusions

Propolis exerts anti-inflammatory/antiallergic activity, likely due to its antioxidant and anti-inflammatory properties. The protective role of propolis (via inhibition of the ROS formation pathway) occurs through retardation of NF-κB activation, inhibition of eicosanoid synthesis, reduction in expression of various inflammatory cytokines, and inhibition of oxidative damage to proteins, lipids, DNA/RNA and carbohydrates. The main components of propolis with anti-inflammatory properties are CA, quercetin, naringenin, caffeic acid phenethyl ester, apigenin, ferulic acid, and galangin. The flavonoid components of propolis are effective in inhibiting LOX activity and the inducible isoform of COX (COX-2) activity, reducing the release of prostaglandins and leukotrienes and nitric oxide. In addition, propolis and its flavonoids have inhibitory effects on the activity of myeloperoxidase, NADPH-oxidase, ornithine decarboxylase and tyrosine-protein kinase and hyaluronidase. Thus, the administration of propolis and its flavonoids may provide a viable strategy for mitigating allergic symptoms. Furthermore, polyphenols/flavonoid compounds may regulate Th1/Th2 balance and cytokine profiles and thus inhibit antigen-specific IgE antibody production by influencing the formation of allergen-IgE complexes and binding of this complex to its receptors (FceRI) on mast cells and basophils. Furthermore, the intake of polyphenols as prebiotics, especially catechins, anthocyanins and proanthocyanidins, increases the number of *Lactobacillus*, *Bifidobacterium*, *Akkermansia*, *Roseburia*, and *Faecalibacterium* spp. and production of SCFAs, including butyrate, acetate and propionate, which help maintain intestinal homeostasis and health. Maintaining intestinal homeostasis is important for normal functioning and stimulation of the immune system, protecting the host from the invasion of pathogenic bacteria, viruses and toxins, changing signaling pathways in the host’s cells, reducing the inflammatory response and promoting digestion of intolerant food. It should be noted that alterations in the GI tract bacterial levels or diversity (dysbiosis) can disrupt mucosal immunological tolerance, leading to allergic diseases including food allergies. These bacteria create conditions unfavorable for the growth of potentially pathogenic species, inhibit bacterial adherence to the mucosal layer, enhance epithelial barrier integrity and improve barrier function and mucus production affecting allergic diseases by physiological and immunological mechanisms. Host-microbe interactions undoubtedly are complex; however, a change in dietary habits and a higher intake of polyphenol components can regulate the gut microbiota to favor growth of beneficial bacteria and prevent growth of pathogenic bacteria. Thus, dysbiosis is reduced and consequently, the barrier integrity whose excessive permeability affects the release of inflammatory mediators including ROS and RNS, is maintained.

Propolis is a non-toxic natural product; however, certain cases of allergy and contact dermatitis have been described, mainly among beekeepers. Allergic reactions may manifest as contact cheilitis, contact stomatitis, perioral eczema, labial edema, oral pain, peeling of lips, and dyspnea. For the safe use of propolis preparations, hypoallergenic propolis without CA esters should be developed or sensitivity to propolis should be checked with a skin test before use. Moreover, propolis has a promising potential to be further developed for prophylactic or therapeutic use in allergic diseases, such as asthma, allergic rhinitis, and AD, and its therapeutic benefits are well-supported by both preclinical and clinical studies. Similarly to prebiotics, propolis may be an effective alternative, considering the relationship between the microbiome and healthy aging without diseases such as inflammation, allergy, autoimmunity and cancer. In addition, propolis and flavonoids could alleviate and reduce numerous consequences of allergic diseases and improve quality of life. Regardless of the numerous positive effects, its availability, and economic profitability, it is still necessary to reconsider the potential applications of propolis and its flavonoids as therapeutic agents in allergies. The development of a new class of anti-inflammatory and anti-allergic agents based on flavonoid molecules may be realized in due time.

## Figures and Tables

**Table 1:** Gell and Coombs classification of hypersensitive reaction and some characteristics of the major of hypersensitivities.

Characteristic	Type I Hypersensitivity	Type II Hypersensitivity	Type III Hypersensitivity	Type IV Hypersensitivity; Delayed Cell-Mediated			
Descriptive name	IgE-mediated hypersensitivity	Antibody mediated cytotoxic hypersensitivity	Immune complex-mediated hypersensitivity	Cell-mediated hypersensitivity			
				Type IVa	Type IVb	Type IVc	Type IVd
Cell type responsible	B cells	B cells	B cells	Th1 cells	Th2 cells	T-cells	Neutrophils
Type of antigen	Soluble antigen	Cell- or matrix- associated antigen	Soluble antigen	Soluble antigen	Soluble antigen	Cell antigen	Soluble antigen
Type of antibody	IgE	IgG, IgM	IgG	none	IgE	none	none
Other cell involved	Basophils, mast cells	Red blood cells, white blood cells, platelets	Various host cells	Macrophage activation	IgE production, eosinophyls activation, mastocytosis	CTL	Soluble antigen presented by cells or direct activation of T cells
Mechanism	Ag induces cross-linkage of IgE bound to mast cells and basophils with release of vasoactive mediators	Ab directed against cell-surface antigens mediates cell destruction via complement activation or FcR^+^ cells (phagocytes, NK cells)	Ag-Ab complex deposited in various tissues induce complement activation and ensuing inflammatory response	Sensitized T_DTH_ cell release cytokines that activate macrophages or T_C_ cells, which mediate direct cellular damage	Sensitized T_DTH_ cell release cytokines that activate macrophages or T_C_ cells, which mediate direct cellular damage	Cytotoxic reactions (by CD4 and CD8 cells) withn cytotoxic T cells as effector cells which mediate direct damage	T-cell–dependent, neutrophilic inflammatory reaction
Effector mechanism	Mast-cell activation	Complement, FcR^+^ cells (phagocytes, NK cells)	Complement, Phagocytes	Macrophage activation	Macrophage activation	Cytotoxicity, perforine, granzime B	Activated neutrophils
Mediators	Histamine, serotonin	Complement, ADCC	Complement, neutrophil proteases	Cytokines, IFN-γ, TNF-α	Cytokines Il-4, Il-5, IL13	Cytokines	Cytokines, CXCL8, GM-CSF
Transfer of hypersensitivity	By serum	By serum	By serum	By T cells	By T cells	By T cells	By T cells
Initiation time	2–30 min	5–8 h	2–8 h	24–72 h	24–72 h	24–72 h	24–72 h
Time of reaction after challenge with antigen	Immediate, up to 30 min	Hours to days	Hours to days	Peaks at 48 to 72 h	Peaks at 48 to 72 h	Peaks at 48 to 72 h	Peaks at 48 to 72 h
Skin reaction	Wheal and flare	None	Arthus	Induration, necrosis	Induration, necrosis	Induration, necrosis	Skin peels off, desquamation
Typical manifestation, exampes	Systemic anaphylaxis (anaphylactic shock); Localised anaphylaxis: hay fever, asthma, hives, food allergy, eczema	Blood—transfusion reactions, erythroblastosis fetalis, autoimmune hemolytic anemia, allergy to some drugs (e.g., penicillin)	Localised Arthus reaction; Generalised reactions: serum sickness, glomerulonephritis, rheumatoid arthritis, systemic lupus erythematosus, malaria kidney damage	Contact dermatitis, tuberculin reaction	Chronic asthma, rhinitis	Contact dermatitis, graft rejection	Acute generalised exanthematous pustulosis (AGEP), Behcet diseases (rare vasculitic disorder)

**Table 2:** Common clinical features of adverse reactions to food.

Organ System	Clinical Features
Skin	Atopic dermatitis
	Urticaria angio-oedema
Gastrointestinal tract	Oral allergy syndrome
	Abdominal pain
	Colic, nausea
	Vomiting
	Diarrhoea
	Constipation
	Bloating
	Gastro-oesophageal reflux (heart burn)
	Enteropathies
	Failure to thrive
Respiratory tract	Asthma
	Rhinitis
Eyes	Conjunctivities
	Watering eyes
Central nervous system	Headache
	Abnormal behaviour (ADHD), rare
Generalised (systemic)	Anaphylaxis

**Table 3:** Main effects of probiotics.

Effects	Probiotic Bacteria	Mechanism of Action
Maintain the balance of intestinal microflora	Lactobacilli and bifidobacteria	- Maintain a healthy balance of intestinal flora by producing organic compounds such as lactic acid, hydrogen peroxide, and acetic acid that increase the acidity of the intestine and inhibit the reproduction of many harmful bacteria.
Inhibit the growth of harmful bacteria	Acidophilus, *Bifidobacterium bifidum*, *Bifidobacterium longum*, Intestinal Flora, *Lactobacillus acidophilus*, *Lactobacillus casei*, *Saccharomyces boulardii* *L. rhamnosus GG*	- Produce bacteriocins, which act as natural antibiotics to kill undesirable microorganisms
Improving good digestion		- Proteases and lipases production; of enzymes which help digest proteins, and fat
Strengthen immune function		- Stimulate Th1 cells and production IL-12 and IFN-γ
Strengthening resistance to infections		- Prevent vaginal yeast infection - Inhibition *Candida* overgrowth
Protection of intestinal bacteria	*Lactobacillus acidophilus*, *Lactobacillus rhamnosus* and *Lactobacillus plantarum*	- Involved in the production of several “gut nutrients,” such as short-chain fatty acids, and the amino acids, arginine, cysteine, and glutamine - Remove toxins from the gut and exert a beneficial effect on cholesterol levels
Reduce intestinal permeability and inflammatory cytokines	Lactobacilli	- Attenuate pro-inflammatory responses by regulating NFκB activity
Protect the digestive system from opportunistic bacterial infections	*Saccharomyces boulardii* *Lactobacillus rhamnosus* GG(ATCC 53013) *Bifidobacterium lactis* Bb-12	- Prevent new infections - Reduce “traveler’s diarrhea”
Alleviate the consequences of acute pancreatitis	*Lactobacilus plantarum*	- Reduce the number of complications after surgery
Tumor prevention	*Lactobacillus* *Lactobacillus casei Shirot*	- Reduce fecal enzyme activities - prevent superficial bladder cancer and cervical cancer
Intestinal recolonization during and after antibiotic use	*Bifidobacterium longum SBT 2928* *Lactobacillus rhamnosus* GG(ATCC 53013) *Lactobacillus GG strain*	- Prevent infections after antibiotic use - Inhibit adhesion of enterotoxigenic *Escherichia coli* strain Pb176 - Reduce fecal enzyme activities - Reduce antibiotic-associated diarrhoea in children and prevent rotavirus - relapsing *Clostridium difficile* diarrhoea, immune response modulation, and alleviation of atopic symptoms in children
Improve digestion of foods causing intolerance	Acidophilus, *Bifidobacterium bifidum*, *Bifidobacterium longum*, Intestinal Flora, *Lactobacillus acidophilus*, *Lactobacillus casei*, *Saccharomyces boulardii*	- Enzymes secreted by probiotic bacteria aid digestion - source of lactase, the enzyme needed to digest milk sugar
Production of key vitamins: K, B and folic acid, riboflavin (B2), folate (B11), and cyanocobalamine (B12)	*Lactococcus lactis*, *Lactobacillus gasseri*, *Lactobacillus reuteri* and *L. plantarum Bifidobacterium* (e.g., *B. adolescentis*)	- Vitamin K is required for blood clotting and healty wound healing, - Supports calcium balance and bone mineralization, - Beneficial effects on the heart and blood vessels and cognitive function - Riboflavin (vitamin B2) has an essential role in cellular metabolism, precursor of the coenzymes flavin mononucleotide (FMN) and flavin adenine dinucleotide (FAD) - Vitamin B12 is required for the metabolism of fatty acids, amino acids, nucleic acids and carbohydrates - Synthesize *de novo* and supply vitamins to human body
Alleviation of allergic diseases by physiological and immunological mechanisms	*Enterococcus faecalis*, *L. casei DN-114001*, *L. acidophil us L-92*, *LGG and B. lactis Bb12*, *L. casei strain shirota*, *L. rhamnosus*, *L. paracasei* *B. Ion gum BBS36* *B. Iongum BBS36* *B. lactis*	- **Physiological mechanisms include:** - Inhibition of bacterial adherence to mucosal layer, - Enhancement of epithelial barrier integrity and improved barrier function, - Increase in mucus production - **Immunological mechanisms:** - Affect the epithelial cells and modulate signaling pathways - Reduce the expression of inflammatory cytokines by suppressing NF-kB signaling - reduce proinflammatory cytokines (TNF-α, IL-1β, IL-6, and IL-8), and increase in IL-10 production - restoration of Th1/Th2 cytokine balance - Modulate toll-like receptors and the DCs, reduce of TLR4 and IL-1β mRNA levels and significantly increased mucosal IL-10 - Increase production of IFN-γ and decrease IgE and IgG1 levels - Reduce number of eosinophils and its activity - Decrease production of CCL2, CCL3, CCL5, CCL11, IL-4 and IL-5 - Stimulate DCs and Treg differentiation induction of CD4+ Foxp3+ Treg cells and production of TGF-β and IL-10, - Increase the production of secretory IgA and IgG4 by B cell and reduction in allergen-specific IgE by B cells. - Prevent antigen-induced Th2 immune responses - Suppress Th17 immunity and symptoms of allergic airway disease - Inhibit subsequent allergic sensitization and airway disease by induction of Treg cells with increased TGF-β. - Decrease levels of degranulated mast cells, eosinophil granules, and tail scabs. - Reduce matrix metalloproteinase 9 expression in lung tissue - inhibit inflammatory cell infiltration. - Decrease the mRNA levels of Th2 and Th17 cell transcription factors, - Increase the transcription factors of Th1 and Treg cells, galactin-9, filaggrin - Inhibit the lung inflammation and airway remodel by interfering on Th1/Th2 cytokines and STAT6/T-bet transcription factors.
Strengthen the immune defense and end immune resistance	*Bacillus coagulans, B. subtilis HU58* *Bifidobacterium adolescentis, B. animalis, B. bifidum, B. breve, B. infantis, B. lactis, B. longum, B. thermophilus* *Enterococcus faecium* *Lactobacillus acidophilus, L. brevis, L. casei, L. delbrueckii*, *L. fermentum, L. helveticus, L. johnsonii, L. lactis, L. paracasei, L. plantarum, L. reuteri, L. rhamnosus, L. salivarius*, *Lactococcus lactis* *Propionibacterium freudenreichii; P. jensenii* *Streptococcus thermophilus*	- Stimulation of local and systemic systems - enhanced defense response (increases IgA, CD4+ and CD8+ T cells, NK cell activity, adjuvant effect) - Downregulate inflammatory and allergic responses
Strengthen resistance to colonization		- Increase competitive exclusion (e.g., for nutrients, adhesion sites) - Decrease harmful microbial species - Increase epithelial cell mucin synthesis - Strengthen intestinal barrier function

**Table 4:** Eosinophils secrete a range of highly toxic granule proteins and other mediators of inflammation that are released by eosinophils.

Class of Product	Mediators	Biological Effect
**Enzyme**	Eosinophil peroxidase	Toxic to target by catalysing halogenation. Triggers histamine release from mast cells
	Eosinophil collagenase	Remodeling of conective tissue matrix
	Lizosim	Higly toxic granule proteins which can kill microorganisms and parasites
	Acid phosfatase	
	Arilsulfatase B	
	Catalase	
	Enoil-CoA hidratase	
	3-Ketoacyl-CoA tiolase	
	β-glucoronidase	
	Elastase	
	Katepsin D	
**Toxic proteins**	Major basic protein	Toxic to parasites and mammalian cells. Triggers histamine release from mast cell, Stimulation of neutrophils, Mast cells, and basophils, Respiratory epithelial desquamation, M2 receptor dysfunction
	Eosinophil cationic protein	Toxic to parasites. Neurotoxin, bronchial hyperresponsiveness, leads to bronchoconstriction, respiratory epithelial desquamation, cell and parasite toxicity, Generation of radical species, stimulation of mast cells
	Eosinophil-derived neurotoxin	Neurotoxin
**Cytokine**	IL-3, IL-5, IL-9, GM-CSF, IFN-γ, TNF-α, IL-2, IL-13, IL-16, IL-17, IL-4, IL-6, IL-8	Sustained inflammation, amplify eosinophil production by bone marrow. Cause eosinophil activation eosinophil migration, development, and survival
**Chemokine**	CCL2, CCL3, CCL5 (RANTES), CCL-7, CCL8, CCL11, CCL13, CXCL1, CXCL10, CXCL12, and IL-8	Promote influx of leukocytes Migration of monocytes, macrophages, neutrophils, T cells, and eosinophils Increase adhesion molecule expression, airway wall remodeling Increase eosinophil survival, increased adhesion molecules expression, airway wall remodeling
	eotaxin	Cause ckemotaxis for eosinophils
**Lipid mediators**	Leukotrienes (LTC4, LTD4, LTB4), tromboxan A_2_ (TXA^2^), prostagladins (PGE_2_, PGG_2_, PGF_2_, PGI_2_), Cysteinyl leukotrienes,	Smooth muscle contraction, increased vascular permeability, mucus secretion Increased mucus secretion, increased vascular permeability, activation of eosinophils, mast cells, basophils, neutrophils, and platelets, smooth muscle cell contraction Increased adhesion molecules expression Chemotaxis of eosinophil and neutrophil
	5-HETE; 12-HETE; 15-HETE (monohydroxyeicosatetraenoic acids); 5,15-diHETE; 8,15-diHETE; 14,12-diHETE	Play a vital role in inflammation by controlling the intesity and duration of pain, fever, swelling, and heat of an affected area
	Lipoxin A_4_ and C_4_ (LXA_4_, LXC_4_)	Inhibits PMN adherence, migration, degranulation, and superoxide anion generation, as well as eosinophil migration and lymphocyte activation, and has potent actions on cytokine formation and release. Blocks P-selectin mobilization induced by peptidoleukotrienes and attenuated P-selectin-mediated PMN-endothelial cell adhesion.
	13-hydroxilinoleic acid (13HODE)	
	Platelet-activating factor (PAF)	Chemotactic to leukocytes. Amplifies production of lipid mediators, neutrophil, eosinophil, and platelet activation.
**Growth factor of eosinophils**	Tumor necrosis factor α and β (TNF-α, TNF-β); Platelet-derived growth factor (PDGF); Vascular endothelial growth factor (VEGF); Heparin-binding epidermal growth factor (HB-EGF); Nerve growth factor (NGF); Endothelin (ET); Corticotropin releasing factor (SCF); A proliferation-inducing ligand (APRIL)	Control of maturation and differentiation of eosinophils

**Table 5:** Mediators of basophils.

Class of Product	Mediators	Examples of Function
**Granule-associated**	Histamine and serotonin	Alter vascular permeability, smooth-muscle contraction
	Heparin and/or chondroitin sulphate peptidoglycans	Enhance chemokine and/or cytokine function and angiogenesis
	Neutral proteases Tryptase, chymase, carboxypeptidase A, cathepsin G, elastase, and other proteases	Remodel tissue and recruit effector cells
**Growth factor**	VEGF and FGF2, SCF	Recruit effector cells and enhance angiogenesis
**Enzymes**	Matrixmetalloproteinases 9 (MMP9), β-hexosaminidases and heparanases, plasminogen activator, renin, β-glucuronidase Arylsulfatase	Tissue damage/remodelling
**Lipid-derived**	Prostaglandin D2 (PGE2) Leukotriene C4, D4, E4 (LTC4, LTD4,LTE4) Platelet activating factor	Vasodilation, bronchoconstriction, neutrophil chemotaxis Prolong bronchoconstriction, mucus secretion, increase vascular permeability, Bronchoconstriction, increase vascular permeability, chemotaxis of leukocytes
**Cytokine**	IL3, TNF-α, MIP-α IL-4, IL-6, IL-8, IL-13, IL-18, IL-33, Thymic stromal lymphopoietin (TSLP) IL-5	Promotes mast cell proliferation Inflammation Th2 differentiation Basophils regulation, IgE-independent disorders Promotes eosinophil production and activation
	IL-3, IL-4, IL-5, IL-9, IL-13, IL-15 and IL-16	Functions of T helper 2-type cytokines
	IL-12 and IFN-γ	Functions of T helper 1-type cytokines
	IL-10, TGF-ß and VEGF	Regulate inflammation and angiogenesis
**Chemokine**	MIP-1α (CCL3), MIP-1β (CCL4), CCL12, and Cxcl2, CXCL11, innate lymphoid cells (ILC2s)	Recruit effector cells, including DCs, and regulate immune responses
**Neuropeptides**	ACTH, CRF, urocortin, VIP	Inflamation, sensory nerve modulation, vasodilatation
**Other**	Nitric oxide and superoxide radicals Antimicrobial peptides	Bactericidal Bactericidal

**Table 6:** Main classes of mediators that are released by mast cells.

Class of Product	Mediators	Biological Effect
**Granule-associated**	Histamine and serotonin	Alter vascular permeability, smooth-muscle contraction
	Heparin and/or chondroitin sulphate peptidoglycans	Enhance chemokine and/or cytokine function and angiogenesis
	Tryptase, chymase, carboxypeptidase and other proteases	Remodel tissue and recruit effector cells
	TNF, VEGF and FGF2	Recruit effector cells and enhance angiogenesis
**Lipid-derived**	LTC4, LTB4, PGD2 and PGE2	Increased vascular permeability, vasodilatation, recruit effector cells, regulate immune responses, and promote angiogenesis, platelet aggregation, oedema and bronchoconstriction
	Platelet-activating factor (PAF)	Platelet aggregation and degranulation, contraction of pulmonary smooth muscles, activates effector cells, enhances angiogenesis and induces physiological inflammation
**Cytokine**	TNF, IL-1α, IL-1ß, IL-6, IL-18 GM-CSF, LIF, IFN-γ. and IFN-ß	Induce inflammation
	IL-3, IL-4, IL-5, IL-9, IL-13, IL-15 and IL-16	Functions of T helper 2-type cytokines
	IL-12 and IFN-γ	Functions of T helper 1-type cytokines
	IL-10, TGF-ß and VEGF	Regulate inflammation and angiogenesis
**Chemokine**	CCL2, CCL3, CCL4, CCL5, CCL11 and CCL20	Recruit effector cells, including DCs, and regulate immune responses
	CXCL1, CXCL2, CXCL8, CXCL9, CXCL10 and CXCL11	Recruit effector cells and regulate immune responses
**Neuropeptides**	Adrenocorticotropin hormone (ACTH), CRF, urocortin, vasoactive intestinal peptide (VIP)	Inflamation, sensory nerve modulation, vasodilatation
**Other**	Nitric oxide and superoxide radicals Antimicrobial peptides	Bactericidal Bactericidal

**Table 7:** Inflammatory mediators synthesized by and stored in platelets.

Class of Product	Mediators	Biological Effects
**Cytokine**	IL-1β, IL-1α,IL-7, Tumor necrosis factor α (TNF-α), CD40L, HMGB1, ENA-78 (CXCL5), SDF-1(CXCL12)	IL-1β, CD40L induces adhesion receptor expression on endothelium and release of chemokines Increse leukocyte recruitment
**Chemokine**	IL-8 (CxCL8), platelet factor 4-PF_4_(CxCL4), β-thromboglobulin (CXCL7/NAP-2), Regulated on Activation, Normal T Cell Expressed and Secreted (RANTES or CCL5), Macrophage Inflammatory Protein-MIP-1α (CCL3), MCP-3 (CCL7), NAP-2 (CXCL7) TARC (CCL17)	RANTES-promote leucocyte recruitment and potent eosinophil chemoattractant; MIP-1α is a chemoattractant for monocytes, macrophages, T-cells and neutrophils Recruits monocytes
**Enzymes**	Matrixmetalloproteinases (MMP), β-hexosaminidases and heparanases	Disruption of the composition and integrity of cell membranes by degrading glycoproteins, glycolipids and glycosaminoglycans
**Lipid mediators**	tromboxan A_2, P_GE2 and 12-HETE (12-hydroxyeicosatetraenoic acid)	Vasoconstriction; platelet activation and aggregation; smooth muscle contraction Vasodilation (PGE2), naive T cell priming (PGE2),chemotaxis (12-HETE)
**Growth factor**	Platelet-Derived Growth Factor (PDGF),Tumor necrosis factor β(TNF-β), epidermal growth factor (EGF), vascular endothelial growth factor (VEGF), growth-related oncogene (GRO-α or CXCL1)	Contribute to cellular proliferation and amplification of the inflammatory response. Proliferative actions on structural cells of the airways; PDGF, a chemoattractant for monocytes and eosinophils
**Polyphosphates**	Phosphates	Pro-inflammatory and procoagulant functions, accumulation of neutrophils and increased vascular permeability
**ATP**	Nucleotide	
**Serotonin**	Monoamine	Vasoactive mediator; stimulation of fibrosis, by increasing collagen synthesis by fibroblasts, as well as neurotransmission
Glutamate	Amino acid	

**Table 8:** The most important propolis types: geographical origin and major constituents.

Propolis Type	Geographic Origin	Plant Source	Typical Chemical Constituents
Poplar propolis	Europe, North America, non-tropical regions of Asia, New Zealand, China	*Populus* spp. (most often *P. nigra* L.)	pinocembrin, pinobanksin, pinobanksin -3-O-acetate, chrysin, galangin, phenolic acids, and their esters
Birch propolis	Russia	*Betula verucosa* Ehrh.	acacetin, apigenin, ermanin, rhamnocitrin, kaempferid, α-acetoxybetulenol
Green (Alecrim) propolis	Brazil	*Baccharis* ssp. (most often *B. dracunculifolia* DC.)	prenylated p-coumaric acids and o-hydroxy-acetophenone, labdane, diterpenic acids
Red propolis	Cuba, Mexico, Brazil, Venezuela, Amazon	*Clussia *ssp. (?) *Clusia* flower *D. ecastophyllum* and other Dalbergia species	phenylpropene derivative elemicin, triterpenic alcohol β-amyrin, prenylated benzophenones, polyprenylated benzophenones
Mediterranean propolis	Greece, Malta, Crete, Southern Italy	*Cupressus* and *Pinus* plants, possibly *C. sempervirens*	Diterpenes, mainly acids of labdane type), anthraquinones
“Canarian” propolis	Canarian Islands	unknown	furoruran lignans
“Pacific” propolis	Okinawa, Taiwan, Japan	*Macaranga* plants, *Macaranga tanarius*	C-Prenyl-flavanones

**Table 9:** Anti-inflammatory and antioxidative properties of propolis and its flavonoids.

Effects of Propolis and Its Flavonoids on:	Mechanism
**ROS production**	- Ability to scavange ROS including singlet oxygen (^1^O_2_), hydroxyl radical (OH^●^), hydrogen peroxide (H_2_O_2_), superoxide anions (O_2_^●−^), perhydroxy radical (HO_2_^●^), lipid radical (LO^●^) and lipid peroxy radical (LOO^●^) - inhibition of oxidative enzymes - metal ion chelation (Cu^2+^, Fe^2+^, Zn^2+^ i Mg^2+^) - increase in the activity of antioxidant enzymes and their protection - synergistic action with other antioxidants - upregulate the signal pathway of (Nrf2-ARE) and an increase in transcriptional activity of protective genes such as antioxidant proteins and phase II detoxification enzymes
**RNS production**	- Ability to scavange nitric reactive radical (HOONO, NO, NO_3_ and others) - ability to inhibit NO production in LPS-induced macrophages and macrophages cell lines - inhibit iNOS protein and mRNA expression and reduce NO overproduction
**Eicosanoids’ production**	- Inhibition of the arachidonic acid signaling pathway and eicosanoid synthesis pathway: cyclooxygenase (COX) and lipoxygenase (LOX) - the COX-1 (constitutive form) pathway results in the synthesis of prostaglandins and thromboxanes, which are important for physiological functions - the COX-2 (inducible form) pathway plays a crucial rule in the production and release of inflammatory prostaglandins - the LOX pathway leads to the synthesis of leukotrienes and hydroxy eicosatetraenoic acid (HETE)
**Pro-oxidant reactive species**	- Inhibition of NADPH oxidase, xanthine oxidase and myeloperoxidase, COX, LOX
**Antioxidant Activity**	- Direct scavenging of ROS or RNS by hydrogen atom donation - inhibition of oxidases responsible for O_2_^●^^−^ production, xanthine oxidase, COX, LOX, microsomal monooxygenase, glutathione S-transferase, mitochondrial succinoxidase, and NADPH oxidase - activation of antioxidant enzymes involving the induction of phase II detoxifying enzymes, namely NAD(P)H-quinone oxidoreductase, glutathione S-transferase, and UDP-glucuronosyltransferase - chelating trace metals
**Endogeneus antioxidans**	- Sequestration of transition metal ions into complexes - scavenging or quenching of ROS and RNS - breaking chain reactions initiated by free radicals - repairing damaged molecules. - activation of the Nrf2/ARE pathway - ability to increase activity of SOD CAT and GPx enzymes - induce apoptosis of damaged cells
**Inactivation of pro-oxidant intermediates, ROS and RNS**	- Anti-mutagenic effect through the regulation of strong radicals - NO reacts with O_2_^●^^−^, producing ONOO^−^, cytotoxic and mutagenic peroxynitrous acid (ONOOH) - ONOOH becomes activated through a *cis*/*trans* isomerization producing *trans*-ONOOH, a powerful oxidant - highly reactive reaction between ONOO− and CO_2_, nitrosoperoxycarbonate anion (ONOOCO_2_^−^);decomposition of ONOOCO_2_—results in production of CO_2_ and NO_3_
**Anti-inflammatory effect**	***Anti-inflammatory effect of flavonoids is related to:*** Antioxidant activity - radical scavening - inhibition of ROS production - inhibition of prooxidative enzymes Modulation of inflammatory cells - modulation of enzymatic activity - modulation of secretory processes Modulation of proinflammatory enzymes - inhibition of arachidonic acid enzymes - inhibition NO synthase Modulation of proinflammatory mediators - modulation of cytokine production Modulation of pro-inflammatory gene expression - modulation of signal transduction - anti-inflammatory activity by bloking various signaling stages of the NLRP3 inflammasome pathway - inhibit the expression of NLRP3 inflammasome-related components such as IL-1β, IL-18, NLRP3, and caspase-1 and/or block inflammasome formation through modulation signaling molecules such as TLR4/NF-κB/NLRP3, PPARγ, TXNIP and Syk/Pyk2
**Cytokines production**	- Inhibitors of L-6, IL-1β, and TNF-*α,* IL-4, IL-13 formation in LPS-induced human monocytes, macrophages, gastric epithelial cells, and osteoblasts - Modulation of pro-inflammatory cytokines: INF-γ, TNF-α, IL-6, IL-1β, GM-CSF and IL-17A
**Chemokines production**	- Inhibition of IL-8, MIP-2 and MCP-1, CCL5 and CCL11 production,
**Transcription Factors**	- Inhibition of nuclear factor-κB (NF-κB), - mitogen-activated protein kinase (MAPK) - STAT activation
**Other Inflammatory Mediators**	***Effect of flavonoids is related to inhibition:***
*Growth factors*	- EGF; fibroblast growth factor; insulin-like growth factor 1; keratinocyte growth factor - hepatocyte growth factor; PDGF; and TGF-β
2. *Cell Adhesion Molecules*	- Cadherins, mucins, integrins, selectins, and Ig superfamily molecules - transendothelial migration of leukocytes, neutrophils and monocytes
3. *Platelet-Activating Factor;*	- Activation of platelets, chemoattraction and activation of neutrophils, macrophages and eosinophils, - bronchoconstriction, and increase in vascular permeability and vasodilation
4. *kinin;*	- Vascular permeability, vasodilation, hypotension, induction of pain, contraction of many types of smooth muscle cells, and activation of PLA2
5. *inflammasome;*	- Inflammasome formation
6. *C-Reactive Protein*	- Activation of the classical complement pathway and interaction with other cells
7. *Serum Amyloid A*	- Induces various cytokines, acts as a chemotactic for leukocytes, and stimulates angiogenesis, TF, and matrix MMPs’ expression
8. *Vasoactive Amines*	- Histamine and serotonin (main functions are vasodilation, increase venular permeability, enhance leukocyte rolling and firm adhesion, induction of gaps in the endothelial cell lining, and enhance leukocyte extravasation)
9. *Proteases*	- Cysteine proteases, serine proteases, and MMPs - degradation of the extracellular matrix - prepare the tissue for growth of new blood vessels and adherence and retention of newly recruited cells.

**Table 10:** Effects of inflammatory mediators on MCC, ciliary beat frequency, and mucus secretion * and modulation effects of propolis and its flavonoids on inflammatory mediators.

Inflammatory Mediators	MCC	Ciliary Beat Frequency	Mucus Secretion	Effect of Flavonoids on Inflammatory Mediators
Histamine	↑	↑	↑	↓
Acetylcholine	↑	↑(↓)	↑	↓
SRS-A	↓			
15-Hydroxyeicosa tetraenoic acid			↑↑	↓
Leukotriene C4		↑↑	↑↑	↓
Leukotriene D4	↓	↑	↑↑	↓
Bradykinin	↑		↑	↓
Thromboxane A2	↓	↑		↓
PAF	↓	↓	↑	↓
Eosinophil major basic protein		↓	↓	↓
Eosinophil cationic protein			↑	↓
Neutrophil elastase	↓	↓	↑	↓
Neutral protease		↓		↓
Mast cell chymase			↑	↓
PGs	↑	↑	↑	↓
VIP		↑	↑(↓)	↓
Neurokinin A		↑	↑	↓
Substance P		↑	↑	↓
Adenosine		↓		↓
Capsaicin			↑	↓
Cathepsin G			↑	↓
Complement C3a and C5		↓		↓
Endothelin 1, 2, and 3		↑	↑(↓)	↓

* ↑ = increase; ↓ = decrease; ↑(↓) = increase with some reported decreasing effect or no effect. PAF, platelet activating factor; VIP, vasoactive intestinal peptide.

## Data Availability

Not applicable.
